# Supplementary material for: Mixed-methods, descriptive and observational cohort study examining feeding and growth patterns among low birthweight infants in India, Malawi and Tanzania: the LIFE study protocol
Source: BMJ Open. 2021 Dec 2;11(12):e048216. doi: 10.1136/bmjopen-2020-048216 (PMC8640640; doi:10.1136/bmjopen-2020-048216)
Supplement: Author's manuscript [file bmjopen-2020-048216.draft_revisions.pdf]

BMJ Open

BMJ Open is committed to open peer review. As part of this commitment we make the peer review history of every article we publish publicly available.

When an article is published we post the peer reviewers' comments and the authors' responses online. We also post the versions of the paper that were used during peer review. These are the versions that the peer review comments apply to.

The versions of the paper that follow are the versions that were submitted during the peer review process. They are not the versions of record or the final published versions. They should not be cited or distributed as the published version of this manuscript.

BMJ Open is an open access journal and the full, final, typeset and author-corrected version of record of the manuscript is available on our site with no access controls, subscription charges or pay-per-view fees (http://bmjopen.bmj.com).

If you have any questions on BMJ Open's open peer review process please email info.bmjopen@bmj.com

BMJ Open

Low Birthweight Infant Feeding Exploration Study in India, Malawi and Tanzania: Protocol for a Mixed-Methods Descriptive Study

Journal:	*BMJ Open*
Manuscript ID	bmjopen-2020-048216
Article Type:	Protocol
Date Submitted by the Author:	19-Dec-2020

Complete List of Authors:	Vesel, Linda; Ariadne Labs at Brigham and Women's Hospital and the Harvard T.H. Chan School of Public Health<br>Spigel, Lauren; Ariadne Labs at Brigham and Women's Hospital and the Harvard T.H. Chan School of Public Health<br>Behera , Jnanindra ; SCB Medical College and Hospital, Department of Paediatrics,<br>Bellad, Roopa; KLE Academy of Higher Education and Research<br>Das, Leena; SCB Medical College and Hospital, Department of Paediatrics<br>Dhaded, Sangappa; KLE Academy of Higher Education and Research<br>Goudar, Shivaprasad S.; KLE Academy of Higher Education and Research<br>Guruprasad , Gowdar; JJM Medical College, Department of Neonatology<br>Misra, Sujata; SCB Medical College and Hospital, Department of Paediatrics<br>Panda, Sanghamitra ; City Hospital , Department of Obstetrics and Gynaecology<br>Shamanur , Latha; SS Institute of Medical Sciences and Research Center, Department of Paediatrics<br>Vernekar , Sunil; KLE Academy of Higher Education and Research<br>Hoffman , Irving ; University of North Carolina at Chapel Hill School of Medicine, Institute for Global Health and Infectious Diseases<br>Mvalo, Tisungane; UNC Project; University of North Carolina at Chapel Hill School of Medicine, Department of Pediatrics<br>Phiri, Melda; UNC Project<br>Saidi, Friday; UNC Project<br>Kisenge, Rodrick; Muhimbili University of Health and Allied Sciences, Department of Pediatrics and Child Health<br>Manji, Karim; Muhimbili University of Health and Allied Sciences, Department of Pediatrics and Child Health<br>Salim, Nahya; Muhimbili University of Health and Allied Sciences, Department of Pediatrics and Child Health<br>Somji, Sarah; Muhimbili University of Health and Allied Sciences, Department of Pediatrics and Child Health<br>Sudfeld, CR; Harvard University T H Chan School of Public Health, Departments of Global Health and Population<br>Adair, Linda; University of North Carolina at Chapel Hill Gillings School of Global Public Health, Department of Nutrition<br>Caruso, Bethany; Emory University School of Public Health, Hubert Department of Global Health<br>Duggan, Christopher; Boston Children's Hospital, Center for Nutrition

	Israel-Ballard, Kiersten; PATH, Maternal, Newborn, Child Health and Nutrition Program<br>Lee, Anne; Brigham and Women's Hospital, Pediatric Newborn Medicine<br>Martin, Stephanie; University of North Carolina at Chapel Hill Gillings School of Global Public Health, Department of Nutrition<br>Mansen , Kimberly; PATH, Maternal, Newborn, Child Health and Nutrition Program<br>North, Krysten; University of North Carolina at Chapel Hill School of Medicine, Department of Pediatrics<br>Young, Melissa; Emory University School of Public Health, Hubert Department of Global Health<br>Benotti, Emily; Ariadne Labs at Brigham and Women's Hospital and the Harvard T.H. Chan School of Public Health<br>Marx Delaney , Megan; Ariadne Labs at Brigham and Women's Hospital and the Harvard T.H. Chan School of Public Health<br>Fishman, Eliza; Ariadne Labs at Brigham and Women's Hospital and the Harvard T.H. Chan School of Public Health<br>Fleming, Katelyn; Ariadne Labs at Brigham and Women's Hospital and the Harvard T.H. Chan School of Public Health<br>Henrich, Natalie ; Ariadne Labs at Brigham and Women's Hospital and the Harvard T.H. Chan School of Public Health<br>Miller, Kate; Ariadne Labs at Brigham and Women's Hospital and the Harvard T.H. Chan School of Public Health<br>Subramanian , Laura ; Ariadne Labs at Brigham and Women's Hospital and the Harvard T.H. Chan School of Public Health<br>Tuller, Danielle; Ariadne Labs at Brigham and Women's Hospital and the Harvard T.H. Chan School of Public Health<br>Semrau, Katherine; Ariadne Labs at Brigham and Women's Hospital and the Harvard T.H. Chan School of Public Health; Harvard Medical School, Department of Medicine
Keywords:	EPIDEMIOLOGY, NEONATOLOGY, NUTRITION & DIETETICS, PUBLIC HEALTH, QUALITATIVE RESEARCH

SCHOLARONE™
Manuscripts

I, the Submitting Author has the right to grant and does grant on behalf of all authors of the Work (as defined in the below author licence), an exclusive licence and/or a non-exclusive licence for contributions from authors who are: i) UK Crown employees; ii) where BMJ has agreed a CC-BY licence shall apply, and/or iii) in accordance with the terms applicable for US Federal Government officers or employees acting as part of their official duties; on a worldwide, perpetual, irrevocable, royalty-free basis to BMJ Publishing Group Ltd ("BMJ") its licensees and where the relevant Journal is co-owned by BMJ to the co-owners of the Journal, to publish the Work in this journal and any other BMJ products and to exploit all rights, as set out in our licence.

The Submitting Author accepts and understands that any supply made under these terms is made by BMJ to the Submitting Author unless you are acting as an employee on behalf of your employer or a postgraduate student of an affiliated institution which is paying any applicable article publishing charge ("APC") for Open Access articles. Where the Submitting Author wishes to make the Work available on an Open Access basis (and intends to pay the relevant APC), the terms of reuse of such Open Access shall be governed by a Creative Commons licence – details of these licences and which Creative Commons licence will apply to this Work are set out in our licence referred to above.

Other than as permitted in any relevant BMJ Author's Self Archiving Policies, I confirm this Work has not been accepted for publication elsewhere, is not being considered for publication elsewhere and does not duplicate material already published. I confirm all authors consent to publication of this Work and authorise the granting of this licence.

For peer review only

Low Birthweight Infant Feeding Exploration Study in India, Malawi and Tanzania: Protocol for a Mixed-Methods Descriptive Study

Linda Vesel[1], Lauren Spigel[1], Jnanindra Nath Behera[2], Roopa M Bellad[3], Leena Das[2], Sangappa M Dhaded[3], Shivaprasad S Goudar[3], Gowdar Guruprasad[4], Sujata Misra[2], Sanghamitra Panda[5], Latha G Shamanur[6], Sunil S Vernekar[3], Irving F Hoffman[7], Tisungane Mvalo[8,9], Melda Phiri[8], Friday Saidi[8], Rodrick Kisenge[10], Karim Manji[10], Nahya Salim[10], Sarah S Somji[10], Christopher R Sudfeld[11], Linda S Adair[12], Bethany A Caruso[13], Christopher P Duggan[14], Kiersten Israel-Ballard[15], Anne CC Lee[16], Stephanie L Martin[12], Kimberly L Mansen[15], Krysten North[9], Melissa F Young[13], Emily Benotti[1], Megan Marx Delaney[1], Eliza Fishman[1], Katelyn Fleming[1], Natalie Henrich[1], Kate Miller[1], Laura Subramanian[1], Danielle E Tuller[1], Katherine E. A. Semrau[1,17]

[1] Ariadne Labs at Brigham and Women's Hospital and the Harvard T.H. Chan School of Public Health, Boston, Massachusetts, USA
[2] Department of Paediatrics, SCB Medical College, Cuttack, Odisha, India
[3] KLE Academy of Higher Education and Research's Jawaharlal Nehru Medical College, Belgaum, Karnataka, India
[4] Department of Neonatology, JJM Medical College, Davangere, Karnataka, India
[5] Department of Obstetrics and Gynaecology, City Hospital, Cuttack, Odisha, India
[6] Department of Pediatrics, SS Institute of Medical Sciences, Davangere, Karnataka, India
[7] Institute for Global Health and Infectious Diseases, University of North Carolina School of Medicine, University of North Carolina at Chapel Hill, Chapel Hill, North Carolina, USA
[8] University of North Carolina Project Malawi, Lilongwe, Malawi
[9] Department of Pediatrics, School of Medicine, University of North Carolina at Chapel Hill, NC, USA
[10] Department of Pediatrics and Child Health, Muhimbili University of Health and Allied Sciences, Dar es Salaam, Tanzania
[11] Departments of Global Health and Population and Nutrition, Harvard T.H. Chan School of Public Health, Boston, MA, USA
[12] Department of Nutrition, Gillings School of Global Public Health, University of North Carolina at Chapel Hill, Chapel Hill, NC, USA
[13] Hubert Department of Global Health, Emory University Rollins School of Public Health, Atlanta, GA, USA
[14] Center for Nutrition, Boston Children's Hospital, Boston, MA, USA
[15] Maternal, Newborn, Child Health and Nutrition Program, PATH, Seattle, WA, USA
[16] Department of Pediatric Newborn Medicine, Brigham and Women's Hospital, Boston, MA, USA
[17] Department of Medicine, Harvard Medical School. Boston, MA, USA

Corresponding Author: Linda Vesel, 401 Park Drive, 3rd Floor, Boston, MA 02215, USA, lvesel@ariadnelabs.org

Word count (excluding title page, abstract, references, figures and tables): 3657

For peer review only - http://bmjopen.bmj.com/site/about/guidelines.xhtml

ABSTRACT

Introduction

Ending preventable deaths of newborns and children under five will not be possible without evidence-based strategies addressing the health and care of low birthweight (LBW <2.5kg) infants. The majority of LBW infants are born in low- and middle-income countries (LMICs) and account for more than 60-80% of newborn deaths. Feeding promotion tailored to meet the nutritional needs of LBW infants in LMICs and prevent growth faltering is crucial for curbing newborn mortality rates. The Low Birthweight Infant Feeding Exploration (LIFE) Study aims to establish foundational knowledge about optimal feeding options for LBW infants in low-resource settings throughout infancy.

Methods and analysis

This mixed-methods, descriptive study in India, Malawi and Tanzania consists of desk reviews, facility needs assessments, retrospective chart reviews, in-depth interviews, focus group discussions, observations of infant care and feeding, and longitudinal cohort surveys with anthropometric measures captured over time for LBW infants with birthweights of 1.5kg to <2.5kg. Study measures include optimal feeding, morbidity, mortality, well-being, child development, and health system inputs that facilitate or hinder care of LBW infants.

Ethics and dissemination

This study was approved by eleven ethics committees in India, Malawi, Tanzania and the United States. The results will be disseminated through peer-reviewed publications and presentations targeting the global and local research, clinical, program implementation and policy communities.

Registration details

Clinical Trials Registration: NCT04002908 (www.clinicaltrials.gov) and CTRI/2019/02/017475 (Clinical Trial Registry of India - http://ctri.nic.in).

STRENGTHS & LIMITATIONS OF THIS STUDY

- Our mixed methods study design will yield detailed and unique data on supply and demand side aspects of infant care, feeding and growth at the facility and community levels throughout the first year of life.
- The LIFE study will focus on low birthweight (LBW) infants with birthweights between 1.5kg and 2.5kg since there is limited data on this more predominant group of LBW infants.
- The multi-site approach will enable the comparison of results across multiple contexts within and across 3 countries, representing populations in Sub-Saharan Africa and South Asia where manifestations of LBW infants may vary.
- The main limitation of this study is that enrollment is facility-based, missing the population of small infants who are born outside the facility or delay presentation for care within the health system.

KEYWORDS

Low birthweight; nutrition; growth; anthropometrics; newborn; infant; feeding; morbidity; mortality; India; Malawi; Tanzania

For peer review only - http://bmjopen.bmj.com/site/about/guidelines.xhtml

INTRODUCTION

Challenge

Although improvements in child health and survival have been achieved in the past two decades, a significant decline in neonatal mortality is needed to attain Sustainable Development Goal 3.2 by 2030 (1). Focusing attention on the most vulnerable small and sick newborns, namely those born low birthweight (LBW <2.5 kg), can reduce risk and poor outcomes in the first days, weeks and months of life. LBW, resulting from preterm birth and/or intrauterine growth restriction, accounts for 15.5% of newborn births, but represents 60-80% of newborn deaths (2). This burden is disproportionately concentrated in low- and middle-income countries (LMICs) (2). For at least the last five years, prematurity has been the predominant cause of newborn mortality (3). Mortality risk among infants born preterm and small for gestational age is more than 15 times greater than those born term and with adequate size for gestational age (2,4–7).

Additionally, LBW infants face increased risks for morbidity, including infections, neurodevelopmental impairments, and growth faltering as well as challenges related to breastfeeding, particularly its initiation and exclusivity in early infancy (2,8–12). Inadequate and insufficient feeding contributes to poor growth outcomes such as stunting, wasting and underweight, for which the risk among LBW infants is already heightened (13). Without close monitoring of growth and guidance on optimal feeding, LBW infants may not catch up to their normal birthweight and term peers within a healthy time period.

Design, testing, and implementation of interventions to optimize feeding, growth and development among LBW infants require a rigorously investigated foundational understanding of current practices, standard of care, outcomes and resource availability in LMICs (14). Limited evidence exists, particularly in low resource settings, on feeding patterns and initiation, the feeding ecosystem, policies for the care of LBW infants, and supply and demand inputs related to various feeding modes (15–17). Generally, studies capturing evidence on nutritional interventions for LBW infants are lacking hindering the proper management of nutritionally at-risk newborns (18). World Health Organization (WHO) feeding guidelines for LBW infants were published in 2011 (19); however, most of the recommendations are based on low quality evidence, the majority were from high income countries, and most were limited to very LBW infants (<1.5kg) even though the majority of LBW infants are born between 1.5kg and 2.5kg (12,20).

Opportunity

Despite these gaps, a number of opportunities in recent years have propelled interest and investment in the care of LBW infants. In 2012, the World Health Assembly highlighted the need to prevent LBW by setting a goal of reducing LBW births by 30% by 2025 as part of a set of six nutrition targets comprising its comprehensive implementation plan on maternal, infant and young child nutrition (2). In 2017, 40 global health organizations issued an urgent call to action for more evidence to address the current state of feeding of sick and vulnerable infants (21). In the past two years, the WHO, UNICEF and USAID released reports and guidelines focusing on the care of the small and sick newborn (22). With interest and investment galvanized, research is needed to develop evidence-based approaches and translate findings into action to ensure the most vulnerable infants can thrive.

For peer review only - http://bmjopen.bmj.com/site/about/guidelines.xhtml

Study Aims

The Low Birthweight Infant Feeding Exploration (LIFE) study will aim to document current feeding practices and growth patterns among LBW infants in LMICs to inform potential feeding interventions. The study will have four objectives to be assessed among LBW infants: 1) understand feeding practices and the standard of care underpinning them; 2) define and document key longitudinal growth and health outcomes up to 12 months of age; 3) examine predictors of growth faltering; and 4) assess acceptability and feasibility of a system-level Infant and Young Child Feeding (IYCF) intervention with proposed feeding options or packages. The long-term goal of the LIFE study is to build the evidence-based needed to design and rigorously test a future LBW infant feeding and growth trial and, in turn, revise global infant feeding guidelines.

METHODS AND ANALYSIS

Study design

LIFE will be a multi-site, mixed-methods descriptive study with several data collection streams including desk reviews; facility needs assessments; quantitative data collection utilizing retrospective chart reviews, observations, longitudinal infant follow-up and surveys; and qualitative data collection involving in-depth interviews (IDIs) and focus group discussions (FGDs) (Table 1). Data collection began in August 2019 and is on-going with plans for completion by October 2021.

Table 1. Study activities and samples

Data source		Study Objective*	Aim	Study population	N (per site)
Desk review	Literature review	1	Document evidence from published research and policies/protocols/guidelines on LBW infant feeding to promote optimal growth, growth patterns, and use of breastmilk substitutes and strategies for nutritionally at-risk infants.	N/A	N/A
	Umbrella review	1	Conduct a formal systematic review of systematic reviews to document the landscape of evidence on various sources of nutrition for LBW infants and their association with growth.	N/A	N/A
Facility needs assessment	Facility profile	1, 4	Examine health system inputs related to LBW infant feeding and understand facility readiness to care for LBW infants.	Study facilities	2-5
	DHM readiness assessment	1, 4	Collect information needed to inform future research activities that may utilize existing donor human milk (DHM) banks or establish new ones.	Study facilities	1-3
Quantitative data	Retrospective chart review	1, 4	Document the routine and standard of care for LBW infants at each study facility for a cohort of infants prior to longitudinal data collection.	Mother-infant pairs	155
	Prospective cohort	1, 2, 3, 4	Understand feeding practices among LBW infants, their growth and their health outcomes over the first 12 months of life.	Mother-infant pairs	300

For peer review only - http://bmjopen.bmj.com/site/about/guidelines.xhtml

	In-facility observation cohort	1, 2, 4	Examine early maternal lactation and feeding practices among LBW infants from birth to facility discharge.	Mother-infant pairs	35
Qualitative data	In-depth interviews	1, 4	Explore beliefs, norms, barriers and facilitators related to LBW infant feeding.	Clinicians	Up to 40
				Government officials and supply chain and DHM knowledge experts	Up to 12
				Mothers	Up to 20
	Focus group discussions	1, 4	Explore beliefs, norms, barriers and facilitators related to LBW infant feeding.	Mothers	Up to 4 (5-8 participants)
				Family members	Up to 4 (5-8 participants)
				Community members	Up to 4 (5-8 participants)

*Study objectives: (1) Understand feeding practices and the standard of care underpinning them; (2) define and document key longitudinal growth and health outcomes up to 12 months of age; (3) examine predictors of growth faltering; and (4) assess acceptability and feasibility of a system-level IYCF intervention with proposed feeding options or packages.

Study setting

The LIFE study will be conducted in four sites across three countries: 1) Karnataka State, India and 2) Odisha State, India led by teams at Jawaharlal Nehru Medical College, JJM Medical College, SS Institute of Medical Sciences, City Hospital and SCB Medical College; 3) Lilongwe, Malawi led by the team at UNC Project Malawi; and (4) Dar-es-Salaam, Tanzania led by the team at Muhimbili University of Health and Allied Sciences. All sites have strong, long-standing relationships with key government stakeholders, placing them in a position to address local and national priorities and advocate for translation of research to practice. Sites were chosen in South Asia and sub-Saharan Africa as these regions represent the greatest burden of LBW as well as its diverse drivers (23). Investigators will document regional similarities and differences regarding gestational age, birthweight, feeding patterns, and growth outcomes. In total, participants will be recruited from 12 study facilities (i.e. hospitals) chosen based on delivery volume, capacity to care for LBW infants in the first days of life, and willingness of facility leadership to participate. All facilities are located in urban settings with participants residing within a 50 kilometer radius. Additional site details are included in Table 2.

Table 2. Site descriptions

**Site**	**Prevalence of LBW (24) (2)**	**Neonatal Mortality Rate (25)*Deaths per 100 live births***	**Infant Mortality Rate - *Deaths per 100 live births (26)***	**Study facilities per site and type**
India-Karnataka State	17.2%	22	28	3 private tertiary hospitals<br>2 public tertiary hospitals
India-Odisha State	20.8%			1 public tertiary hospital<br>1 public secondary hospital

For peer review only - http://bmjopen.bmj.com/site/about/guidelines.xhtml

Malawi	14.5%	20	31	1 public tertiary hospital<br>1 public secondary hospital
Tanzania	10.5%	20	36	1 public tertiary hospital<br>2 public secondary hospitals

Study population

For each data stream involving the recruitment of mothers, infants or interview respondents, participants will need to fulfill specific inclusion criteria detailed in Table 3. Mothers and their newborns with recorded birthweights of 1.5kg to <2.5kg will be eligible for the chart review, prospective cohort, in-facility observation cohort, IDIs and FGDs. Maternal consent will be required to participate. Additionally, IDIs and FGDs will be conducted among other stakeholders involved in IYCF, including family members (e.g. husband, guardian, mothers' parents, mother's in-laws, grandmother, sister, and sister-in-law), religious and community leaders, traditional healers, clinicians, government officials, supply chain experts, and human milk banking experts. Clinicians who have been at their position for less than six months will be excluded from IDIs and FGDs. All 12 study facilities will participate in at least one of the facility needs assessments, namely a facility profile and a donor human milk (DHM) bank assessment; further inclusion and exclusion criteria have not been specified. Similarly, the desk reviews will capture evidence on the care and feeding of LBW infants without specific linkage to mother-infant pairs evaluated as part of the LIFE study.

Table 3. Inclusion and exclusion criteria mothers-infant pairs

Data Stream	Inclusion Criteria	Exclusion Criteria
Retrospective Chart Review	• Infants with birthweight of 1.5kg to <2.5kg<br>• Infants discharged before the start of prospective data collection LIFE	• Infants with birthweight <1.5kg<br>• Infants with congenital abnormalities that interfere with feeding (cleft lip or palate; hydrocephalus; gastrointestinal tract anomalies including gastroschisis, omphalocele, or anal atresia; neural tube defects; congenital cardiac defects; suspected trisomy 21; suspected TORCH infection (Toxoplasmosis, Other agents, Rubella, Cytomegalovirus, and Herpes simplex)<br>• Infants with young mothers: <18 years old in Tanzania and India, 16-17 years old and unmarried in Malawi, and all mothers <16 years old in Malawi<br>• Infants who die less than 72 hours from the time of birth<br>• Infants born outside the facility
Prospective Longitudinal Survey	• Infants with birthweight between 1.5kg to <2.5kg (as measured at birth, or calculated using algorithm based on time since birth to account for expected postnatal weight loss)<br>• Mother-infant pairs who reside within the catchment area (approximately 50km) of the facility in which they were enrolled	• Infants with birthweight <1.5kg<br>• Infants with congenital abnormalities that interfere with feeding (cleft lip or palate; hydrocephalus; gastrointestinal tract anomalies including gastroschisis, omphalocele, or anal atresia; neural tube defects; congenital cardiac defects; suspected trisomy 21; suspected TORCH infection (Toxoplasmosis, Other agents, Rubella, Cytomegalovirus, and Herpes simplex). Infants with severe neonatal encephalopathy jeopardizing early survival (as determined by modified Sarnat criteria) (27,28)<br>• Infants with young mothers: <18 years old in Tanzania and India, 16-17 years old and

For peer review only - http://bmjopen.bmj.com/site/about/guidelines.xhtml

		unmarried in Malawi, and all mothers <16 years old in Malawi<br>• Infants with mothers who died prior to enrollment<br>• Infants who die less than 72 hours from the time of birth<br>• Infants older than 72 hours at the time of screening<br>• Infants who withdraw less than 72 hours from the time of birth<br>• Infants with a twin or triplet who die prior to the time of screening<br>• Mothers who plan to leave the catchment area within 6 months of study enrollment
Prospective In-facility Observations	• Infants with birthweight between 1.5kg to <2.5kg	• Infants with birthweight <1.5kg<br>• Infants with congenital abnormalities that interfere with feeding<br>• Infants with young mothers: <18 years old in Tanzania and India, 16-17 years old and unmarried in Malawi, and all mothers <16 years old in Malawi<br>• Infants who die less than 6 hours from the time of birth<br>• Infants with mothers who die less than 6 hours from the time of birth<br>• Infants born outside the facility<br>• Infants older than 6 hours at the time of screening
IDIs and FGDs (Birth - 6 months)	• Mothers with infants with birthweight between 1.5kg to <2.5kg aged 0 to 7 months (enrollment in prospective cohort not required)<br>• Family members of infants with birthweight between 1.5kg to <2.5kg aged 0 to 7 months who play a role in infant and young child feeding<br>• Religious leaders, community leaders, and traditional healers that are opinion leaders on infant and young child feeding practices in the community<br>• Health care workers currently involved in providing infant and young child feeding<br>• Government officials who support infant and young child feeding programs and policies<br>• Supply chain experts involved in infant and young child feeding supply chain logistics<br>• Human milk bank experts	• Mothers and family members with infants with birthweight ≥2.5kg<br>• Mothers and family members with infants with birthweight <1.5kg<br>• Young mothers: <18 years old in Tanzania and India, 16-17 years old and unmarried in Malawi, and all mothers <16 years old in Malawi<br>• Health care workers who have been in their position for less than 6 months<br>• Government officials who have been at their post for less than 6 months
IDIs (9-12 months)	• Mothers with infants with birthweight between 1.5kg to <2.5kg enrolled in the prospective cohort and aged 9-12 months	• Mothers with infants who were not enrolled in the prospective cohort or withdrew/died before 9 months of age

For peer review only

Patient and public involvement

As part of the study design, the LIFE study team will involve clinicians and researchers familiar with the respective settings and populations. Additionally, a community advisory board in Malawi will be consulted. Study tools will be piloted with patients and community members to ensure that research questions and indicators will be culturally appropriate, acceptable and relevant to the study population.

For peer review only - http://bmjopen.bmj.com/site/about/guidelines.xhtml

Study measures

A range of study measures will be evaluated to address our study objectives. As part of objective one - to understand feeding practices and the standard of care underpinning them, we will examine infant feeding patterns including: timing of feeding initiation, feeding types given from birth to 12 months, and duration of exclusive breastfeeding. For objective two - to define and document key longitudinal growth and birth outcomes, we will measure infant weight, length, head circumference and mid-upper arm circumference at multiple timepoints to assess growth faltering. Due to a lack of consensus and standardization of the definition and evaluation of growth faltering (29), we will be using various indicators and strategies including: inability to regain birthweight in the first two weeks of life; slow weight gain in the first 6 weeks of life (≤10 grams per day for two consecutive weeks and <20 grams per day); slow weight gain between 10 and 18 weeks of life (change in weight-for-age z-score >0.67 SD), stunting (length-for-age z-score <-2SD), wasting (weight-for-length z-score <-2SD) and underweight (weight-for-age z-score <-2SD) at six and 12 months; and growth trajectories and velocities (weight-for-age z-score, length-for-age z-score and weight-for-length z-score) to depict trends of growth over time against an appropriate standard of optimal growth (i.e. the INTERGROWTH-21st newborn size at birth and preterm postnatal growth standards (30,31) and the WHO infant growth standards (32)). The WHO standards were designed using a cohort of term infants while the INTERGROWTH-21st standards were designed specifically for preterm infants and serve as a complement to the WHO standards in the first six months of life. Additional study measures for objective two include infant morbidity at six and 12 months, infant mortality throughout infancy and child development at one year. Under objective three - to examine predictors of infant growth faltering, we will use previously mentioned growth faltering indicators as well as select maternal and infant characteristics, feeding patterns over time and maternal health and well-being. Finally, to address objective four - to assess the acceptability and feasibility of IYCF interventions with proposed feeding options or packages, we will explore barriers, facilitators, risks and benefits of various feeding practices and link these insights with the growth and feeding results from objectives one to three to make targeted recommendations.

Data collection

Data Sources

Data collection will consist of desk reviews, facility needs assessments, quantitative surveys and observations, and qualitative interviews and discussions (Table 1). Across the data streams, initiation of data collection will vary based on timing of IRB approvals and other contextual factors. Timing of data collection will be linked with infant age; details vary by data stream (Figure 1). All data collection will be prospective except for the chart reviews. Desk reviews and facility needs assessments are not included in Figure 1 since they will not be linked to infant age and will be completed throughout the study.

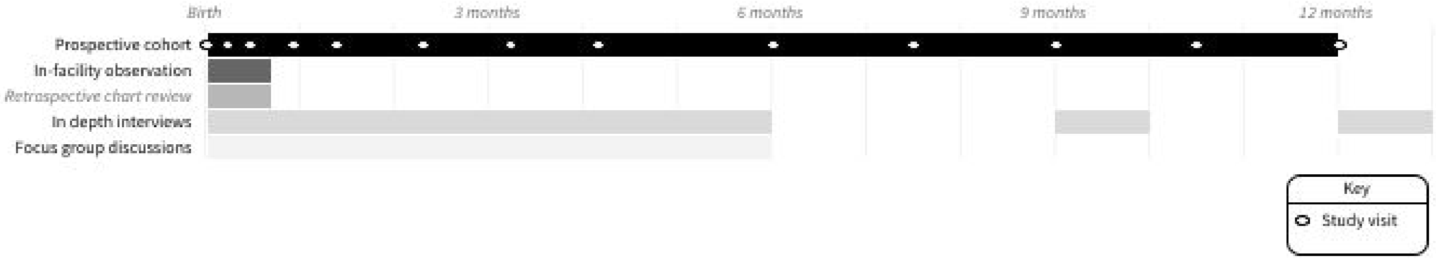

Figure 1. Data collection timeline by data source and infant age

For peer review only - http://bmjopen.bmj.com/site/about/guidelines.xhtml

Desk reviews

Desk reviews will involve searching and synthesizing current evidence and methodologies related to feeding and growth of LBW infants using PubMed, CINAHL, Embase, Web of Sciences and grey literature. For the umbrella review, the strength of evidence will be graded using established criteria.

Facility needs assessments

Facility needs assessment data will be collected via standardized tools including quantitative and qualitative questions. The facility profile tool will be designed to capture key vital statistics and describe the structural, human resource, equipment and service inputs present at each of study facilities within the obstetric, mother/baby (newborn), neonatal intensive care unit (NICU) or special newborn care unit (SNCU), and pediatric wards. It will be administered by a study team member at each facility through observations, record reviews and staff consultations. The DHM Readiness Assessment will be based on previously validated tools utilized in multiple LMICs to determine facility and programmatic readiness for establishing and strengthening DHM banks (33–36). The assessment will explore current lactation support, milk storage and transportation, infrastructure requirements for quality human milk bank systems, and regulatory and ethical considerations for the use of DHM. This assessment will be administered by study staff with the support of global DHM experts through consultations, observations and structured surveys.

Chart review

Research nurses will collect retrospective patient chart data for infants born between July 2018 and October 2019 using a structured survey capturing information on maternal and newborn demographics, feeding, growth, morbidity and documentation. Data collection in each site will be completed before prospective cohort data collection begins, varying slightly by site.

Prospective cohort

Research nurses will gather longitudinal, prospective data using structured survey tools and observations for infants born between September 2019 and January 2020 (noting that sites will start and end at different times) and followed up for 12 months until July 2021. For the prospective cohort, we will collect survey data at 13 timepoints between birth and 52 weeks of age (Figure 1 and Table 4). Data will be collected in-person at all timepoints, apart from 32.5 and 45.5 weeks, at which time an abbreviated survey will be administered over the phone in Malawi and Tanzania to bridge the three-month time gap between in-person visits and reduce loss to follow-up. The India sites will not conduct these two extra surveys based on past experience in previous studies with at least three-month intervals between visits without the need for check-ins. Data will be collected during hospitalizations, as applicable. Where possible, we will use survey items from validated instruments to develop our data collection tools. See Table 4 for the planned timing of administration for all assessments that will comprise data collection for the prospective cohort. Focused training will be conducted by a pediatrician or research investigator for all assessments listed. Although this is an exploratory study with no intervention, adverse events will be monitored and recorded.

For peer review only - http://bmjopen.bmj.com/site/about/guidelines.xhtml

Table 4. Timing of assessment in prospective cohort

Assessment or Survey Content	Age of Infant (weeks)												
	0	1	2	4	6	10	14	18	26	32.5	39	45.5	52
Maternal demographics and pregnancy history*	▪												
Infant demographics and delivery information*	▪												
Dubowitz examination for gestational age at birth (37,38)	▪												
Infant anthropometrics*	▪	▪	▪	▪	▪	▪	▪	▪	▪		▪		▪
Maternal anthropometrics	▪				▪				▪				▪
Maternal and infant health information	▪	▪	▪	▪	▪	▪	▪	▪	▪		▪		▪
Maternal and infant mortality information*	▪	▪	▪	▪	▪	▪	▪	▪	▪	▪	▪	▪	▪
The World Health Organization-5 Well-Being Index (WHO-5) (39)											▪		▪
Maternal lactation and infant feeding information*	▪	▪	▪	▪	▪	▪	▪	▪	▪	▪	▪	▪	▪
Infant and Young Child Feeding (IYCF) Questionnaire for complementary feeding period (40)											▪		▪
LATCH breastfeeding assessment* (41)		▪	▪	▪	▪	▪	▪	▪	▪				
Preterm Infant Breastfeeding Scale (PIBBS)* (42)		▪	▪	▪	▪								
Neonatal Eating Assessment Tool (NeoEAT) (43)		▪	▪	▪	▪	▪	▪	▪	▪				
Water, sanitation and hygiene information*		▪	▪	▪	▪	▪	▪	▪	▪		▪		▪

For peer review only - http://bmjopen.bmj.com/site/about/guidelines.xhtml

Patient Health Questionnaire 2 (PHQ-2) on maternal depression (44)		▪	▪	▪	▪	▪	▪	▪	▪		▪		▪
Caregiver Reported Early Childhood Development Instrument (CREDI) (45)													▪

*These assessments will also be completed for the in-facility observation cohort between birth and facility discharge.

The prospective cohort sample size of 300 mother-infant pairs was selected to be as large as possible given constraints in time, recruitment rates, and budget. Hypothesis testing using this observational data is not planned. The main statistical results from the prospective cohort will be point estimates and confidence intervals for certain rates, such as the percent of LBW infants who fail to thrive or whose growth falters, develop problems breastfeeding, or fall ill. The size of the true proportions will determine the precision of the estimate (e.g. with a sample of 300 mother-infant pairs, a true proportion of 10% can be detected with precision of +/- 3.6%). The 95% confidence interval would be 6.4% to 13.6% (Figure 2).

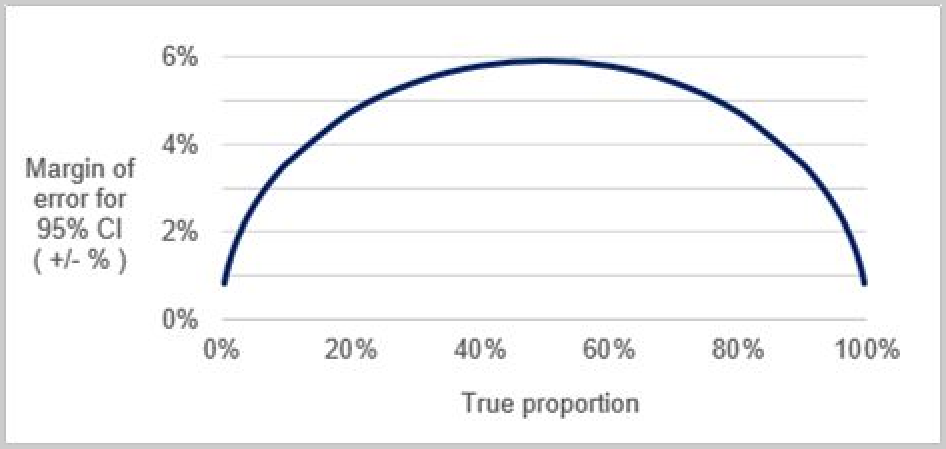

Figure 2: Margin of error in estimation of a single proportion with a sample of 300 for the prospective cohort

To ensure standardization and facilitate high quality data collection, investigators will conduct site-specific workshops prior to data collection to review data collection tools, provide guidance on piloting and train research staff in theory and practice of key assessments listed in Table 4. The timing and duration of each workshop will be tailored to the needs and knowledge of each team. Data collectors will be taught how to accurately collect standardized anthropometric measurements, and maintain and calibrate scales and infantometers. Supervisors will perform regular quality checks and one-on-one as well as group refresher training as needed. Standardized anthropometric equipment that will be used for the study will include: Seca 334 Mobile Digital Baby Scale, 887/876/874 Digital Flat Scale with Foot Pedal for Maternal Weight, Seca 417 Infant Measuring Board, Shorr MUAC tapes (WM-MUAC26), and Shorr Tape 65cm head circumference (SKU: WM-STape).

In-facility observation cohort

For the in-facility observational cohort, research nurses will conduct regular observations and surveys within six hours of birth through facility discharge (first week - every three to four hours; second week - one to two times a day; and third week and thereafter - once daily for unstable infants and every three days for stable infants). The feeding observations will span about 15 minutes. See Table 4 for assessments and survey

For peer review only - http://bmjopen.bmj.com/site/about/guidelines.xhtml

content included in this data stream. Each site will conduct in-facility observations for 35 mother-infant pairs divided among the participating study facilities. This sample size was determined by timeline and feasibility constraints. We will not aim to make statistical inferences or precise point estimates from these samples, but will use the data descriptively for each infant to construct a narrative of their feeding experience at the facility.

Qualitative data

Trained local interviewers will conduct IDIs and FGDs with mothers and family members of LBW infants, community members, clinicians, government officials, and supply chain and DHM knowledge experts between July 2019 and January 2021. Mothers of infants aged seven months or less will be sampled for participation in the FGDs from patient charts based on the age of their infants (0-3 months and 4-7 months); these pairs will not need to be included in the prospective cohort. Conversely, mothers of infants aged nine to twelve months will be sampled from the prospective cohort based on their age (with a one month window), sex and growth status at six months (stunted and not). All other respondents will be interviewed based on their differing roles within IYCF support using convenience sampling based on availability. We will develop semi-structured interview guides, which will be piloted by site staff prior to use. IDIs and FGDs will be conducted in English or in the local language and will take place in a private location either within a study health facility or in the community setting. Participants will be given a small incentive to participate in order to cover the cost of travel and lost wages. All interviews and discussions will be audio-recorded and supported with real-time note-taking.

Data management and analysis

Facility needs assessments

Descriptive and thematic analyses will be conducted using Excel. Key messages will be elucidated and compared across study sites and geographies.

Chart review, prospective cohort and in-facility observation cohort

All data will be entered into CommCare, a secure electronic data capture and management system. For the prospective cohort only, the Tanzania team will use its own electronic data capture system and will securely transfer data to be merged with the rest of the data collected in CommCare. All data used for analysis will be de-identified and stores securely. We will use means, medians and standard deviations to describe continuous variables and frequencies and proportions for categorical variables. Confidence intervals around all measures will be constructed to take any clustering into account, by site or to account for twins. Any further analyses will be post-hoc and described separately. Analyses will be conducted using the Stata Statistical Software Package, Version 16 (46) and SAS software (47).

Qualitative data

All de-identified data will be stored in SharePoint. Analysis of the IDIs and FGDs will involve a combination of rapid (framework analysis based on real-time notes supported by audio-recordings where needed) and in-depth (coding of verbatim translated transcripts) approaches (48–50). In India and Malawi, IDIs and FGDs will be summarized into thematically structured tables, corresponding to the research questions, by interviewers at each site. All summary tables will undergo quality reviews and any questions or concerns will be addressed with the interviewers and discussed until consensus is reached. These tables will then be coded by qualitative researchers at Harvard using Dedoose (51). Framework analysis and coding of notes will be used for the first

For peer review only - http://bmjopen.bmj.com/site/about/guidelines.xhtml

For peer review only

stage of data collection in India and Malawi because it enables on-going learning during the data collection period for a large volume of qualitative data. For maternal IDIs conducted in the second half of infancy in India and Malawi, as well as all of Tanzania's maternal IDIs and FGDs across the full year of infancy, full transcripts will be coded in Dedoose by qualitative researchers at Harvard. A comprehensive, deductive analysis of the data will be conducted by the Ariadne Labs team to identify key messages for each theme, as well as similarities and differences across sites. The analyses for each theme will be reviewed with the corresponding site teams to ensure that the interpretation and characterization of the content is appropriate.

Protocol and registration

The study is registered with clinicaltrials.gov (NCT04002908) and CTRI/2019/02/017475 (Clinical Trial Registry of India - http://ctri.nic.in).

ETHICS & DISSEMINATION

This study was approved by eleven ethics committees in India, Malawi, Tanzania and the United States: 1) India Health Ministry's Screening Committee with Indian Council of Medical Research acting as its secretariat; 2) Directorate of Health and Family Welfare Services, Government of Karnataka which also covers investigators at Women & Children Hospital, Davangere and Chigateri General District Hospital, Davangere; 3) Institutional Ethics Committee of KLE Academy of Higher Education and Research which also covers investigators at JN Medical College, Belagavi and KLES Dr Prabhakar Kore Hospital & Medical Research Center, Belagavi; 4) Institutional Ethics Review Board of SS Institute of Medical Sciences & Research Centre; 5) Institutional Ethics Committee of JJM Medical College which also covers investigators at Bapuji Child Health Institute & Research Centre, Davangere, Women & Children Hospital, Davangere and Chigateri General District Hospital, Davangere; 6) Research and Ethics Committee, Directorate of Health Services, Odisha State which also covers investigators at City Hospital Oriya Bazar, Cuttack; 7) Institutional Ethical Committee, Sriram Chandra Bhanja Medical College, Cuttack; 8) Malawi National Health Sciences Research Committee; 9) Tanzania National Institute of Medical Research; 10) Muhimbili University of Health and Allied Sciences; and 11) United States - Harvard T.H Chan School of Public Health which also covers investigators at Boston Children's Hospital, Brigham and Women's Hospital, Emory University, PATH and University of North Carolina.

Written informed consent will be obtained from all IDI, FGD, in-facility observation cohort and prospective cohort participants**.** After six months of follow-up, prospective cohort participants will be re-consented for an additional six months. Verbal consent will only be obtained in the event of extenuating circumstances where written consent cannot be sought; IRB approval will be needed before proceeding. For the facility needs assessments, verbal consent will be sought at the facility leadership level as these activities are considered to be a part of quality improvement. Results from the LIFE study will be disseminated at global- and site-levels through peer-reviewed publications and presentations to key stakeholders during meetings and conferences. Site-PIs will also share results informally with participating study facilities. Upon publication of the study results, data will be uploaded to Harvard Dataverse.

DISCUSSION

The LIFE study will fill critical data gaps in LBW care and nutrition in LMICs. Through systematic identification of context-specific practices and their barriers and enablers, we will aim to establish the foundational knowledge required to design and test the most effective and feasible infant feeding strategies appropriate for LBW infants in low resource settings. Our hope is that the findings from this rich, mixed methods study will

For peer review only - http://bmjopen.bmj.com/site/about/guidelines.xhtml

enable us to learn how to support breastfeeding and provision of mother's own milk; understand how LBW infants grow; identify the predictors of growth faltering; and explore the role of breastmilk substitutes and supplements to prevent and support infants from becoming nutritionally at-risk in the first year of life. This work will provide much-needed evidence to inform future trials and revisions to global LBW infant feeding guidelines.

ACKNOWLEDGEMENTS

The authors would like to thank clinical leadership and staff at all study facilities for their buy-in, support and contribution to this work; the mothers and infants for allowing us to have a glimpse into their experiences and sharing key moments of their lives; community members, government officials and subject experts for sharing their perspectives; and all data collectors and study staff for conducting study activities.

AUTHORS' CONTRIBUTIONS

Study conceptualization and design was completed by ACL, BC, CD, CRS, DET, EB, IH, KEAS, KIB, KLM, KM, KMi, KN, LA, LSp, LSu, LV, MFY, NH, RMB, SMD, SSG and TM. Protocol development and tool design was carried out by ACL, BC, CRS, DET, EB, EF, GG, IH, JNB, KEAS, KF, KIB, KLM, KM, KMi, KN, LA, LD, LGS, LSp, LSu, LV, MFY, MMD, NH, NS, RK, RMB, SLM, SM, SP, SMD, SSG, SSS, SSV and TM. Tool pilot testing/ modification were conducted by EB, EF, FS, GG, JNB, KF, LD, LGS, LSp, LSu, LV, MP, NH, NS, SM, SP, SSS and SSV. Writing of the manuscript was conducted by LSp and LV. All authors reviewed multiple drafts of the manuscript and approved the final version.

FUNDING STATEMENT

This work was supported by The Bill & Melinda Gates Foundation, grant number OPP1192260. The Bill and Melinda Gates Foundation reviewed the study design and sample-size calculations, but will not be involved in data collection, management, analysis, interpretation, writing of the manuscript, or the decision to submit manuscripts for publication.

COMPETING INTERESTS STATEMENT

All authors completed the ICMJE conflict of interest form and were funded by the Gates Foundation for this work as part of the LIFE study. ACL, BC, CRS, DET, KEAS, KIB, KLM, KM, MMD, RK and SS have received funding from the Gates Foundation for maternal and newborn health work at large. CD reports other from American Society for Nutrition, other from UpToDate, and other from People's Medical Publishing House outside the submitted work. ACL reports grants from the World Health Organization and National Institute of Health NICHD outside the submitted work. All other authors have declared no conflicts of interest.

REFERENCES

1. Goal 3: Ensure healthy lives and promote well-being for all at all ages — SDG Indicators. United Nations - SDG; 2017.
2. Blencowe H, Krasevec J, de Onis M, Black RE, An X, Stevens GA, et al. National, regional, and worldwide estimates of low birthweight in 2015, with trends from 2000: a systematic analysis. Lancet Glob Health. 2019 May 15;7(7):e849–60.
3. Liu L, Oza S, Hogan D, Chu Y, Perin J, Zhu J, et al. Global, regional, and national causes of under-5 mortality in 2000-15: an updated systematic analysis with implications for the Sustainable Development

For peer review only - http://bmjopen.bmj.com/site/about/guidelines.xhtml

Goals. Lancet. 2016 Dec 17;388(10063):3027–35.

4. Imdad A, Bhutta ZA. Nutritional management of the low birth weight/preterm infant in community settings: a perspective from the developing world. J Pediatr. 2013 Mar;162(3 Suppl):S107-14.

5. Blencowe H, Cousens S, Oestergaard MZ, Chou D, Moller A-B, Narwal R, et al. National, regional, and worldwide estimates of preterm birth rates in the year 2010 with time trends since 1990 for selected countries: a systematic analysis and implications. Lancet. 2012 Jun 9;379(9832):2162–72.

6. Katz J, Lee AC, Kozuki N, Lawn JE, Cousens S, Blencowe H, et al. Mortality risk in preterm and small-for-gestational-age infants in low-income and middle-income countries: a pooled country analysis. Lancet. 2013 Aug 3;382(9890):417–25.

7. Lee ACC, Katz J, Blencowe H, Cousens S, Kozuki N, Vogel JP, et al. National and regional estimates of term and preterm babies born small for gestational age in 138 low-income and middle-income countries in 2010. Lancet Glob Health. 2013 Jul;1(1):e26-36.

8. Mwaniki MK, Atieno M, Lawn JE, Newton CRJC. Long-term neurodevelopmental outcomes after intrauterine and neonatal insults: a systematic review. Lancet. 2012 Feb 4;379(9814):445–52.

9. Lawn JE, Kerber K, Enweronu-Laryea C, Cousens S. 3.6 million neonatal deaths--what is progressing and what is not? Semin Perinatol. 2010 Dec;34(6):371–86.

10. Pawar SJ, Oleti T, Bharathi S, Tipparaju S, Mustafa E. Growth and Neurodevelopmental Outcome in Preterm LBW Infants with Sepsis in India: A Prospective Cohort. Int J Pediatr. 2018 Feb 21;2018:5735632.

11. Upadhyay RP, Martines JC, Taneja S, Mazumder S, Bahl R, Bhandari N, et al. Risk of postneonatal mortality, hospitalisation and suboptimal breast feeding practices in low birthweight infants from rural Haryana, India: findings from a secondary data analysis. BMJ Open. 2018 Jun 22;8(6):e020384.

12. Rana R, McGrath M, Gupta P, Thakur E, Kerac M. Feeding Interventions for Infants with Growth Failure in the First Six Months of Life: A Systematic Review. Nutrients. 2020 Jul 9;12(7).

13. Christian P, Lee SE, Donahue Angel M, Adair LS, Arifeen SE, Ashorn P, et al. Risk of childhood undernutrition related to small-for-gestational age and preterm birth in low- and middle-income countries. Int J Epidemiol. 2013 Oct;42(5):1340–55.

14. Lawn JE, Kerber K, Enweronu-Laryea C, Massee Bateman O. Newborn survival in low resource settings--are we delivering? BJOG. 2009 Oct;116 Suppl 1:49–59.

15. Wardlaw TM, World Health Organization, UNICEF. Low birthweight: Country, regional and global estimates. Geneva: WHO; 2004.

16. Blanc AK, Wardlaw T. Monitoring low birth weight: an evaluation of international estimates and an updated estimation procedure. Bull World Health Organ. 2005 Mar 16;83(3):178–85.

17. Gisore P, Shipala E, Otieno K, Rono B, Marete I, Tenge C, et al. Community based weighing of newborns and use of mobile phones by village elders in rural settings in Kenya: a decentralised approach to health care provision. BMC Pregnancy Childbirth. 2012 Mar 19;12:15.

18. North K, Marx-Delaney M, Bose C, Lee AC, Vesel L, Adair L, et al. Effect of Milk Type and Fortification on Growth of Low-Birthweight Infants: An Umbrella Review of Systematic Reviews and Meta-Analyses. Maternal & Child Nutrition, under review. 2020;

19. Guidelines on Optimal Feeding of Low Birth-Weight Infants in Low- and Middle-Income Countries. Geneva: World Health Organization; 2011.

For peer review only - http://bmjopen.bmj.com/site/about/guidelines.xhtml

20. Brown S, McSharry P. Improving accuracy and usability of growth charts: case study in Rwanda. BMJ Open. 2016 Jan 27;6(1):e009046.

21. JustACTIONS. URGENT CALL FOR AN INVESTIGATION INTO THE FEEDING OF SICK & VULNERABLE NEWBORNS | by JustACTIONS | Medium [Internet]. 2017 [cited 2020 Oct 28]. Available from: https://medium.com/@JustACTIONS/urgent-call-for-an-investigation-into-the-feeding-of-sick-vulnerable-newborns-3f4ddb49c494

22. WHO. Survive and thrive: transforming care for every small and sick newborn. Geneva, Switzerland: World Health Organization; 2018.

23. Blencowe H, Cousens S, Jassir FB, Say L, Chou D, Mathers C, et al. National, regional, and worldwide estimates of stillbirth rates in 2015, with trends from 2000: a systematic analysis. Lancet Glob Health. 2016 Feb;4(2):e98–108.

24. IIPS. National Family Health Survey 2015-2016. Mumbai: International Institute for Population Sciences; 2017.

25. Countdown Country Profiles [Internet]. Countdown to 2030. [cited 2020 Oct 30]. Available from: https://profiles.countdown2030.org/#/ds/MWI

26. IGME. IGME UN Inter-agency Group for Child Mortality Estimation [Internet]. 2020 [cited 2020 Dec 7]. Available from: https://childmortality.org/

27. Sarnat HB, Sarnat MS. Neonatal encephalopathy following fetal distress. A clinical and electroencephalographic study. Arch Neurol. 1976 Oct;33(10):696–705.

28. Roland EH, Hill A. Clinical aspects of perinatal hypoxic-ischemic brain injury. Semin Pediatr Neurol. 1995 Mar 1;2(1):57–71.

29. Olsen EM, Petersen J, Skovgaard AM, Weile B, Jørgensen T, Wright CM. Failure to thrive: the prevalence and concurrence of anthropometric criteria in a general infant population. Arch Dis Child. 2007 Feb;92(2):109–14.

30. Villar J, Giuliani F, Bhutta ZA, Bertino E, Ohuma EO, Ismail LC, et al. Postnatal growth standards for preterm infants: the Preterm Postnatal Follow-up Study of the INTERGROWTH-21(st) Project. Lancet Glob Health. 2015 Nov;3(11):e681-91.

31. Villar J, Cheikh Ismail L, Victora CG, Ohuma EO, Bertino E, Altman DG, et al. International standards for newborn weight, length, and head circumference by gestational age and sex: the Newborn Cross-Sectional Study of the INTERGROWTH-21st Project. Lancet. 2014 Sep 6;384(9946):857–68.

32. de Onis M, Garza C, Victora CG, Onyango AW, Frongillo EA, Martines J. The WHO Multicentre Growth Reference Study: planning, study design, and methodology. Food Nutr Bull. 2004 Mar;25(1 Suppl):S15-26.

33. Kimani-Murage EW, Wanjohi MN, Kamande EW, Macharia TN, Mwaniki E, Zerfu T, et al. Perceptions on donated human milk and human milk banking in Nairobi, Kenya. Matern Child Nutr. 2019 Jul 26;15(4):e12842.

34. Mansen K, Nguyen TT, Nguyen NQ, Do CT, Tran HT, Nguyen NT, et al. Strengthening newborn nutrition through establishment of the first human milk bank in vietnam. J Hum Lact. 2020 Aug 24;890334420948448.

35. Mondkar J, Chugh Sachdeva R, Shanbhag S, Khan A, Manuhar Sinha M, Dasgupta R, et al. Understanding barriers and facilitators for human milk banking among service providers, mothers, and

For peer review only - http://bmjopen.bmj.com/site/about/guidelines.xhtml

influencers of preterm and sick neonates admitted at two health facilities in a metropolitan city in india. Breastfeed Med. 2018 Oct 31;13(10):694–701.

36. PATH. Strengthening Human Milk Banking: A Resource Toolkit for Establishing and Integrating Human Milk Bank Programs--An Assessment Tool for Determining Facility Readiness. Seattle, Washington, USA: PATH; 2019.

37. Clopton N. The dubowitz assessment of gestational age. Phys Occup Ther Pediatr. 1983;3(3):75–86.

38. Dubowitz LM, Dubowitz V, Goldberg C. Clinical assessment of gestational age in the newborn infant. J Pediatr. 1970 Jul;77(1):1–10.

39. Topp CW, Østergaard SD, Søndergaard S, Bech P. The WHO-5 Well-Being Index: a systematic review of the literature. Psychother Psychosom. 2015 Mar 28;84(3):167–76.

40. World Health Organization. Indicators forassessing infant and young childfeeding practices. Part 1 Definitions. [Internet]. World Health Organization; 2008 [cited 2020 Dec 18]. Available from: https://apps.who.int/iris/bitstream/handle/10665/43895/9789241596664_eng.pdf?sequence=1

41. Jensen D, Wallace S, Kelsay P. LATCH: a breastfeeding charting system and documentation tool. J Obstet Gynecol Neonatal Nurs. 1994 Jan;23(1):27–32.

42. Hedberg Nyqvist K, Ewald U. Infant and maternal factors in the development of breastfeeding behaviour and breastfeeding outcome in preterm infants. Acta Paediatr. 1999 Nov;88(11):1194–203.

43. Pados BF, Estrem HH, Thoyre SM, Park J, McComish C. The neonatal eating assessment tool: development and content validation. Neonatal Netw. 2017 Nov 1;36(6):359–67.

44. Manea L, Gilbody S, Hewitt C, North A, Plummer F, Richardson R, et al. Identifying depression with the PHQ-2: A diagnostic meta-analysis. J Affect Disord. 2016 Oct;203:382–95.

45. McCoy DC, Sudfeld CR, Bellinger DC, Muhihi A, Ashery G, Weary TE, et al. Development and validation of an early childhood development scale for use in low-resourced settings. Popul Health Metr. 2017 Feb 9;15(1):3.

46. StataCorp LLC. StataCorp: Stata Statistical Software. College Station, TX: StataCorp LLC.; 2019.

47. SAS Institute. SAS/IML Software . Cary, NC: SAS Institute; 1990.

48. Gale RC, Wu J, Erhardt T, Bounthavong M, Reardon CM, Damschroder LJ, et al. Comparison of rapid vs in-depth qualitative analytic methods from a process evaluation of academic detailing in the Veterans Health Administration. Implement Sci. 2019 Feb 1;14(1):11.

49. Taylor B, Henshall C, Kenyon S, Litchfield I, Greenfield S. Can rapid approaches to qualitative analysis deliver timely, valid findings to clinical leaders? A mixed methods study comparing rapid and thematic analysis. BMJ Open. 2018 Oct 8;8(10):e019993.

50. Gale NK, Heath G, Cameron E, Rashid S, Redwood S. Using the framework method for the analysis of qualitative data in multi-disciplinary health research. BMC Med Res Methodol. 2013 Sep 18;13:117.

51. SocioCultural Research Consultants, LLC. Dedoose . Los Angeles, CA: SocioCultural Research Consultants, LLC; 2018.

For peer review only

SPIRIT
STANDARD PROTOCOL ITEMS: RECOMMENDATIONS FOR INTERVENTIONAL TRIALS

SPIRIT 2013 Checklist: Recommended items to address in a clinical trial protocol and related documents*

Section/item	Item No	Description	Addressed on page number
**Administrative information**			
Title	1	Descriptive title identifying the study design, population, interventions, and, if applicable, trial acronym	________1______
Trial registration	2a	Trial identifier and registry name. If not yet registered, name of intended registry	________2_____
	2b	All items from the World Health Organization Trial Registration Data Set	________N/A____
Protocol version	3	Date and version identifier	_______ N/A_____
Funding	4	Sources and types of financial, material, and other support	_______ 14_____
Roles and responsibilities	5a	Names, affiliations, and roles of protocol contributors	________1, 14____
	5b	Name and contact information for the trial sponsor	________14______
	5c	Role of study sponsor and funders, if any, in study design; collection, management, analysis, and interpretation of data; writing of the report; and the decision to submit the report for publication, including whether they will have ultimate authority over any of these activities	________14______
	5d	Composition, roles, and responsibilities of the coordinating centre, steering committee, endpoint adjudication committee, data management team, and other individuals or groups overseeing the trial, if applicable (see Item 21a for data monitoring committee)	________14______

For peer review only

**Introduction**			
Background and rationale	6a	Description of research question and justification for undertaking the trial, including summary of relevant studies (published and unpublished) examining benefits and harms for each intervention	_____3________
	6b	Explanation for choice of comparators	______3_______
Objectives	7	Specific objectives or hypotheses	______4_______
Trial design	8	Description of trial design including type of trial (eg, parallel group, crossover, factorial, single group), allocation ratio, and framework (eg, superiority, equivalence, noninferiority, exploratory)	______N/A (4)__
**Methods: Participants, interventions, and outcomes**			
Study setting	9	Description of study settings (eg, community clinic, academic hospital) and list of countries where data will be collected. Reference to where list of study sites can be obtained	_____5________
Eligibility criteria	10	Inclusion and exclusion criteria for participants. If applicable, eligibility criteria for study centres and individuals who will perform the interventions (eg, surgeons, psychotherapists)	______6-7_____
Interventions	11a	Interventions for each group with sufficient detail to allow replication, including how and when they will be administered	____N/A________
	11b	Criteria for discontinuing or modifying allocated interventions for a given trial participant (eg, drug dose change in response to harms, participant request, or improving/worsening disease)	_____N/A_______
	11c	Strategies to improve adherence to intervention protocols, and any procedures for monitoring adherence (eg, drug tablet return, laboratory tests)	______N/A______
	11d	Relevant concomitant care and interventions that are permitted or prohibited during the trial	______N/A______
Outcomes	12	Primary, secondary, and other outcomes, including the specific measurement variable (eg, systolic blood pressure), analysis metric (eg, change from baseline, final value, time to event), method of aggregation (eg, median, proportion), and time point for each outcome. Explanation of the clinical relevance of chosen efficacy and harm outcomes is strongly recommended	_______8______
Participant timeline	13	Time schedule of enrolment, interventions (including any run-ins and washouts), assessments, and visits for participants. A schematic diagram is highly recommended (see Figure)	___8, 10-11_____

For peer review only - http://bmjopen.bmj.com/site/about/guidelines.xhtml

Sample size	14	Estimated number of participants needed to achieve study objectives and how it was determined, including clinical and statistical assumptions supporting any sample size calculations	__4-5, 11______
Recruitment	15	Strategies for achieving adequate participant enrolment to reach target sample size	____11_________

Methods: Assignment of interventions (for controlled trials)

Allocation:

Sequence generation	16a	Method of generating the allocation sequence (eg, computer-generated random numbers), and list of any factors for stratification. To reduce predictability of a random sequence, details of any planned restriction (eg, blocking) should be provided in a separate document that is unavailable to those who enrol participants or assign interventions	____N/A________
Allocation concealment mechanism	16b	Mechanism of implementing the allocation sequence (eg, central telephone; sequentially numbered, opaque, sealed envelopes), describing any steps to conceal the sequence until interventions are assigned	____N/A________
Implementation	16c	Who will generate the allocation sequence, who will enrol participants, and who will assign participants to interventions	______N/A______
Blinding (masking)	17a	Who will be blinded after assignment to interventions (eg, trial participants, care providers, outcome assessors, data analysts), and how	_____N/A_______
	17b	If blinded, circumstances under which unblinding is permissible, and procedure for revealing a participant's allocated intervention during the trial	_____N/A_______

Methods: Data collection, management, and analysis

Data collection methods	18a	Plans for assessment and collection of outcome, baseline, and other trial data, including any related processes to promote data quality (eg, duplicate measurements, training of assessors) and a description of study instruments (eg, questionnaires, laboratory tests) along with their reliability and validity, if known. Reference to where data collection forms can be found, if not in the protocol	____8-12_______
	18b	Plans to promote participant retention and complete follow-up, including list of any outcome data to be collected for participants who discontinue or deviate from intervention protocols	_____8-12______

Data management	19	Plans for data entry, coding, security, and storage, including any related processes to promote data quality (eg, double data entry; range checks for data values). Reference to where details of data management procedures can be found, if not in the protocol	___12-13_______
Statistical methods	20a	Statistical methods for analysing primary and secondary outcomes. Reference to where other details of the statistical analysis plan can be found, if not in the protocol	_____12________
	20b	Methods for any additional analyses (eg, subgroup and adjusted analyses)	____12-13______
	20c	Definition of analysis population relating to protocol non-adherence (eg, as randomised analysis), and any statistical methods to handle missing data (eg, multiple imputation)	____N/A________
**Methods: Monitoring**			
Data monitoring	21a	Composition of data monitoring committee (DMC); summary of its role and reporting structure; statement of whether it is independent from the sponsor and competing interests; and reference to where further details about its charter can be found, if not in the protocol. Alternatively, an explanation of why a DMC is not needed	___N/A_________
	21b	Description of any interim analyses and stopping guidelines, including who will have access to these interim results and make the final decision to terminate the trial	__N/A__________
Harms	22	Plans for collecting, assessing, reporting, and managing solicited and spontaneously reported adverse events and other unintended effects of trial interventions or trial conduct	___9_________
Auditing	23	Frequency and procedures for auditing trial conduct, if any, and whether the process will be independent from investigators and the sponsor	____N/A________
**Ethics and dissemination**			
Research ethics approval	24	Plans for seeking research ethics committee/institutional review board (REC/IRB) approval	____13_________
Protocol amendments	25	Plans for communicating important protocol modifications (eg, changes to eligibility criteria, outcomes, analyses) to relevant parties (eg, investigators, REC/IRBs, trial participants, trial registries, journals, regulators)	___N/A_________

Consent or assent	26a	Who will obtain informed consent or assent from potential trial participants or authorised surrogates, and how (see Item 32)	_____13__________
	26b	Additional consent provisions for collection and use of participant data and biological specimens in ancillary studies, if applicable	_____N/A_________
Confidentiality	27	How personal information about potential and enrolled participants will be collected, shared, and maintained in order to protect confidentiality before, during, and after the trial	______12_________
Declaration of interests	28	Financial and other competing interests for principal investigators for the overall trial and each study site	_______14________
Access to data	29	Statement of who will have access to the final trial dataset, and disclosure of contractual agreements that limit such access for investigators	______13_________
Ancillary and post-trial care	30	Provisions, if any, for ancillary and post-trial care, and for compensation to those who suffer harm from trial participation	______N/A________
Dissemination policy	31a	Plans for investigators and sponsor to communicate trial results to participants, healthcare professionals, the public, and other relevant groups (eg, via publication, reporting in results databases, or other data sharing arrangements), including any publication restrictions	______13_________
	31b	Authorship eligibility guidelines and any intended use of professional writers	_____N/A_________
	31c	Plans, if any, for granting public access to the full protocol, participant-level dataset, and statistical code	_____13__________
**Appendices**			
Informed consent materials	32	Model consent form and other related documentation given to participants and authorised surrogates	_____N/A_________
Biological specimens	33	Plans for collection, laboratory evaluation, and storage of biological specimens for genetic or molecular analysis in the current trial and for future use in ancillary studies, if applicable	_____N/A_________

*It is strongly recommended that this checklist be read in conjunction with the SPIRIT 2013 Explanation & Elaboration for important clarification on the items. Amendments to the protocol should be tracked and dated. The SPIRIT checklist is copyrighted by the SPIRIT Group under the Creative Commons "Attribution-NonCommercial-NoDerivs 3.0 Unported" license.

For peer review only - http://bmjopen.bmj.com/site/about/guidelines.xhtml

BMJ Open

A mixed-methods, descriptive and observational cohort study examining feeding and growth patterns among low birthweight infants in India, Malawi and Tanzania: the LIFE study protocol

Journal:	*BMJ Open*
Manuscript ID	bmjopen-2020-048216.R1
Article Type:	Protocol
Date Submitted by the Author:	01-Jul-2021
Complete List of Authors:	Vesel, Linda; Ariadne Labs at Brigham and Women's Hospital and the Harvard T.H. Chan School of Public Health<br>Spigel, Lauren; Ariadne Labs at Brigham and Women's Hospital and the Harvard T.H. Chan School of Public Health<br>Behera , Jnanindra ; SCB Medical College and Hospital, Department of Paediatrics,<br>Bellad, Roopa; KLE Academy of Higher Education and Research<br>Das, Leena; SCB Medical College and Hospital, Department of Paediatrics<br>Dhaded, Sangappa; KLE Academy of Higher Education and Research<br>Goudar, Shivaprasad S.; KLE Academy of Higher Education and Research<br>Guruprasad , Gowdar; JJM Medical College, Department of Neonatology<br>Misra, Sujata; SCB Medical College and Hospital, Department of Paediatrics<br>Panda, Sanghamitra ; City Hospital , Department of Obstetrics and Gynaecology<br>Shamanur , Latha; SS Institute of Medical Sciences and Research Center, Department of Paediatrics<br>Vernekar , Sunil; KLE Academy of Higher Education and Research<br>Hoffman , Irving ; University of North Carolina at Chapel Hill School of Medicine, Institute for Global Health and Infectious Diseases<br>Mvalo, Tisungane; UNC Project; University of North Carolina at Chapel Hill School of Medicine, Department of Pediatrics<br>Phiri, Melda; UNC Project<br>Saidi, Friday; UNC Project<br>Kisenge, Rodrick; Muhimbili University of Health and Allied Sciences, Department of Pediatrics and Child Health<br>Manji, Karim; Muhimbili University of Health and Allied Sciences, Department of Pediatrics and Child Health<br>Salim, Nahya; Muhimbili University of Health and Allied Sciences, Department of Pediatrics and Child Health<br>Somji, Sarah; Muhimbili University of Health and Allied Sciences, Department of Pediatrics and Child Health<br>Sudfeld, CR; Harvard University T H Chan School of Public Health, Departments of Global Health and Population<br>Adair, Linda; University of North Carolina at Chapel Hill Gillings School of Global Public Health, Department of Nutrition<br>Caruso, Bethany; Emory University School of Public Health, Hubert Department of Global Health

	Duggan, Christopher; Boston Children's Hospital, Center for Nutrition<br>Israel-Ballard, Kiersten; PATH, Maternal, Newborn, Child Health and Nutrition Program<br>Lee, Anne; Brigham and Women's Hospital, Pediatric Newborn Medicine<br>Martin, Stephanie; University of North Carolina at Chapel Hill Gillings School of Global Public Health, Department of Nutrition<br>Mansen , Kimberly; PATH, Maternal, Newborn, Child Health and Nutrition Program<br>North, Krysten; University of North Carolina at Chapel Hill School of Medicine, Department of Pediatrics<br>Young, Melissa; Emory University School of Public Health, Hubert Department of Global Health<br>Benotti, Emily; Ariadne Labs at Brigham and Women's Hospital and the Harvard T.H. Chan School of Public Health<br>Marx Delaney , Megan; Ariadne Labs at Brigham and Women's Hospital and the Harvard T.H. Chan School of Public Health<br>Fishman, Eliza; Ariadne Labs at Brigham and Women's Hospital and the Harvard T.H. Chan School of Public Health<br>Fleming, Katelyn; Ariadne Labs at Brigham and Women's Hospital and the Harvard T.H. Chan School of Public Health<br>Henrich, Natalie ; Ariadne Labs at Brigham and Women's Hospital and the Harvard T.H. Chan School of Public Health<br>Miller, Kate; Ariadne Labs at Brigham and Women's Hospital and the Harvard T.H. Chan School of Public Health<br>Subramanian , Laura ; Ariadne Labs at Brigham and Women's Hospital and the Harvard T.H. Chan School of Public Health<br>Tuller, Danielle; Ariadne Labs at Brigham and Women's Hospital and the Harvard T.H. Chan School of Public Health<br>Semrau, Katherine; Ariadne Labs at Brigham and Women's Hospital and the Harvard T.H. Chan School of Public Health; Harvard Medical School, Department of Medicine
**Primary Subject Heading**:	Global health
Secondary Subject Heading:	Nutrition and metabolism
Keywords:	EPIDEMIOLOGY, NEONATOLOGY, NUTRITION & DIETETICS, PUBLIC HEALTH, QUALITATIVE RESEARCH

SCHOLARONE™
Manuscripts

I, the Submitting Author has the right to grant and does grant on behalf of all authors of the Work (as defined in the below author licence), an exclusive licence and/or a non-exclusive licence for contributions from authors who are: i) UK Crown employees; ii) where BMJ has agreed a CC-BY licence shall apply, and/or iii) in accordance with the terms applicable for US Federal Government officers or employees acting as part of their official duties; on a worldwide, perpetual, irrevocable, royalty-free basis to BMJ Publishing Group Ltd ("BMJ") its licensees and where the relevant Journal is co-owned by BMJ to the co-owners of the Journal, to publish the Work in this journal and any other BMJ products and to exploit all rights, as set out in our licence.

The Submitting Author accepts and understands that any supply made under these terms is made by BMJ to the Submitting Author unless you are acting as an employee on behalf of your employer or a postgraduate student of an affiliated institution which is paying any applicable article publishing charge ("APC") for Open Access articles. Where the Submitting Author wishes to make the Work available on an Open Access basis (and intends to pay the relevant APC), the terms of reuse of such Open Access shall be governed by a Creative Commons licence – details of these licences and which Creative Commons licence will apply to this Work are set out in our licence referred to above.

Other than as permitted in any relevant BMJ Author's Self Archiving Policies, I confirm this Work has not been accepted for publication elsewhere, is not being considered for publication elsewhere and does not duplicate material already published. I confirm all authors consent to publication of this Work and authorise the granting of this licence.

For peer review only

A mixed-methods, descriptive and observational cohort study examining feeding and growth patterns among low birthweight infants in India, Malawi and Tanzania: the LIFE study protocol

Linda Vesel[1], Lauren Spigel[1], Jnanindra Nath Behera[2], Roopa M Bellad[3], Leena Das[2], Sangappa M Dhaded[3], Shivaprasad S Goudar[3], Gowdar Guruprasad[4], Sujata Misra[2], Sanghamitra Panda[5], Latha G Shamanur[6], Sunil S Vernekar[3], Irving F Hoffman[7], Tisungane Mvalo[8,9], Melda Phiri[8], Friday Saidi[8], Rodrick Kisenge[10], Karim Manji[10], Nahya Salim[10], Sarah S Somji[10], Christopher R Sudfeld[11], Linda S Adair[12], Bethany A Caruso[13], Christopher P Duggan[14], Kiersten Israel-Ballard[15], Anne CC Lee[16], Stephanie L Martin[12], Kimberly L Mansen[15], Krysten North[16], Melissa F Young[13], Emily Benotti[1], Megan Marx Delaney[1], Eliza Fishman[1], Katelyn Fleming[1], Natalie Henrich[1], Kate Miller[1], Laura Subramanian[1], Danielle E Tuller[1], Katherine E. A. Semrau[1,17]

[1] Ariadne Labs at Brigham and Women's Hospital and the Harvard T.H. Chan School of Public Health, Boston, Massachusetts, USA
[2] Department of Paediatrics, SCB Medical College, Cuttack, Odisha, India
[3] KLE Academy of Higher Education and Research's Jawaharlal Nehru Medical College, Belgaum, Karnataka, India
[4] Department of Neonatology, JJM Medical College, Davangere, Karnataka, India
[5] Department of Obstetrics and Gynaecology, City Hospital, Cuttack, Odisha, India
[6] Department of Pediatrics, SS Institute of Medical Sciences, Davangere, Karnataka, India
[7] Institute for Global Health and Infectious Diseases, University of North Carolina School of Medicine, University of North Carolina at Chapel Hill, Chapel Hill, North Carolina, USA
[8] University of North Carolina Project Malawi, Lilongwe, Malawi
[9] Department of Pediatrics, School of Medicine, University of North Carolina at Chapel Hill, NC, USA
[10] Department of Pediatrics and Child Health, Muhimbili University of Health and Allied Sciences, Dar es Salaam, Tanzania
[11] Departments of Global Health and Population and Nutrition, Harvard T.H. Chan School of Public Health, Boston, MA, USA
[12] Department of Nutrition, Gillings School of Global Public Health, University of North Carolina at Chapel Hill, Chapel Hill, NC, USA
[13] Hubert Department of Global Health, Emory University Rollins School of Public Health, Atlanta, GA, USA
[14] Center for Nutrition, Boston Children's Hospital, Boston, MA, USA
[15] Maternal, Newborn, Child Health and Nutrition Program, PATH, Seattle, WA, USA
[16] Department of Pediatric Newborn Medicine, Brigham and Women's Hospital, Boston, MA, USA
[17] Department of Medicine, Harvard Medical School. Boston, MA, USA

Corresponding Author: Linda Vesel, 401 Park Drive, 3rd Floor, Boston, MA 02215, USA, lvesel@ariadnelabs.org

Word count (excluding title page, abstract, references, figures and tables): 3905

For peer review only - http://bmjopen.bmj.com/site/about/guidelines.xhtml

ABSTRACT

Introduction

Ending preventable deaths of newborns and children under five will not be possible without evidence-based strategies addressing the health and care of low birthweight (LBW, <2.5kg) infants. The majority of LBW infants are born in low- and middle-income countries (LMICs) and account for more than 60-80% of newborn deaths. Feeding promotion tailored to meet the nutritional needs of LBW infants in LMICs may serve a crucial role in curbing newborn mortality rates and promoting growth. The Low Birthweight Infant Feeding Exploration (LIFE) study aims to establish foundational knowledge regarding optimal feeding options for LBW infants in low-resource settings throughout infancy.

Methods and analysis

LIFE is a formative, multi-site, observational cohort study involving 12 study facilities in India, Malawi and Tanzania and using a convergent parallel, mixed-methods design. We assess feeding patterns, growth indicators, morbidity, mortality, child development, and health system inputs that facilitate or hinder care and survival of LBW infants.

Ethics and dissemination

This study was approved by 11 ethics committees in India, Malawi, Tanzania and the United States. The results will be disseminated through peer-reviewed publications and presentations targeting the global and local research, clinical, program implementation and policy communities.

Registration details

Clinical Trials Registration: NCT04002908 (www.clinicaltrials.gov) and CTRI/2019/02/017475 (Clinical Trial Registry of India - http://ctri.nic.in).

STRENGTHS & LIMITATIONS OF THIS STUDY

- Our convergent parallel, mixed-methods study design will yield detailed and unique data on supply and demand side aspects of infant care, feeding and growth at the facility and community levels throughout the first year of life.
- The LIFE study focuses on low birthweight (LBW) infants with birthweights between 1.5kg and <2.5kg since there is limited data on this birthweight group.
- The multi-site approach enables the comparison of results within and across three countries in Sub-Saharan Africa and South Asia, where the phenotypes of LBW infants may vary.
- The main limitation of this study is that enrollment is facility-based, missing the population of small infants who are born outside the health facility or delay presentation for care within the health system.

KEYWORDS

Low birthweight; nutrition; growth; anthropometrics; newborn; infant; feeding; morbidity; mortality; India; Malawi; Tanzania

For peer review only - http://bmjopen.bmj.com/site/about/guidelines.xhtml

INTRODUCTION

Challenge

Although improvements in child health and survival have been achieved in the past two decades, a significant decline in neonatal mortality is needed to attain Sustainable Development Goal 3.2 by 2030 (1). Focusing attention on the most vulnerable small and sick newborns, namely those born low birthweight (LBW, <2.5 kg), can reduce risk and poor outcomes in the first days, weeks and months of life. LBW, resulting from preterm birth and/or intrauterine growth restriction, accounts for 14.6% of newborn births, but represents 60-80% of newborn deaths (2). This burden is disproportionately concentrated in low- and middle-income countries (LMICs) (2). For at least the last five years, prematurity has been the predominant cause of mortality for children under five years of age (3). The mortality risk among infants born preterm and small-for-gestational age is more than 15 times greater than those born term and appropriate-for-gestational age (2,4–7).

Additionally, LBW infants face increased risks for morbidity, neurodevelopmental impairments, and growth faltering as well as challenges related to breastfeeding, particularly its initiation and exclusivity in early infancy (2,8–12). Inadequate and insufficient feeding contributes to poor growth outcomes, such as stunting, wasting and underweight, for which the risk among LBW infants is already increased (13). Without close monitoring of growth and guidance on optimal feeding, LBW infants may not appropriately catch up to their non-LBW peers.

Design, testing, and implementation of interventions to optimize feeding, growth and development among LBW infants requires a rigorously investigated foundational understanding of current practices, standard of care, outcomes and resource availability in LMICs (14). Limited evidence exists, particularly in low resource settings, on feeding patterns and initiation, the feeding ecosystem, policies for the care of LBW infants, and supply and demand inputs related to various feeding modes (15–17). Generally, studies capturing evidence on nutritional interventions for LBW infants are lacking, hindering the proper management of nutritionally at-risk newborns (18). The World Health Organization (WHO) feeding guidelines for LBW infants were published in 2011 (19); however, most of the recommendations are based on low quality evidence; even so, the majority of available evidence was from high-income countries, and most data were limited to very LBW infants (<1.5kg) even though the majority of LBW infants are born between 1.5kg to <2.5kg (12,20).

Opportunity

Despite these gaps, a number of opportunities in recent years have propelled interest and investment in the care of LBW infants. In 2012, the World Health Assembly highlighted the need to prevent LBW by setting a goal of reducing LBW births by 30% by 2025 as part of a set of six nutrition targets comprising its comprehensive implementation plan on maternal, infant and young child nutrition (2). In 2017, 40 global health organizations issued an urgent call to action for more evidence to address the current state of feeding of sick and vulnerable infants (21). In the past two years, the WHO, the United Nations Children's Fund (UNICEF) and the United States Agency for International Development (USAID) released reports and guidelines focusing on the care of the small and sick newborn (22). With interest and investment galvanized, research is needed to develop evidence-based approaches and translate findings into action to ensure the most vulnerable infants can thrive.

Study Aims

For peer review only - http://bmjopen.bmj.com/site/about/guidelines.xhtml

The Low Birthweight Infant Feeding Exploration (LIFE) study aims to document current feeding practices and growth patterns among LBW infants in LMICs to inform potential feeding interventions. The formative, observational cohort study has four objectives to be assessed among LBW infants using a mixed-methods approach: 1) understand feeding practices and the standard of care underpinning them; 2) explore the beliefs, facilitators and barriers around the feeding of LBW infants; 3) define and document key longitudinal growth and health outcomes up to 12 months of age; and 4) examine the relationships between infant and maternal characteristics, feeding, growth and child development. Before embarking on the formative research, we will conduct extensive desk reviews to better understand the current LBW infant feeding literature and policies in place. The long-term goal of the LIFE study is to inform the design of a future LBW infant feeding and growth trial and, in turn, strengthen the evidence-base for global infant feeding guidelines.

METHODS AND ANALYSIS

Study design

This protocol was developed based on the Strengthening the Reporting of Observational Studies in Epidemiology (STROBE) guidelines, the Consolidated Criteria for Reporting Qualitative Research (COREQ) guidelines, as well as key principles of designing and conducting mixed-methods studies (23–25). LIFE is a formative, multi-site, observational cohort study involving 12 study facilities and using a convergent parallel, mixed-methods design. Quantitative and qualitative data will be collected and analyzed in parallel and then merged at the interpretation phase (25). A mixed methods approach allows us to establish the comprehensive foundational knowledge to design feeding interventions for nutritionally at-risk LBW infants. The purpose of the quantitative observational descriptive component of the LIFE study (including a retrospective chart review, prospective observational cohort, in-facility observational cohort and facility needs assessments) is to evaluate feeding practices and health outcomes among LBW infants and the health system inputs that support their care. The purpose of the qualitative descriptive component (including in-depth interviews (IDIs) and focus group discussions (FGDs)) is to examine the care and feeding of LBW infants from the perspectives of various key stakeholders. Finally, the integration of the quantitative and qualitative findings will allow us to more fully understand the context and reasons for the feeding patterns and health outcomes that we observe. Further details of the study design, objectives and data collection activities can be found in Table 1. Data collection began in August 2019 and is on-going with plans for completion by October 2021.

Table 1. Details of study design, data collection and analysis

	Quantitative Data Strand	Qualitative Data Strand	Merged Quantitative & Qualitative Data Strands
Aim	To document current feeding practices and growth patterns among LBW infants in LMICs in order to inform potential feeding interventions		
Objectives	1. Define and document feeding patterns, and key longitudinal growth and health outcomes from birth to 12 months of age<br>2. Examine the relationships between infant and maternal characteristics, feeding, and growth	Explore the beliefs, facilitators and barriers around the feeding of LBW infants	Understand feeding practices and the beliefs, facilitators, barriers, and standards of care underpinning them in order to better identify infants at-

For peer review only

			risk for poor growth and health outcomes
Research Questions	1. What are the feeding patterns, growth trajectories, and health outcomes among LBW infants from birth to 12 months?<br>2. What are the infant and maternal predictors of poor growth outcomes at 6 and 12 months?<br>3. What are the infant and maternal predictors of non-exclusive breastfeeding in the first 6 months?<br>4. What is the association between the duration of exclusive breastfeeding and growth outcomes at 6 and 12 months?	What do mothers, family members, community members, healthcare providers and other key stakeholders think LBW infants should be fed and why?	What are the current practices, beliefs, facilitators and barriers regarding the feeding of LBW infants in facility and community settings in LMICs?
Study Design	Observational, descriptive quantitative data collection and analysis (formative - no intervention) as part of overall convergent parallel design	Descriptive qualitative data collection and analysis as part of overall convergent parallel design	Convergent parallel design leveraging and merging data from quantitative and qualitative data strands
Data Collection<br><br>(Activity: sample per site)	• Retrospective chart review: 155 mother-infant pairs<br>• Prospective observational cohort: 300 mother-infant pairs<br>• In-facility observational cohort: 35 mother-infant pairs<br>• Facility needs assessments: 1-5 health facilities	• In-depth interviews: 72 participants<br>• Focus group discussions: 12 groups (15-24 participants)	Quantitative and qualitative data collected in parallel as noted in the respective data strands
Data Analysis<br><br>(Activity: analysis methods)	• Retrospective chart review: descriptive statistics (means, medians, standard deviations and frequencies)<br>• Prospective observational cohort: descriptive statistics (means, medians, standard deviations and frequencies) and models exploring relationships between key characteristics, feeding and growth (t-test, chi-squared tests, linear and logistic regression)<br>• In-facility observational cohort: descriptive statistics (means, medians, standard deviations and frequencies)<br>• Facility needs assessment: descriptive statistics (means, medians, standard deviations and frequencies)	• In-depth interviews: thematic framework analysis<br>• Focus group discussions: thematic framework analysis	Quantitative and qualitative data analyzed in parallel

For peer review only - http://bmjopen.bmj.com/site/about/guidelines.xhtml

Interpretation	Merging of findings from quantitative and qualitative data collection strands to compare and contrast findings and provide recommendations on optimal feeding options and timing of growth monitoring in order to prevent infants from becoming nutritionally at-risk

Study setting

The LIFE study is implemented in four sites across three countries: 1) Karnataka State, India and 2) Odisha State, India led by teams at Jawaharlal Nehru Medical College, JJM Medical College, SS Institute of Medical Sciences, City Hospital and Srirama Chandra Bhanja Medical College; 3) Lilongwe, Malawi led by the team at University of North Carolina (UNC) Project Malawi; and (4) Dar-es-Salaam, Tanzania led by the team at Muhimbili University of Health and Allied Sciences. All sites have strong, long-standing relationships with key government stakeholders, placing them in a position to address local and national priorities and advocate for translation of research to practice. Sites were chosen in South Asia and sub-Saharan Africa as these regions represent the greatest burden of LBW as well as the diverse drivers of LBW (2). Investigators will document regional similarities and differences in maternal and infant characteristics, gestational age, birthweight, feeding patterns, and growth. In total, participants are recruited from 12 study facilities (i.e. secondary and tertiary hospitals) chosen based on delivery volume, capacity to care for LBW infants in the first days of life, and willingness of facility leadership to participate. All facilities are located in urban settings with participants residing within a 50 kilometer radius. Additional site details are included in Table 2.

Table 2. Site descriptions

Site	Prevalence of LBW (2,26)	Neonatal mortality rate (27) *Deaths per 1000 live births*	Infant mortality rate (28)	Study facilities *Number and type by site*
India-Karnataka	17.2%	22	28	3 private tertiary hospitals<br>2 public tertiary hospitals
India-Odisha	20.8%			1 public tertiary hospital<br>1 public secondary hospital
Malawi	14.5%	20	31	1 public tertiary hospital<br>1 public secondary hospital
Tanzania	10.5%	20	36	1 public tertiary hospital<br>2 public secondary hospitals

Study population

Quantitative

For the quantitative data collection activities, the study population comprises mother-infant pairs that include newborns with recorded birthweights of 1.5kg to <2.5kg, as well as health facilities (Table 3). All 12 study facilities will participate in the facility needs assessments, including a facility profile and a donor human milk (DHM) bank assessment; further inclusion and exclusion criteria have not been specified.

Qualitative

For peer review only - http://bmjopen.bmj.com/site/about/guidelines.xhtml

For the qualitative data collection activities, the study population includes mothers, family members (e.g. husbands, guardians, mothers' parents, mother's in-laws, grandmothers, sisters, and sisters-in-law), religious and community leaders, traditional healers, clinicians, government officials, supply chain experts, and DHM banking experts meeting specific inclusion criteria (Table 3). Mothers and family members of newborns with recorded birthweights of 1.5kg to <2.5kg are eligible for the IDIs and FGDs. Clinicians who have been at their position for less than six months are not eligible.

Table 3. Inclusion and exclusion criteria for mothers-infant pairs

Data collection activity	Inclusion criteria	Exclusion criteria
Retrospective chart review	• Infants with birthweight of 1.5kg to <2.5kg<br>• Infants discharged before the start of prospective data collection LIFE	• Infants with birthweight <1.5kg<br>• Infants with congenital abnormalities that interfere with feeding (cleft lip or palate; hydrocephalus; gastrointestinal tract anomalies including gastroschisis, omphalocele, or anal atresia; neural tube defects; congenital cardiac defects; suspected trisomy 21; suspected TORCH (Toxoplasmosis, Other agents, Rubella, Cytomegalovirus, and Herpes simplex) infection<br>• Infants with young mothers: <18 years old in Tanzania and India, 16-17 years old and unmarried in Malawi, and all mothers <16 years old in Malawi<br>• Infants who die less than 72 hours from the time of birth<br>• Infants born outside the facility
Longitudinal prospective cohort	• Infants with birthweight between 1.5kg to <2.5kg (as measured at birth, or calculated using algorithm based on time since birth to account for expected postnatal weight loss)<br>• Mother-infant pairs who reside within the catchment area (approximately 50km) of the facility in which they were enrolled	• Infants with birthweight <1.5kg<br>• Infants with congenital abnormalities that interfere with feeding (cleft lip or palate; hydrocephalus; gastrointestinal tract anomalies including gastroschisis, omphalocele, or anal atresia; neural tube defects; congenital cardiac defects; suspected trisomy 21; suspected TORCH infection . Infants with severe neonatal encephalopathy jeopardizing early survival (as determined by modified Sarnat criteria) (29,30)<br>• Infants with young mothers: <18 years old in Tanzania and India, 16-17 years old and unmarried in Malawi, and all mothers <16 years old in Malawi<br>• Infants with mothers who died prior to enrollment<br>• Infants who die less than 72 hours from the time of birth<br>• Infants older than 72 hours at the time of screening<br>• Infants who withdraw less than 72 hours from the time of birth<br>• Infants with a twin or triplet who die prior to the time of screening<br>• Mothers who plan to leave the catchment area within 6 months of study enrollment
In-facility observational cohort	• Infants with birthweight between 1.5kg to <2.5kg	• Infants with birthweight <1.5kg<br>• Infants with congenital abnormalities that interfere with feeding<br>• Infants with young mothers: <18 years old in Tanzania and India, 16-17 years old and unmarried in Malawi, and all mothers <16 years old in Malawi

For peer review only - http://bmjopen.bmj.com/site/about/guidelines.xhtml

		• Infants who die less than 6 hours from the time of birth<br>• Infants with mothers who die less than 6 hours from the time of birth<br>• Infants born outside the facility<br>• Infants older than 6 hours at the time of screening
In-depth interviews and focus group discussions<br>(Birth - 6 months)	• Mothers with infants with birthweight between 1.5kg to <2.5kg aged 0 to 7 months (enrollment in prospective observational cohort not required)<br>• Family members of infants with birthweight between 1.5kg to <2.5kg aged 0 to 7 months who play a role in infant and young child feeding<br>• Religious leaders, community leaders, and traditional healers that are opinion leaders on infant and young child feeding practices in the community<br>• Health care workers currently involved in providing infant and young child feeding<br>• Government officials who support infant and young child feeding programs and policies<br>• Supply chain experts involved in infant and young child feeding supply chain logistics<br>• Human milk bank experts	• Mothers and family members with infants with birthweight ≥2.5kg<br>• Mothers and family members with infants with birthweight <1.5kg<br>• Young mothers: <18 years old in Tanzania and India, 16-17 years old and unmarried in Malawi, and all mothers <16 years old in Malawi<br>• Health care workers who have been in their position for less than 6 months<br>• Government officials who have been at their post for less than 6 months
In-depth interviews<br>(9 - 12 months)	• Mothers with infants with birthweight between 1.5kg to <2.5kg enrolled in the prospective observational cohort and aged 9-12 months	• Mothers with infants who were not enrolled in the prospective cohort or withdrew/died before 9 months of age

Patient and public involvement

As part of the study design, the LIFE team involves clinicians, researchers and community stakeholders familiar with the respective settings and populations. Study tools were piloted with patients and community members to ensure that research questions and indicators are culturally appropriate, acceptable and relevant to the study population.

Study measures

Quantitative

We evaluate key maternal and infant characteristics (e.g., maternal education, maternal age, place of residence, parity, place and type of delivery, gestational age, infant sex and average length of facility stay) to better understand the LBW population in each site and region and to evaluate which characteristics serve as predictors of particular feeding patterns and growth and health outcomes. We examine infant feeding patterns including: early initiation of breastfeeding (within one hour of birth), feeding profiles (exclusive breastmilk, mixed milk feeding or no breastmilk) at each visit week from birth to 12 months, and duration of exclusive breastfeeding (feeding of only breastmilk directly from the breast, expressed or from a donor). We will assess infant growth by measuring weight, length, head circumference and mid-upper arm circumference (MUAC) at 13 timepoints. Anthropometrics will be used to identify stunting (length-for-age z-score <-2SD), wasting (weight-for-length z-score <-2SD) and underweight (weight-for-age z-score <-2SD) at six and 12 months; and

For peer review only - http://bmjopen.bmj.com/site/about/guidelines.xhtml

plot growth trajectories and velocities. Z-scores are derived from the International Fetal and Newborn Growth Consortium for the 21st Century (INTERGROWTH-21st) newborn size at birth and preterm postnatal growth standards (31,32) and the WHO infant growth standards (32) from birth through 12 months. We also examine weeks to birthweight regain, namely lack of regain in the first two weeks (33–35); and identify infants experiencing slow weight gain (<20g/ day) in the second week of life (infants are meant to gain an average of 20g/day in the first month of life and the second week of life is when infants should be gaining back their postnatal weight loss) (36,37). The WHO standards were designed using a cohort of term infants while the INTERGROWTH-21st standards were designed specifically for preterm infants and serve as a complement to the WHO standards in the first six months of life. Additional health outcomes include infant morbidity based on maternal self-report of illnesses and symptoms experienced by the infants in the past week at each study visit; neonatal and infant mortality at any point during follow-up; and child development assessed via the Caregiver Reported Early Childhood Development Instruments (CREDI) at one year of age (38). Maternal study measures including illness, depression (based on the Patient Health Questionnaire-2) and anthropometrics (weight, height and MUAC at birth, 6 weeks, 6 month and 12 months) are collected to evaluate mothers' roles in the feeding and growth of their infants (39). Finally, data on bed capacity, length of stay post-delivery, neonatal intensive care admissions, infrastructure, space, equipment, feeding options, medications, staffing, service availability, and sanitation practices are gathered to assess the health facility inputs available for care provision of LBW infants.

Qualitative

We explore beliefs, barriers, facilitators, risks and benefits of various feeding practices through structured IDIs and FGDs.

Data collection

Quantitative and qualitative data collection occur in parallel through six descriptive study activities (Table 1). For most data collection activities, timing of data collection will be linked with infant age (Figure 1). All data collection is prospective except for the chart reviews. Facility needs assessments are not included in Figure 1 since they will not be linked to infant age; facility profiles are completed at baseline and DHM assessments over the course of the study.

Figure 1. Data collection timeline by activity and infant age

Quantitative

Quantitative data collection is conducted by trained research nurses. The use of multiple data collection activities, combining observations and maternal self-report, helps to reduce bias and allows for triangulation of data. Retrospective patient chart data was collected for infants born in 12 study facilities between July 2018 and October 2019 using a structured survey. Chart reviews were completed before the initiation of prospective observational cohort data collection. The goal for the prospective cohort is to include 300 mother-infant pairs per site during 13 study visits using structured survey tools for infants born between September 2019 and January 2020 (noting that sites will start and end at different times) and completing one-year follow-up in July 2021 (Figure 1). Data are collected in-person at all timepoints, apart from at 32.5 and 45.5 weeks, at which time an abbreviated survey is administered over the phone in Malawi and Tanzania to bridge the three-month time gap between in-person visits in order to reduce loss to follow-up; India sites have previous experience with at least three-month intervals between visits without adverse consequences on follow-up rates. Changes

For peer review only - http://bmjopen.bmj.com/site/about/guidelines.xhtml

made in response to the COVID-19 pandemic included the addition of COVID-19 symptom screening questions, pauses to enrollment, data collection via phone calls where in-person visits were not possible and widening of the 6 month visit window. Table 4 summarizes the assessments and timing of administration for the prospective observational cohort. Focused training was conducted by a pediatrician or research investigator for all assessments listed. Although this is an observational cohort study with no intervention, adverse events are monitored and recorded. The in-facility observational cohort is intended to include 35 mother-infant pairs per site from all participating study facilities via regular feeding observations (15 minutes each) and maternal reports starting within six hours of birth and continuing until facility discharge (first week - every three to four hours; second week - one to two times a day; and third week and thereafter - once daily for unstable infants and every three days for stable infants) (Table 4). Finally, facility needs assessment data are collected via standardized tools capturing key vital statistics; the structural, human resource, equipment and service inputs present for new mothers and newborns; and facility and programmatic readiness for establishing and strengthening DHM banks (33–36). Study team members administer the assessments at each of the study facilities through observations, record reviews and staff consultations; global DHM experts will participate in and provide detailed guidance for the DHM component.

Table 4. Timing of assessments for longitudinal prospective observational cohort

**Assessment or survey tool content**	**Age of infant (weeks)**												
	**0**	**1**	**2**	**4**	**6**	**10**	**14**	**18**	**26**	**32.5**	**39**	**45.5**	**52**
Maternal demographics and pregnancy history*	•												
Infant demographics and delivery information*	•												
Dubowitz examination for gestational age at birth (40,41)	•												
Infant anthropometrics*	•	•	•	•	•	•	•	•	•		•		•
Maternal anthropometrics	•				•				•				•
Maternal and infant health information	•	•	•	•	•	•	•	•	•		•		•
Maternal and infant mortality information*	•	•	•	•	•	•	•	•	•	•	•	•	•
The World Health Organization-5 Well-Being Index (WHO-5) (42)											•		•
Maternal lactation and infant feeding information*	•	•	•	•	•	•	•	•	•	•	•	•	•
Infant and Young Child Feeding (IYCF) Questionnaire for complementary feeding period (43)											•		•

For peer review only - http://bmjopen.bmj.com/site/about/guidelines.xhtml

Latch, Audible Swallowing, Nipple Type, Comfort and Hold (LATCH) breastfeeding assessment* (44)		•	•	•	•	•	•	•	•				
Preterm Infant Breastfeeding Behavior Scale (PIBBS)* (45)		•	•	•	•								
Neonatal Eating Assessment Tool (NeoEAT) (46)		•	•	•	•	•	•	•	•				
Water, sanitation and hygiene information*		•	•	•	•	•	•	•	•		•		•
Patient Health Questionnaire 2 (PHQ-2) on maternal depression (47)		•	•	•	•	•	•	•	•		•		•
Caregiver Reported Early Childhood Development Instrument (CREDI) (38)													•

*Assessments also to be completed for the in-facility observational cohort between birth and facility discharge.

All sample sizes were determined to account for timeline, feasibility and resource constraints. Apart from the prospective observational cohort, we will not aim to make statistical inferences or precise point estimates from these samples, but will use the data descriptively for each infant to construct a narrative of their feeding patterns and health outcomes. The main statistical results from the prospective observational cohort will be point estimates and confidence intervals for certain rates, such as the percent of LBW infants who fail to thrive or whose growth falters, develop problems breastfeeding, or fall ill. The size of the true proportions will determine the precision of the estimate (e.g., with a sample of 300 mother-infant pairs at each site, a true proportion of 10% can be detected with precision of +/- 3.6%). The 95% confidence interval would be 6.4% to 13.6% (Figure 2).

Figure 2. Margin of error in estimation of a single proportion with a sample of 300 at each study site for the prospective observational cohort

To ensure standardization and facilitate high quality data collection, investigators will conduct site-specific workshops to review tools and train research staff on how to prepare for and conduct all key assessments; timing and duration will be tailored to the needs and knowledge of each team. Supervisors perform regular quality checks and refresher training as needed. Standardized anthropometric equipment includes: Seca 334 mobile digital baby scale, 887/876/874 digital flat scale with foot pedal for maternal weight, site-specific height boards for mothers, Seca 417 infant measuring board, Shorr mid-upper arm circumference (MUAC) tapes for infants and mothers (WM-MUAC26), and Shorr 65cm head circumference tapes for infants (SKU: WM-STape). The survey tools are administered in the local language (Hindi, Kannada and Marathi in India-Karnataka; Hindi and Oriya in India-Odisha; Chichewa in Malawi; and Swahili in Tanzania).

Qualitative

IDIs and FGDs with mothers and family members of LBW infants, community members, clinicians, government officials, and supply chain and DHM knowledge experts took place between July 2019 and January 2021. Interviewers include males and females with clinical and/or research backgrounds including specialized training in qualitative data collection methods. They conduct IDIs and FGDs with stakeholders and clinicians in English, and with mothers, family members and community leaders in relevant local languages. Interviewers are not personally known to interviewees and are trained to reduce bias by building rapport with all participants

For peer review only - http://bmjopen.bmj.com/site/about/guidelines.xhtml

and maintaining neutrality and confidentiality. They start each IDI and FGD by sharing the purpose of the study and any potential risks, obtaining consent, and then use structured interview guides with specific probes; guides were piloted prior to data collection. All IDIs and FDGs are audio-recorded with permission from participants and supported with real-time note-taking; transcripts and/or summary notes will not be reviewed by participants. IDIs are scheduled for one hour and FGDs for two. All qualitative data collection takes place in a private location either within a study health facility or in the community setting. Participants are given a small incentive to participate in order to cover the cost of travel and lost wages.

We use purposive sampling for government officials and knowledge experts and convenience sampling based on availability for clinicians, mothers, family members and community leaders. Participants are approached in person or via email and visits are confirmed over the phone. Mothers of LBW infants aged 0-3 months and 4-7 months are sampled from facility patient charts while those aged 9 and 12 months are sampled from the prospective cohort based on their sex and presence or absence of stunting at 6 months of age. Our sampling strategy is designed to reach code saturation rather than meaning saturation (48).

Data management and analysis

Quantitative

Quantitative data are entered and managed in CommCare (49), a secure electronic data capture and management system (retrospective charts review and prospective observational cohorts) and Excel (facility needs assessments). For the prospective observational cohort only, the Tanzania team uses its own electronic data capture system and securely transfers data to be merged with the rest of the data collected in CommCare. All data used for analysis are de-identified and stored securely. Experienced statistical analysts and epidemiologists will use means, medians and standard deviations to describe continuous variables and frequencies and proportions for categorical variables (all activities). For the prospective observational cohort, t-tests, chi-squared tests, and linear and logistic regression models will be used to explore relationships between key maternal and infant characteristics, feeding and growth. Confidence intervals around all measures will be constructed to adjust by site and cluster by mother to account for multiple births. Analyses will be stratified by site, sex and LBW phenotype. Missing data will be accounted for and denominators will be adjusted accordingly. Outliers for z-scores and anthropometrics will be examined and cleaned with the use of existing guidelines and sensitivity analyses, where helpful. Any further analyses will be post-hoc and described separately. Analyses will be conducted using the Stata Statistical Software Package, Version 16 (50) and the SAS statistical software suite (51).

Qualitative

De-identified qualitative data are stored for internal use in SharePoint, a secure web-based document management and storage system. Analysis of the IDIs and FGDs will involve a combination of rapid (framework analysis based on real-time notes supported by audio-recordings, where needed) and in-depth (coding of verbatim translated transcripts) approaches (52–54). Framework analysis and coding of notes into summary tables by interviewers/site-based coders with subsequent coding (using a codebook) in Dedoose by qualitative researchers at Harvard will be used for the first stage of data collection in India and Malawi to enable on-going learning during the data collection process for a large volume of qualitative data (51). For maternal IDIs conducted among those with infants ages 9 months and older in India and Malawi, as well as all of Tanzania's maternal IDIs and FGDs across the full year of infancy, interviewers will transcribe the audio-

For peer review only - http://bmjopen.bmj.com/site/about/guidelines.xhtml

recordings verbatim in English and the qualitative researchers at Harvard will conduct a comprehensive, deductive analysis of the data to identify key messages as well as similarities and differences across sites.

Protocol and registration

The study is registered with clinicaltrials.gov (NCT04002908) and CTRI/2019/02/017475 (Clinical Trial Registry of India - http://ctri.nic.in).

ETHICS & DISSEMINATION

This study was approved by 11 ethics committees in India, Malawi, Tanzania and the United States: 1) India Health Ministry's Screening Committee with Indian Council of Medical Research acting as its secretariat; 2) Directorate of Health and Family Welfare Services, Government of Karnataka which also covers investigators at Women & Children Hospital, Davangere and Chigateri General District Hospital, Davangere; 3) Institutional Ethics Committee of KLE Academy of Higher Education and Research which also covers investigators at JN Medical College, Belagavi and KLES Dr Prabhakar Kore Hospital & Medical Research Center, Belagavi; 4) Institutional Ethics Review Board of SS Institute of Medical Sciences & Research Centre; 5) Institutional Ethics Committee of JJM Medical College which also covers investigators at Bapuji Child Health Institute & Research Centre, Davangere, Women & Children Hospital, Davangere and Chigateri General District Hospital, Davangere; 6) Research and Ethics Committee, Directorate of Health Services, Odisha State which also covers investigators at City Hospital Oriya Bazar, Cuttack; 7) Institutional Ethical Committee, Sriram Chandra Bhanja Medical College, Cuttack; 8) Malawi National Health Sciences Research Committee; 9) Tanzania National Institute of Medical Research; 10) Muhimbili University of Health and Allied Sciences; and 11) United States - Harvard T.H Chan School of Public Health which also covers investigators at Boston Children's Hospital, Brigham and Women's Hospital, Emory University, PATH and University of North Carolina.

Written informed consent is obtained from all IDI, FGD, in-facility observational cohort and prospective observational cohort participants**.** After six months of follow-up, prospective observational cohort participants will be re-consented for an additional six months. Verbal consent will only be obtained in the event of extenuating circumstances where written consent cannot be sought; IRB approval will be needed before proceeding. For the facility needs assessments, verbal consent is sought at the facility leadership level as these activities are considered to be a part of quality improvement. Results from the LIFE study will be disseminated at global- and site-levels through peer-reviewed publications and presentations to key stakeholders during meetings and conferences. Site investigators will also share results informally with participating study facilities. Upon publication of the study results, select data will be made publicly available via Harvard Dataverse (55).

DISCUSSION

The LIFE study will fill critical data gaps in the care and nutrition of LBW infants in LMICs; although limited, most of the existing research in this area is concentrated in high-income countries (18). Overall, evidence is lacking on feeding, care and health of LBW infants born with birthweights of 1.5 to <2.5kg; however, these moderately LBW infants represent the majority (>90%) of global LBW births compared to very LBW infants (<1.5kg) (2,18,56–59). We will aim to establish the foundational knowledge required to design, test and implement the most effective and feasible infant feeding strategies to prevent and address growth faltering among LBW infants in low resource settings. Although human milk is promoted by the WHO, clinicians and policymakers globally as the first choice for feeding infants, exclusive breastfeeding alone may not provide

For peer review only - http://bmjopen.bmj.com/site/about/guidelines.xhtml

adequate nutrition for optimal growth of LBW infants who are known to be at increased risk for stunting, wasting and underweight and may require other feeding types or additional nutrients (13,19,60–63); the new *Standards for improving the quality of care for small and sick newborns in health facilities* (64) highlight the importance of health systems infrastructure, human resources, equipment and supplies and training of healthcare providers to facilitate the care and survival of vulnerable infants. The LIFE study will provide the much-needed evidence to comprehensively understand what LBW infants require, how they differ from infants born ≥2.5 kg, and what rigorous research is critical to strengthen global LBW infant feeding guidelines.

ACKNOWLEDGEMENTS

The authors would like to thank clinical leadership and staff at all study facilities for their buy-in, support and contribution to this work; the mothers and infants for allowing us to have a glimpse into their experiences and sharing key moments of their lives; community members, government officials and subject experts for sharing their perspectives; and all data collectors and study staff for conducting study activities.

AUTHORS' CONTRIBUTIONS

Study conceptualization and design was completed by ACL, BAC, CD, CRS, DET, EB, IH, KEAS, KIB, KLM, KM, KMi, KN, LA, LSp, LSu, LV, MFY, NH, RMB, SMD, SSG and TM. Protocol development and tool design was carried out by ACL, BAC, CRS, DET, EB, EF, GG, IH, JNB, KEAS, KF, KIB, KLM, KM, KMi, KN, LA, LD, LGS, LSp, LSu, LV, MFY, MMD, NH, NS, RK, RMB, SLM, SM, SP, SMD, SSG, SSS, SSV and TM. Tool pilot testing/ modification were conducted by EB, EF, FS, GG, JNB, KF, LD, LGS, LSp, LSu, LV, MP, NH, NS, SM, SP, SSS and SSV. Writing of the manuscript was conducted by LSp and LV. All authors reviewed multiple drafts of the manuscript and approved the final version.

FUNDING STATEMENT

This work was supported by The Bill & Melinda Gates Foundation, grant number OPP1192260. The Bill and Melinda Gates Foundation reviewed the study design and sample-size calculations, but will not be involved in data collection, management, analysis, interpretation, writing of the manuscript, or the decision to submit manuscripts for publication.

COMPETING INTERESTS STATEMENT

All authors completed the ICMJE conflict of interest form and were funded by the Gates Foundation for this work as part of the LIFE study. ACL, BC, CRS, DET, KEAS, KIB, KLM, KM, MMD, RK and SS have received funding from the Gates Foundation for maternal and newborn health work at large. CD reports other from American Society for Nutrition, other from UpToDate, and other from People's Medical Publishing House outside the submitted work. ACL reports grants from the World Health Organization and National Institute of Health NICHD outside the submitted work. All other authors have declared no conflicts of interest.

REFERENCES

1. Goal 3: Ensure healthy lives and promote well-being for all at all ages — SDG Indicators. United Nations - SDG; 2017.

2. Blencowe H, Krasevec J, de Onis M, Black RE, An X, Stevens GA, et al. National, regional, and worldwide estimates of low birthweight in 2015, with trends from 2000: a systematic analysis. Lancet

For peer review only - http://bmjopen.bmj.com/site/about/guidelines.xhtml

Glob Health. 2019 May 15;7(7):e849–60.

3. Liu L, Oza S, Hogan D, Chu Y, Perin J, Zhu J, et al. Global, regional, and national causes of under-5 mortality in 2000-15: an updated systematic analysis with implications for the Sustainable Development Goals. Lancet. 2016 Dec 17;388(10063):3027–35.

4. Imdad A, Bhutta ZA. Nutritional management of the low birth weight/preterm infant in community settings: a perspective from the developing world. J Pediatr. 2013 Mar;162(3 Suppl):S107-14.

5. Blencowe H, Cousens S, Oestergaard MZ, Chou D, Moller A-B, Narwal R, et al. National, regional, and worldwide estimates of preterm birth rates in the year 2010 with time trends since 1990 for selected countries: a systematic analysis and implications. Lancet. 2012 Jun 9;379(9832):2162–72.

6. Katz J, Lee AC, Kozuki N, Lawn JE, Cousens S, Blencowe H, et al. Mortality risk in preterm and small-for-gestational-age infants in low-income and middle-income countries: a pooled country analysis. Lancet. 2013 Aug 3;382(9890):417–25.

7. Lee ACC, Katz J, Blencowe H, Cousens S, Kozuki N, Vogel JP, et al. National and regional estimates of term and preterm babies born small for gestational age in 138 low-income and middle-income countries in 2010. Lancet Glob Health. 2013 Jul;1(1):e26-36.

8. Mwaniki MK, Atieno M, Lawn JE, Newton CRJC. Long-term neurodevelopmental outcomes after intrauterine and neonatal insults: a systematic review. Lancet. 2012 Feb 4;379(9814):445–52.

9. Lawn JE, Kerber K, Enweronu-Laryea C, Cousens S. 3.6 million neonatal deaths--what is progressing and what is not? Semin Perinatol. 2010 Dec;34(6):371–86.

10. Pawar SJ, Oleti T, Bharathi S, Tipparaju S, Mustafa E. Growth and Neurodevelopmental Outcome in Preterm LBW Infants with Sepsis in India: A Prospective Cohort. Int J Pediatr. 2018 Feb 21;2018:5735632.

11. Upadhyay RP, Martines JC, Taneja S, Mazumder S, Bahl R, Bhandari N, et al. Risk of postneonatal mortality, hospitalisation and suboptimal breast feeding practices in low birthweight infants from rural Haryana, India: findings from a secondary data analysis. BMJ Open. 2018 Jun 22;8(6):e020384.

12. Rana R, McGrath M, Gupta P, Thakur E, Kerac M. Feeding Interventions for Infants with Growth Failure in the First Six Months of Life: A Systematic Review. Nutrients. 2020 Jul 9;12(7).

13. Christian P, Lee SE, Donahue Angel M, Adair LS, Arifeen SE, Ashorn P, et al. Risk of childhood undernutrition related to small-for-gestational age and preterm birth in low- and middle-income countries. Int J Epidemiol. 2013 Oct;42(5):1340–55.

14. Lawn JE, Kerber K, Enweronu-Laryea C, Massee Bateman O. Newborn survival in low resource settings--are we delivering? BJOG. 2009 Oct;116 Suppl 1:49–59.

15. Wardlaw TM, World Health Organization, UNICEF. Low birthweight: Country, regional and global estimates. Geneva: WHO; 2004.

16. Blanc AK, Wardlaw T. Monitoring low birth weight: an evaluation of international estimates and an updated estimation procedure. Bull World Health Organ. 2005 Mar 16;83(3):178–85.

17. Gisore P, Shipala E, Otieno K, Rono B, Marete I, Tenge C, et al. Community based weighing of newborns and use of mobile phones by village elders in rural settings in Kenya: a decentralised approach to health care provision. BMC Pregnancy Childbirth. 2012 Mar 19;12:15.

18. North K, Marx-Delaney M, Bose C, Lee AC, Vesel L, Adair L, et al. Effect of Milk Type and Fortification on Growth of Low-Birthweight Infants: An Umbrella Review of Systematic Reviews and Meta-Analyses.

For peer review only - http://bmjopen.bmj.com/site/about/guidelines.xhtml

For peer review only

Matern Child Nutr. 2021 Jul;17(3):e13176.

19. Guidelines on Optimal Feeding of Low Birth-Weight Infants in Low- and Middle-Income Countries. Geneva: World Health Organization; 2011.

20. Brown S, McSharry P. Improving accuracy and usability of growth charts: case study in Rwanda. BMJ Open. 2016 Jan 27;6(1):e009046.

21. URGENT CALL FOR AN INVESTIGATION INTO THE FEEDING OF SICK & VULNERABLE NEWBORNS | by JustACTIONS | Medium [Internet]. [cited 2021 Jun 24]. Available from: https://medium.com/@JustACTIONS/urgent-call-for-an-investigation-into-the-feeding-of-sick-vulnerable-newborns-3f4ddb49c494.

22. WHO. Survive and thrive: transforming care for every small and sick newborn. Geneva, Switzerland: World Health Organization; 2018.

23. Strengthening the reporting of observational studies in epidemiology (STROBE) statement: guidelines for reporting observational studies. BMJ. 2007 Nov 3;335(7626).

24. Tong A, Sainsbury P, Craig J. Consolidated criteria for reporting qualitative research (COREQ): a 32-item checklist for interviews and focus groups. Int J Qual Health Care. 2007 Dec;19(6):349–57.

25. John W. Creswell and Vicki L. Plano Clark. Designing and Conducting Mixed Methods Research. 3rd ed. 2017.

26. IIPS. National Family Health Survey 2015-2016. Mumbai: International Institute for Population Sciences; 2017.

27. Countdown Country Profiles [Internet]. Countdown to 2030. [cited 2020 Oct 30]. Available from: https://profiles.countdown2030.org/#/ds/MWI.

28. IGME. IGME UNInter-agency Group for Child Mortality Estimation [Internet]. 2020 [cited 2020Dec 7]. Available from: https://childmortality.org/.

29. Sarnat HB, Sarnat MS. Neonatal encephalopathy following fetal distress. A clinical and electroencephalographic study. Arch Neurol. 1976 Oct;33(10):696–705.

30. Roland EH, Hill A. Clinical aspects of perinatal hypoxic-ischemic brain injury. Semin Pediatr Neurol. 1995 Mar 1;2(1):57–71.

31. Villar J, Giuliani F, Bhutta ZA, Bertino E, Ohuma EO, Ismail LC, et al. Postnatal growth standards for preterm infants: the Preterm Postnatal Follow-up Study of the INTERGROWTH-21(st) Project. Lancet Glob Health. 2015 Nov;3(11):e681-91.

32. Villar J, Cheikh Ismail L, Victora CG, Ohuma EO, Bertino E, Altman DG, et al. International standards for newborn weight, length, and head circumference by gestational age and sex: the Newborn Cross-Sectional Study of the INTERGROWTH-21st Project. Lancet. 2014 Sep 6;384(9946):857–68.

33. Noel-Weiss J, Courant G, Woodend AK. Physiological weight loss in the breastfed neonate: a systematic review. Open Med. 2008 Oct 28;2(4):e99–110.

34. Macdonald PD, Ross SRM, Grant L, Young D. Neonatal weight loss in breast and formula fed infants. Arch Dis Child Fetal Neonatal Ed. 2003 Nov;88(6):F472-6.

35. DiTomasso D, Cloud M. Systematic Review of Expected Weight Changes After Birth for Full-Term, Breastfed Newborns. J Obstet Gynecol Neonatal Nurs. 2019 Nov;48(6):593–603.

36. Fenton TR, Anderson D, Groh-Wargo S, Hoyos A, Ehrenkranz RA, Senterre T. An Attempt to

For peer review only - http://bmjopen.bmj.com/site/about/guidelines.xhtml

Standardize the Calculation of Growth Velocity of Preterm Infants-Evaluation of Practical Bedside Methods. J Pediatr. 2018 May;196:77–83.

37. Homan GJ. Failure to thrive: A practical guide. Am Fam Physician. 2016 Aug 15;94(4):295–9.

38. McCoy DC, Sudfeld CR, Bellinger DC, Muhihi A, Ashery G, Weary TE, et al. Development and validation of an early childhood development scale for use in low-resourced settings. Popul Health Metr. 2017 Feb 9;15(1):3.

39. Kroenke K, Spitzer RL, Williams JBW. The Patient Health Questionnaire-2: Validity of a two-item depression screener. Med Care. 2003 Nov;41(11):1284–92.

40. Clopton N. The dubowitz assessment of gestational age. Phys Occup Ther Pediatr. 1983;3(3):75–86.

41. Dubowitz LM, Dubowitz V, Goldberg C. Clinical assessment of gestational age in the newborn infant. J Pediatr. 1970 Jul;77(1):1–10.

42. Topp CW, Østergaard SD, Søndergaard S, Bech P. The WHO-5 Well-Being Index: a systematic review of the literature. Psychother Psychosom. 2015 Mar 28;84(3):167–76.

43. World Health Organization. Indicators forassessing infant and young childfeeding practices. Part 1 Definitions. [Internet]. World Health Organization; 2008 [cited 2020 Dec 18]. Available from: https://apps.who.int/iris/bitstream/handle/10665/43895/9789241596664_eng.pdf?sequence=1.

44. Jensen D, Wallace S, Kelsay P. LATCH: a breastfeeding charting system and documentation tool. J Obstet Gynecol Neonatal Nurs. 1994 Jan;23(1):27–32.

45. Hedberg Nyqvist K, Ewald U. Infant and maternal factors in the development of breastfeeding behaviour and breastfeeding outcome in preterm infants. Acta Paediatr. 1999 Nov;88(11):1194–203.

46. Pados BF, Estrem HH, Thoyre SM, Park J, McComish C. The neonatal eating assessment tool: development and content validation. Neonatal Netw. 2017 Nov 1;36(6):359–67.

47. Manea L, Gilbody S, Hewitt C, North A, Plummer F, Richardson R, et al. Identifying depression with the PHQ-2: A diagnostic meta-analysis. J Affect Disord. 2016 Oct;203:382–95.

48. Hennink MM, Kaiser BN, Marconi VC. Code saturation versus meaning saturation: how many interviews are enough? Qual Health Res. 2017 Mar;27(4):591–608.

49. Dimagi. CommCare. Cambridge, MA: Dimagi; 2021.

50. StataCorp LLC. Stata Statistical Software. College Station, TX: StataCorp LLC; 2019.

51. SAS Institute. SAS Software Suite. Cary, NC: SAS Institute; 2020.

52. Gale RC, Wu J, Erhardt T, Bounthavong M, Reardon CM, Damschroder LJ, et al. Comparison of rapid vs in-depth qualitative analytic methods from a process evaluation of academic detailing in the Veterans Health Administration. Implement Sci. 2019 Feb 1;14(1):11.

53. Taylor B, Henshall C, Kenyon S, Litchfield I, Greenfield S. Can rapid approaches to qualitative analysis deliver timely, valid findings to clinical leaders? A mixed methods study comparing rapid and thematic analysis. BMJ Open. 2018 Oct 8;8(10):e019993.

54. Gale NK, Heath G, Cameron E, Rashid S, Redwood S. Using the framework method for the analysis of qualitative data in multi-disciplinary health research. BMC Med Res Methodol. 2013 Sep 18;13:117.

55. Harvard Dataverse [Internet]. [cited 2021 Jun 24]. Available from: https://dataverse.harvard.edu/.

56. World Health Organization, United Nations Children's Fund (UNICEF). Low birthweight : country, regional and global estimates. World Health Organization; 2004.

For peer review only - http://bmjopen.bmj.com/site/about/guidelines.xhtml

57. O'Leary M, Edmond K, Floyd S, Newton S, Thomas G, Thomas SL. A cohort study of low birth weight and health outcomes in the first year of life, Ghana. Bull World Health Organ. 2017 Aug 1;95(8):574–83.

58. Mahumud RA, Sultana M, Sarker AR. Distribution and determinants of low birth weight in developing countries. J Prev Med Public Health. 2017 Jan;50(1):18–28.

59. Marete I, Ekhaguere O, Bann CM, Bucher SL, Nyongesa P, Patel AB, et al. Regional trends in birth weight in low- and middle-income countries 2013-2018. Reprod Health. 2020 Dec 17;17(Suppl 3):176.

60. Rochow N, Raja P, Liu K, Fenton T, Landau-Crangle E, Göttler S, et al. Physiological adjustment to postnatal growth trajectories in healthy preterm infants. Pediatr Res. 2016 Jun;79(6):870–9.

61. Ziegler EE. Meeting the nutritional needs of the low-birth-weight infant. Ann Nutr Metab. 2011 Jun 21;58 Suppl 1:8–18.

62. Hay WW. Strategies for feeding the preterm infant. Neonatology. 2008 Oct 2;94(4):245–54.

63. Lassi ZS, Rind F, Irfan O, Hadi R, Das JK, Bhutta ZA. Impact of Infant and Young Child Feeding (IYCF) Nutrition Interventions on Breastfeeding Practices, Growth and Mortality in Low- and Middle-Income Countries: Systematic Review. Nutrients. 2020 Mar 10;12(3).

64. World Health Organization. Standards for improving the quality of care for small and sick newborns in health facilities. World Health Organization; 2020.

For peer review only

For peer review only - http://bmjopen.bmj.com/site/about/guidelines.xhtml

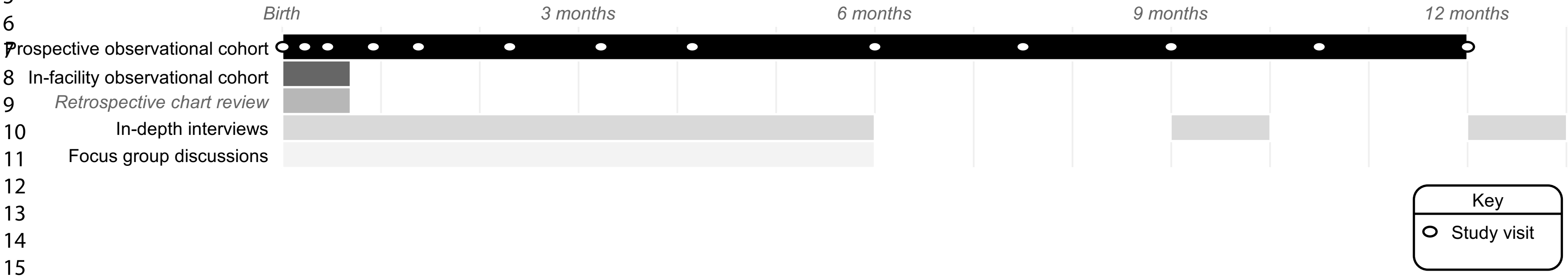

Figure 1. Data collection timeline by activity and infant age

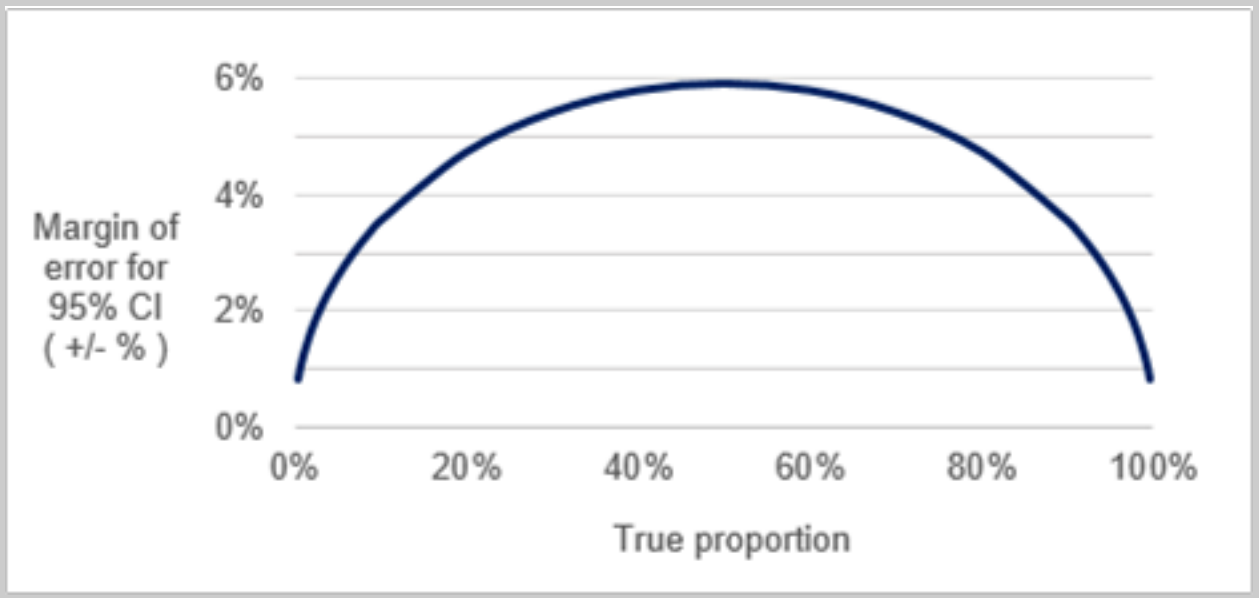

Figure 2. Margin of error in estimation of a single proportion with a sample of 300 at each study site for the prospective observational cohort

STROBE Statement—Checklist of items that should be included in reports of ***cohort studies***

	Item No	Recommendation	Page No
**Title and abstract**	1	(*a*) Indicate the study's design with a commonly used term in the title or the abstract<br>(*b*) Provide in the abstract an informative and balanced summary of what was done and what was found	a) Title: Page 1<br>Abstract: Page 2<br>b) Page 2. Please note that this is a protocol paper so there are no results reported.
**Introduction**			
Background/rationale	2	Explain the scientific background and rationale for the investigation being reported	Page 3
Objectives	3	State specific objectives, including any prespecified hypotheses	Introduction: Pages 3-4<br>Table 1: Pages 4-5
**Methods**			
Study design	4	Present key elements of study design early in the paper	Study design: Page 4<br>Table 1: Page 5
Setting	5	Describe the setting, locations, and relevant dates, including periods of recruitment, exposure, follow-up, and data collection	Study setting: Page 6<br>Table 2: Page 6<br>Data collection: Pages 9-12<br>Table 4: Pages 10-11
Participants	6	(*a*) Give the eligibility criteria, and the sources and methods of selection of participants. Describe methods of follow-up<br>(*b*) For matched studies, give matching criteria and number of exposed and unexposed	a) Study population: Pages 6-8<br>Table 3: Pages 7-8<br>Data collection: Pages 9-12<br>b) Not applicable; this is not a matched study
Variables	7	Clearly define all outcomes, exposures, predictors, potential confounders, and effect modifiers. Give diagnostic criteria, if applicable	Study measures: Pages 8-9<br>Data management and analysis: Pages 12-13
Data sources/ measurement	8*	For each variable of interest, give sources of data and details of methods of assessment (measurement). Describe comparability of assessment methods if there is more than one group	Study measures: Page 8-9<br>Data collection: Pages 9-12
Bias	9	Describe any efforts to address potential sources of bias	Data collection: Pages 9 and 11
Study size	10	Explain how the study size was arrived at	Data collection: Page 11<br>Figure 2: Page 11
Quantitative variables	11	Explain how quantitative variables were handled in the analyses. If applicable, describe which groupings were chosen and why	Data management and analysis: Pages 12-13. However, this is a protocol paper so no analyses have been conducted.
Statistical methods	12	(*a*) Describe all statistical methods, including those used to control for confounding<br>(*b*) Describe any methods used to examine subgroups and interactions	a-c) Data management and analysis: Pages 12-13<br>d) Data collection: Page 9<br>e) Data management and analysis: Page 12

For peer review only - http://bmjopen.bmj.com/site/about/guidelines.xhtml

		(*c*) Explain how missing data were addressed<br>(*d*) If applicable, explain how loss to follow-up was addressed<br>(*e*) Describe any sensitivity analyses	
**Results**			Not applicable because this is a protocol
Participants	13*	(a) Report numbers of individuals at each stage of study—eg numbers potentially eligible, examined for eligibility, confirmed eligible, included in the study, completing follow-up, and analysed<br>(b) Give reasons for non-participation at each stage<br>(c) Consider use of a flow diagram	Not applicable - protocol
Descriptive data	14*	(a) Give characteristics of study participants (eg demographic, clinical, social) and information on exposures and potential confounders<br>(b) Indicate number of participants with missing data for each variable of interest<br>(c) Summarise follow-up time (eg, average and total amount)	Not applicable - protocol
Outcome data	15*	Report numbers of outcome events or summary measures over time	Not applicable - protocol

For peer review only

Main results	16	(*a*) Give unadjusted estimates and, if applicable, confounder-adjusted estimates and their precision (eg, 95% confidence interval). Make clear which confounders were adjusted for and why they were included<br>(*b*) Report category boundaries when continuous variables were categorized<br>(*c*) If relevant, consider translating estimates of relative risk into absolute risk for a meaningful time period	Not applicable - protocol
Other analyses	17	Report other analyses done—eg analyses of subgroups and interactions, and sensitivity analyses	Not applicable - protocol
**Discussion**			
Key results	18	Summarise key results with reference to study objectives	Not applicable – protocol. We have summarized the utility and anticipated outputs in the discussion on page 13.
Limitations	19	Discuss limitations of the study, taking into account sources of potential bias or imprecision. Discuss both direction and magnitude of any potential bias	Strengths & limitations of this study
Interpretation	20	Give a cautious overall interpretation of results considering objectives, limitations, multiplicity of analyses, results from similar studies, and other relevant evidence	Not applicable - protocol
Generalisability	21	Discuss the generalisability (external validity) of the study results	Strengths & limitations of this study
**Other information**			
Funding	22	Give the source of funding and the role of the funders for the present study and, if applicable, for the original study on which the present article is based	Funding statement: Page 14

*Give information separately for exposed and unexposed groups.

Note: An Explanation and Elaboration article discusses each checklist item and gives methodological background and published examples of transparent reporting. The STROBE checklist is best used in conjunction with this article (freely available on the Web sites of PLoS Medicine at http://www.plosmedicine.org/, Annals of Internal Medicine at http://www.annals.org/, and Epidemiology at http://www.epidem.com/). Information on the STROBE Initiative is available at http://www.strobe-statement.org.

For peer review only

For peer review only - http://bmjopen.bmj.com/site/about/guidelines.xhtml

Consolidated criteria for reporting qualitative studies (COREQ): 32-item checklist

Developed from:
Tong A, Sainsbury P, Craig J. Consolidated criteria for reporting qualitative research (COREQ): a 32-item checklist for interviews and focus groups. *International Journal for Quality in Health Care*. 2007. Volume 19, Number 6: pp. 349 – 357

YOU MUST PROVIDE A RESPONSE FOR ALL ITEMS. ENTER N/A IF NOT APPLICABLE

No. Item	Guide questions/description	Reported on Page #
**Domain 1: Research team and reflexivity**		
Personal Characteristics		
1. Interviewer/facilitator	Which author/s conducted the interview or focus group?	N/A – The authors did not conduct the interviews or focus groups
2. Credentials	What were the researcher's credentials? E.g. PhD, MD	Page 11
3. Occupation	What was their occupation at the time of the study?	Page 11
4. Gender	Was the researcher male or female?	Page 11
5. Experience and training	What experience or training did the researcher have?	Page 11
Relationship with participants		
6. Relationship established	Was a relationship established prior to study commencement?	Page 11-12
7. Participant knowledge of the interviewer	What did the participants know about the researcher? e.g. personal goals, reasons for doing the research	Page 11-12
8. Interviewer characteristics	What characteristics were reported about the interviewer/facilitator? e.g. Bias, assumptions, reasons and interests in the research topic	N/A
**Domain 2: study design**		
Theoretical framework		
9. Methodological orientation and theory	What methodological orientation was stated to underpin the study? e.g. grounded theory, discourse analysis, ethnography, phenomenology, content analysis	Pages 4-5
Participant selection		
10. Sampling	How were participants selected? e.g. purposive, convenience, consecutive, snowball	Pages 6-8 & 11-12
11. Method of approach	How were participants approached? e.g.	Page 12

For peer review only

	face-to-face, telephone, mail, email	
12. Sample size	How many participants were in the study?	Page 5
13. Non-participation	How many people refused to participate or dropped out? Reasons?	N/A
Setting		
14. Setting of data collection	Where was the data collected? e.g. home, clinic, workplace	Page 12
15. Presence of non-participants	Was anyone else present besides the participants and researchers?	N/A
16. Description of sample	What are the important characteristics of the sample? e.g. demographic data, date	Pages 4-8
Data collection		
17. Interview guide	Were questions, prompts, guides provided by the authors? Was it pilot tested?	Page 11-12
18. Repeat interviews	Were repeat interviews carried out? If yes, how many?	N/A
19. Audio/visual recording	Did the research use audio or visual recording to collect the data?	Page 12
20. Field notes	Were field notes made during and/or after the inter view or focus group?	Page 12
21. Duration	What was the duration of the interviews or focus group?	Page 12
22. Data saturation	Was data saturation discussed?	Page 12
23. Transcripts returned	Were transcripts returned to participants for comment and/or correction?	Page 12
**Domain 3: analysis and findings**		
Data analysis		
24. Number of data coders	How many data coders coded the data?	N/A
25. Description of the coding tree	Did authors provide a description of the coding tree?	N/A
26. Derivation of themes	Were themes identified in advance or derived from the data?	Page 12
27. Software	What software, if applicable, was used to manage the data?	Page 12
28. Participant checking	Did participants provide feedback on the findings?	Page 12
Reporting		
29. Quotations presented	Were participant quotations presented to illustrate the themes/findings? Was each quotation identified? e.g. participant number	N/A – this is a protocol
30. Data and findings consistent	Was there consistency between the data presented and the findings?	N/A – this is a protocol
31. Clarity of major themes	Were major themes clearly presented in the findings?	N/A – this is a protocol
32. Clarity of minor themes	Is there a description of diverse cases or discussion of minor themes?	N/A – this is a protocol

For peer review only

BMJ Open

A mixed-methods, descriptive and observational cohort study examining feeding and growth patterns among low birthweight infants in India, Malawi and Tanzania: the LIFE study protocol

Journal:	*BMJ Open*
Manuscript ID	bmjopen-2020-048216.R2
Article Type:	Protocol
Date Submitted by the Author:	25-Oct-2021
Complete List of Authors:	Vesel, Linda; Ariadne Labs at Brigham and Women's Hospital and the Harvard T.H. Chan School of Public Health<br>Spigel, Lauren; Ariadne Labs at Brigham and Women's Hospital and the Harvard T.H. Chan School of Public Health<br>Behera , Jnanindra ; SCB Medical College and Hospital, Department of Paediatrics,<br>Bellad, Roopa; KLE Academy of Higher Education and Research<br>Das, Leena; SCB Medical College and Hospital, Department of Paediatrics<br>Dhaded, Sangappa; KLE Academy of Higher Education and Research<br>Goudar, Shivaprasad S.; KLE Academy of Higher Education and Research<br>Guruprasad , Gowdar; JJM Medical College, Department of Neonatology<br>Misra, Sujata; SCB Medical College and Hospital, Department of Paediatrics<br>Panda, Sanghamitra ; City Hospital , Department of Obstetrics and Gynaecology<br>Shamanur , Latha; SS Institute of Medical Sciences and Research Center, Department of Paediatrics<br>Vernekar , Sunil; KLE Academy of Higher Education and Research<br>Hoffman , Irving ; University of North Carolina at Chapel Hill School of Medicine, Institute for Global Health and Infectious Diseases<br>Mvalo, Tisungane; UNC Project; University of North Carolina at Chapel Hill School of Medicine, Department of Pediatrics<br>Phiri, Melda; UNC Project<br>Saidi, Friday; UNC Project<br>Kisenge, Rodrick; Muhimbili University of Health and Allied Sciences, Department of Pediatrics and Child Health<br>Manji, Karim; Muhimbili University of Health and Allied Sciences, Department of Pediatrics and Child Health<br>Salim, Nahya; Muhimbili University of Health and Allied Sciences, Department of Pediatrics and Child Health<br>Somji, Sarah; Muhimbili University of Health and Allied Sciences, Department of Pediatrics and Child Health<br>Sudfeld, CR; Harvard University T H Chan School of Public Health, Departments of Global Health and Population<br>Adair, Linda; University of North Carolina at Chapel Hill Gillings School of Global Public Health, Department of Nutrition<br>Caruso, Bethany; Emory University School of Public Health, Hubert Department of Global Health

	Duggan, Christopher; Boston Children's Hospital, Center for Nutrition<br>Israel-Ballard, Kiersten; PATH, Maternal, Newborn, Child Health and Nutrition Program<br>Lee, Anne; Brigham and Women's Hospital, Pediatric Newborn Medicine<br>Martin, Stephanie; University of North Carolina at Chapel Hill Gillings School of Global Public Health, Department of Nutrition<br>Mansen , Kimberly; PATH, Maternal, Newborn, Child Health and Nutrition Program<br>North, Krysten; University of North Carolina at Chapel Hill School of Medicine, Department of Pediatrics<br>Young, Melissa; Emory University School of Public Health, Hubert Department of Global Health<br>Benotti, Emily; Ariadne Labs at Brigham and Women's Hospital and the Harvard T.H. Chan School of Public Health<br>Marx Delaney , Megan; Ariadne Labs at Brigham and Women's Hospital and the Harvard T.H. Chan School of Public Health<br>Fishman, Eliza; Ariadne Labs at Brigham and Women's Hospital and the Harvard T.H. Chan School of Public Health<br>Fleming, Katelyn; Ariadne Labs at Brigham and Women's Hospital and the Harvard T.H. Chan School of Public Health<br>Henrich, Natalie ; Ariadne Labs at Brigham and Women's Hospital and the Harvard T.H. Chan School of Public Health<br>Miller, Kate; Ariadne Labs at Brigham and Women's Hospital and the Harvard T.H. Chan School of Public Health<br>Subramanian , Laura ; Ariadne Labs at Brigham and Women's Hospital and the Harvard T.H. Chan School of Public Health<br>Tuller, Danielle; Ariadne Labs at Brigham and Women's Hospital and the Harvard T.H. Chan School of Public Health<br>Semrau, Katherine; Ariadne Labs at Brigham and Women's Hospital and the Harvard T.H. Chan School of Public Health; Harvard Medical School, Department of Medicine
**Primary Subject Heading**:	Global health
Secondary Subject Heading:	Nutrition and metabolism
Keywords:	EPIDEMIOLOGY, NEONATOLOGY, NUTRITION & DIETETICS, PUBLIC HEALTH, QUALITATIVE RESEARCH

SCHOLARONE™
Manuscripts

I, the Submitting Author has the right to grant and does grant on behalf of all authors of the Work (as defined in the below author licence), an exclusive licence and/or a non-exclusive licence for contributions from authors who are: i) UK Crown employees; ii) where BMJ has agreed a CC-BY licence shall apply, and/or iii) in accordance with the terms applicable for US Federal Government officers or employees acting as part of their official duties; on a worldwide, perpetual, irrevocable, royalty-free basis to BMJ Publishing Group Ltd ("BMJ") its licensees and where the relevant Journal is co-owned by BMJ to the co-owners of the Journal, to publish the Work in this journal and any other BMJ products and to exploit all rights, as set out in our licence.

The Submitting Author accepts and understands that any supply made under these terms is made by BMJ to the Submitting Author unless you are acting as an employee on behalf of your employer or a postgraduate student of an affiliated institution which is paying any applicable article publishing charge ("APC") for Open Access articles. Where the Submitting Author wishes to make the Work available on an Open Access basis (and intends to pay the relevant APC), the terms of reuse of such Open Access shall be governed by a Creative Commons licence – details of these licences and which Creative Commons licence will apply to this Work are set out in our licence referred to above.

Other than as permitted in any relevant BMJ Author's Self Archiving Policies, I confirm this Work has not been accepted for publication elsewhere, is not being considered for publication elsewhere and does not duplicate material already published. I confirm all authors consent to publication of this Work and authorise the granting of this licence.

For peer review only

A mixed-methods, descriptive and observational cohort study examining feeding and growth patterns among low birthweight infants in India, Malawi and Tanzania: the LIFE study protocol

Linda Vesel[1], Lauren Spigel[1], Jnanindra Nath Behera[2], Roopa M Bellad[3], Leena Das[2], Sangappa M Dhaded[3], Shivaprasad S Goudar[3], Gowdar Guruprasad[4], Sujata Misra[2], Sanghamitra Panda[5], Latha G Shamanur[6], Sunil S Vernekar[3], Irving F Hoffman[7], Tisungane Mvalo[8,9], Melda Phiri[8], Friday Saidi[8], Rodrick Kisenge[10], Karim Manji[10], Nahya Salim[10], Sarah S Somji[10], Christopher R Sudfeld[11], Linda S Adair[12], Bethany A Caruso[13], Christopher P Duggan[14], Kiersten Israel-Ballard[15], Anne CC Lee[16], Stephanie L Martin[12], Kimberly L Mansen[15], Krysten North[16], Melissa F Young[13], Emily Benotti[1], Megan Marx Delaney[1], Eliza Fishman[1], Katelyn Fleming[1], Natalie Henrich[1], Kate Miller[1], Laura Subramanian[1], Danielle E Tuller[1], Katherine E. A. Semrau[1,17]

[1] Ariadne Labs at Brigham and Women's Hospital and the Harvard T.H. Chan School of Public Health, Boston, Massachusetts, USA
[2] Department of Paediatrics, SCB Medical College, Cuttack, Odisha, India
[3] KLE Academy of Higher Education and Research's Jawaharlal Nehru Medical College, Belgaum, Karnataka, India
[4] Department of Neonatology, JJM Medical College, Davangere, Karnataka, India
[5] Department of Obstetrics and Gynaecology, City Hospital, Cuttack, Odisha, India
[6] Department of Pediatrics, SS Institute of Medical Sciences, Davangere, Karnataka, India
[7] Institute for Global Health and Infectious Diseases, University of North Carolina School of Medicine, University of North Carolina at Chapel Hill, Chapel Hill, North Carolina, USA
[8] University of North Carolina Project Malawi, Lilongwe, Malawi
[9] Department of Pediatrics, School of Medicine, University of North Carolina at Chapel Hill, NC, USA
[10] Department of Pediatrics and Child Health, Muhimbili University of Health and Allied Sciences, Dar es Salaam, Tanzania
[11] Departments of Global Health and Population and Nutrition, Harvard T.H. Chan School of Public Health, Boston, MA, USA
[12] Department of Nutrition, Gillings School of Global Public Health, University of North Carolina at Chapel Hill, Chapel Hill, NC, USA
[13] Hubert Department of Global Health, Emory University Rollins School of Public Health, Atlanta, GA, USA
[14] Center for Nutrition, Boston Children's Hospital, Boston, MA, USA
[15] Maternal, Newborn, Child Health and Nutrition Program, PATH, Seattle, WA, USA
[16] Department of Pediatric Newborn Medicine, Brigham and Women's Hospital, Boston, MA, USA
[17] Department of Medicine, Harvard Medical School. Boston, MA, USA

Corresponding Author: Linda Vesel, 401 Park Drive, 3rd Floor, Boston, MA 02215, USA, lvesel@ariadnelabs.org

Word count (excluding title page, abstract, references, figures and tables): 3993

For peer review only - http://bmjopen.bmj.com/site/about/guidelines.xhtml

ABSTRACT

Introduction

Ending preventable deaths of newborns and children under five will not be possible without evidence-based strategies addressing the health and care of low birthweight (LBW, <2.5kg) infants. The majority of LBW infants are born in low- and middle-income countries (LMICs) and account for more than 60-80% of newborn deaths. Feeding promotion tailored to meet the nutritional needs of LBW infants in LMICs may serve a crucial role in curbing newborn mortality rates and promoting growth. The Low Birthweight Infant Feeding Exploration (LIFE) study aims to establish foundational knowledge regarding optimal feeding options for LBW infants in low-resource settings throughout infancy.

Methods and analysis

LIFE is a formative, multi-site, observational cohort study involving 12 study facilities in India, Malawi and Tanzania and using a convergent parallel, mixed-methods design. We assess feeding patterns, growth indicators, morbidity, mortality, child development, and health system inputs that facilitate or hinder care and survival of LBW infants.

Ethics and dissemination

This study was approved by 11 ethics committees in India, Malawi, Tanzania and the United States. The results will be disseminated through peer-reviewed publications and presentations targeting the global and local research, clinical, program implementation and policy communities.

Registration details

Clinical Trials Registration: NCT04002908 (www.clinicaltrials.gov) and CTRI/2019/02/017475 (Clinical Trial Registry of India - http://ctri.nic.in).

STRENGTHS & LIMITATIONS OF THIS STUDY

- Our convergent parallel, mixed-methods study design will yield detailed and unique data on supply and demand side aspects of infant care, feeding and growth at the facility and community levels throughout the first year of life.
- The LIFE study focuses on low birthweight (LBW) infants with birthweights between 1.5kg and <2.5kg since there is limited data on this birthweight group.
- The multi-site approach enables the comparison of results within and across three countries in Sub-Saharan Africa and South Asia, where the phenotypes of LBW infants may vary.
- The main limitation of this study is that enrollment is facility-based, missing the population of small infants who are born outside the health facility or delay presentation for care within the health system.

KEYWORDS

Low birthweight; nutrition; growth; anthropometrics; newborn; infant; feeding; morbidity; mortality; India; Malawi; Tanzania

For peer review only - http://bmjopen.bmj.com/site/about/guidelines.xhtml

INTRODUCTION

Challenge

Although improvements in child health and survival have been achieved in the past two decades, a significant decline in neonatal mortality is needed to attain Sustainable Development Goal 3.2 by 2030 (1). Focusing attention on the most vulnerable small and sick newborns, namely those born low birthweight (LBW, <2.5 kg), can reduce risk and poor outcomes in the first days, weeks and months of life. LBW, resulting from preterm birth and/or intrauterine growth restriction, accounts for 14.6% of newborn births, but represents 60-80% of newborn deaths (2). This burden is disproportionately concentrated in low- and middle-income countries (LMICs) (2). For at least the last five years, prematurity has been the predominant cause of mortality for children under five years of age (3). The mortality risk among infants born preterm and small-for-gestational age is more than 15 times greater than those born term and appropriate-for-gestational age (2,4–7).

Additionally, LBW infants face increased risks for morbidity, neurodevelopmental impairments, and growth faltering as well as challenges related to breastfeeding, particularly its initiation and exclusivity in early infancy (2,8–12). Inadequate and insufficient feeding contributes to poor growth outcomes, such as stunting, wasting and underweight, for which the risk among LBW infants is already increased (13). Without close monitoring of growth and guidance on optimal feeding, LBW infants may not appropriately catch up to their non-LBW peers.

Design, testing, and implementation of interventions to optimize feeding, growth and development among LBW infants requires a rigorously investigated foundational understanding of current practices, standard of care, outcomes and resource availability in LMICs (14). Limited evidence exists, particularly in low resource settings, on feeding patterns and initiation, the feeding ecosystem, policies for the care of LBW infants, and supply and demand inputs related to various feeding modes (15–17). Generally, studies capturing evidence on optimal milk type and content for moderately LBW infants (1.5kg to <2.5kg) in low-resource settings are lacking, hindering the proper management of nutritionally at-risk newborns (18). The World Health Organization (WHO) feeding guidelines for LBW infants were published in 2011 (19); however, most of the recommendations are based on low quality evidence; even so, the majority of available evidence was from high-income countries, and most data were limited to very LBW infants (<1.5kg) even though the majority of LBW infants are born between 1.5kg to <2.5kg (12,20).

Opportunity

Despite these gaps, a number of opportunities in recent years have propelled interest and investment in the care of LBW infants. In 2012, the World Health Assembly highlighted the need to prevent LBW by setting a goal of reducing LBW births by 30% by 2025 as part of a set of six nutrition targets comprising its comprehensive implementation plan on maternal, infant and young child nutrition (2). In 2017, 40 global health organizations issued an urgent call to action for more evidence to address the current state of feeding of sick and vulnerable infants (21). In the past two years, the WHO, the United Nations Children's Fund (UNICEF) and the United States Agency for International Development (USAID) released reports and guidelines focusing on the care of the small and sick newborn (22). With interest and investment galvanized, research is needed to develop evidence-based approaches and translate findings into action to ensure the most vulnerable infants can thrive.

Study Aims

For peer review only - http://bmjopen.bmj.com/site/about/guidelines.xhtml

The Low Birthweight Infant Feeding Exploration (LIFE) study aims to document current feeding practices and growth patterns among LBW infants in LMICs to inform potential feeding interventions. The formative, observational cohort study has four objectives to be assessed among LBW infants using a mixed-methods approach: 1) understand feeding practices and the standard of care underpinning them; 2) explore the beliefs, facilitators and barriers around the feeding of LBW infants; 3) define and document key longitudinal growth and health outcomes up to 12 months of age; and 4) examine the relationships between infant and maternal characteristics, feeding, growth and child development. Before embarking on the formative research, we will conduct extensive desk reviews to better understand the current LBW infant feeding literature and policies in place. The long-term goal of the LIFE study is to inform the design of a future LBW infant feeding and growth trial and, in turn, strengthen the evidence-base for global infant feeding guidelines.

METHODS AND ANALYSIS

Study design

This protocol was developed based on the Strengthening the Reporting of Observational Studies in Epidemiology (STROBE) guidelines, the Consolidated Criteria for Reporting Qualitative Research (COREQ) guidelines, as well as key principles of designing and conducting mixed-methods studies (23–25). LIFE is a formative, multi-site, observational cohort study involving 12 study facilities and using a convergent parallel, mixed-methods design. Quantitative and qualitative data will be collected and analyzed in parallel and then merged at the interpretation phase (25). A mixed methods approach allows us to establish the comprehensive foundational knowledge to design feeding interventions for nutritionally at-risk LBW infants. The purpose of the quantitative observational descriptive component of the LIFE study (including a retrospective chart review, prospective observational cohort, in-facility observational cohort and facility needs assessments) is to evaluate feeding practices and health outcomes among LBW infants and the health system inputs that support their care. The purpose of the qualitative descriptive component (including in-depth interviews (IDIs) and focus group discussions (FGDs)) is to examine the care and feeding of LBW infants from the perspectives of various key stakeholders. Finally, the integration of the quantitative and qualitative findings will allow us to more fully understand the context and reasons for the feeding patterns and health outcomes that we observe. Further details of the study design, objectives and data collection activities can be found in Table 1. Data collection began in August 2019 and is on-going with plans for completion by October 2021.

Table 1. Details of study design, data collection and analysis

	**Quantitative Data Strand**	**Qualitative Data Strand**	**Merged Quantitative & Qualitative Data Strands**
Aim	To document current feeding practices and growth patterns among LBW infants in LMICs in order to inform potential feeding interventions		
Objectives	1. Define and document feeding patterns, and key longitudinal growth and health outcomes from birth to 12 months of age<br>2. Examine the relationships between infant and maternal characteristics, feeding, and growth	1. Explore the beliefs, facilitators and barriers around the feeding of LBW infants	1. Understand feeding practices and the beliefs, facilitators, barriers, and standards of care underpinning them in order to better identify infants at-risk for poor growth and health outcomes

For peer review only - http://bmjopen.bmj.com/site/about/guidelines.xhtml

Research Questions	1. What are the feeding patterns, growth trajectories, and health outcomes among LBW infants from birth to 12 months?<br>2. What are the infant and maternal predictors of poor growth outcomes at 6 and 12 months?<br>3. What are the infant and maternal predictors of non-exclusive breastfeeding in the first 6 months?<br>4. What is the association between the duration of exclusive breastfeeding and growth outcomes at 6 and 12 months?	1. What do mothers, family members, community members, healthcare providers and other key stakeholders think LBW infants should be fed and why?	1. What are the current practices, beliefs, facilitators and barriers regarding the feeding of LBW infants in facility and community settings in LMICs?
Study Design	Observational, descriptive quantitative data collection and analysis (formative - no intervention) as part of overall convergent parallel design	Descriptive qualitative data collection and analysis as part of overall convergent parallel design	Convergent parallel design leveraging and merging data from quantitative and qualitative data strands
Data Collection<br><br>(Activity: sample per site)	• Retrospective chart review: 155 mother-infant pairs<br>• Prospective observational cohort: 300 mother-infant pairs<br>• In-facility observational cohort: 35 mother-infant pairs<br>• Facility needs assessments: 1-5 health facilities	• In-depth interviews: 72 participants<br>• Focus group discussions: 12 groups (15-24 participants)	• Quantitative and qualitative data collected in parallel as noted in the respective data strands
Data Analysis<br><br>(Activity: analysis methods)	• Retrospective chart review: descriptive statistics (means, medians, standard deviations and frequencies)<br>• Prospective observational cohort: descriptive statistics (means, medians, standard deviations and frequencies) and models exploring relationships between key characteristics, feeding and growth (t-test, chi-squared tests, linear and logistic regression)<br>• In-facility observational cohort: descriptive statistics (means, medians, standard deviations and frequencies)<br>• Facility needs assessment: descriptive statistics (means,	• In-depth interviews: thematic framework analysis<br>• Focus group discussions: thematic framework analysis	• Quantitative and qualitative data analyzed in parallel

For peer review only - http://bmjopen.bmj.com/site/about/guidelines.xhtml

	medians, standard deviations and frequencies)		
Interpretation	Merging of findings from quantitative and qualitative data collection strands to compare and contrast findings and provide recommendations on optimal feeding options and timing of growth monitoring in order to prevent infants from becoming nutritionally at-risk		

Study setting

The LIFE study is implemented in four sites across three countries: 1) Karnataka State, India and 2) Odisha State, India led by teams at Jawaharlal Nehru Medical College, JJM Medical College, SS Institute of Medical Sciences, City Hospital and Srirama Chandra Bhanja Medical College; 3) Lilongwe, Malawi led by the team at University of North Carolina (UNC) Project Malawi; and (4) Dar-es-Salaam, Tanzania led by the team at Muhimbili University of Health and Allied Sciences. All sites have strong, long-standing relationships with key government stakeholders, placing them in a position to address local and national priorities and advocate for translation of research to practice. Sites were chosen in South Asia and sub-Saharan Africa as these regions represent the greatest burden of LBW as well as the diverse drivers of LBW (2). Investigators will document regional similarities and differences in maternal and infant characteristics, gestational age, birthweight, feeding patterns, and growth. In total, participants are recruited from 12 study facilities (i.e. secondary and tertiary hospitals) chosen based on delivery volume, capacity to care for LBW infants in the first days of life, and willingness of facility leadership to participate. All facilities are located in urban settings with participants residing within a 50 kilometer radius. Additional site details are included in Table 2.

Table 2. Site descriptions

**Site**	**Prevalence of LBW (2,26)**	**Neonatal mortality rate (27)** *Deaths per 1000 live births*	**Infant mortality rate (28)**	**Study facilities** *Number and type by site*
India-Karnataka	17.2%	22	28	3 private tertiary hospitals<br>2 public tertiary hospitals
India-Odisha	20.8%			1 public tertiary hospital<br>1 public secondary hospital
Malawi	14.5%	20	31	1 public tertiary hospital<br>1 public secondary hospital
Tanzania	10.5%	20	36	1 public tertiary hospital<br>2 public secondary hospitals

Study population

Quantitative

For the quantitative data collection activities, the study population comprises mother-infant pairs that include newborns with recorded birthweights of 1.5kg to <2.5kg, as well as health facilities (Table 3). All 12 study facilities will participate in the facility needs assessments, including a facility profile and a donor human milk (DHM) bank assessment; further inclusion and exclusion criteria have not been specified.

For peer review only

For peer review only - http://bmjopen.bmj.com/site/about/guidelines.xhtml

Qualitative

For the qualitative data collection activities, the study population includes mothers, family members (e.g. husbands, guardians, mothers' parents, mother's in-laws, grandmothers, sisters, and sisters-in-law), religious and community leaders, traditional healers, clinicians, government officials, supply chain experts, and DHM banking experts meeting specific inclusion criteria (Table 3). Mothers and family members of newborns with recorded birthweights of 1.5kg to <2.5kg are eligible for the IDIs and FGDs. Clinicians who have been at their position for less than six months are not eligible.

Table 3. Inclusion and exclusion criteria for mothers-infant pairs

Data collection activity	Inclusion criteria	Exclusion criteria
Retrospective chart review	• Infants with birthweight of 1.5kg to <2.5kg<br>• Infants discharged before the start of prospective data collection LIFE	• Infants with birthweight <1.5kg<br>• Infants with congenital abnormalities that interfere with feeding (cleft lip or palate; hydrocephalus; gastrointestinal tract anomalies including gastroschisis, omphalocele, or anal atresia; neural tube defects; congenital cardiac defects; suspected trisomy 21; suspected TORCH (Toxoplasmosis, Other agents, Rubella, Cytomegalovirus, and Herpes simplex) infection<br>• Infants with young mothers: <18 years old in Tanzania and India, 16-17 years old and unmarried in Malawi, and all mothers <16 years old in Malawi<br>• Infants who die less than 72 hours from the time of birth<br>• Infants born outside the facility
Longitudinal prospective cohort	• Infants with birthweight between 1.5kg to <2.5kg (as measured at birth, or calculated using algorithm based on time since birth to account for expected postnatal weight loss)<br>• Mother-infant pairs who reside within the catchment area (approximately 50km) of the facility in which they were enrolled	• Infants with birthweight <1.5kg<br>• Infants with congenital abnormalities that interfere with feeding (cleft lip or palate; hydrocephalus; gastrointestinal tract anomalies including gastroschisis, omphalocele, or anal atresia; neural tube defects; congenital cardiac defects; suspected trisomy 21; suspected TORCH infection. Infants with severe neonatal encephalopathy jeopardizing early survival (as determined by modified Sarnat criteria) (29,30)<br>• Infants with young mothers: <18 years old in Tanzania and India, 16-17 years old and unmarried in Malawi, and all mothers <16 years old in Malawi<br>• Infants with mothers who died prior to enrollment<br>• Infants who die less than 72 hours from the time of birth<br>• Infants older than 72 hours at the time of screening<br>• Infants who withdraw less than 72 hours from the time of birth<br>• Infants with a twin or triplet who die prior to the time of screening<br>• Mothers who plan to leave the catchment area within 6 months of study enrollment
In-facility observational cohort	• Infants with birthweight between 1.5kg to <2.5kg	• Infants with birthweight <1.5kg<br>• Infants with congenital abnormalities that interfere with feeding<br>• Infants with young mothers: <18 years old in Tanzania and India, 16-17 years old and

For peer review only - http://bmjopen.bmj.com/site/about/guidelines.xhtml

		unmarried in Malawi, and all mothers <16 years old in Malawi<br>● Infants who die less than 6 hours from the time of birth<br>● Infants with mothers who die less than 6 hours from the time of birth<br>● Infants born outside the facility<br>● Infants older than 6 hours at the time of screening
In-depth interviews and focus group discussions (Birth - 6 months)	● Mothers with infants with birthweight between 1.5kg to <2.5kg aged 0 to 7 months (enrollment in prospective observational cohort not required)<br>● Family members of infants with birthweight between 1.5kg to <2.5kg aged 0 to 7 months who play a role in infant and young child feeding<br>● Religious leaders, community leaders, and traditional healers that are opinion leaders on infant and young child feeding practices in the community<br>● Health care workers currently involved in providing infant and young child feeding<br>● Government officials who support infant and young child feeding programs and policies<br>● Supply chain experts involved in infant and young child feeding supply chain logistics<br>● Human milk bank experts	● Mothers and family members with infants with birthweight ≥2.5kg<br>● Mothers and family members with infants with birthweight <1.5kg<br>● Young mothers: <18 years old in Tanzania and India, 16-17 years old and unmarried in Malawi, and all mothers <16 years old in Malawi<br>● Health care workers who have been in their position for less than 6 months<br>● Government officials who have been at their post for less than 6 months
In-depth interviews (9 - 12 months)	● Mothers with infants with birthweight between 1.5kg to <2.5kg enrolled in the prospective observational cohort and aged 9-12 months	● Mothers with infants who were not enrolled in the prospective cohort or withdrew/died before 9 months of age

For peer review only

Patient and public involvement

As part of the study design, the LIFE team involves clinicians, researchers, and community stakeholders familiar with the respective settings and populations. Study tools were piloted with patients and community members to ensure that research questions and indicators are culturally appropriate, acceptable, and relevant to the study population.

Study measures

Quantitative

We evaluate key maternal and infant characteristics (e.g., maternal education, maternal age, place of residence, parity, place and type of delivery, gestational age, infant sex and average length of facility stay) to better understand the LBW population in each site and region and to evaluate which characteristics serve as predictors of particular feeding patterns and growth and health outcomes. We examine infant feeding patterns including: early initiation of breastfeeding (within one hour of birth), feeding profiles (exclusive breastmilk, mixed milk feeding or no breastmilk) at each visit week from birth to 12 months, and duration of exclusive breastfeeding (feeding of only breastmilk directly from the breast, expressed or from a donor). We will assess infant growth by measuring weight, length, head circumference and mid-upper arm circumference (MUAC) at 13 timepoints. Anthropometrics will be used to identify stunting (length-for-age z-score <-2SD), wasting

For peer review only - http://bmjopen.bmj.com/site/about/guidelines.xhtml

(weight-for-length z-score <-2SD) and underweight (weight-for-age z-score <-2SD) at six and 12 months; and plot growth trajectories and velocities. Z-scores are derived from the International Fetal and Newborn Growth Consortium for the 21st Century (INTERGROWTH-21st) newborn size at birth and preterm postnatal growth standards (31,32) and the WHO infant growth standards (32) from birth through 12 months. We also examine weeks to birthweight regain, namely lack of regain in the first two weeks (33–35); and identify infants experiencing slow weight gain (<20g/ day) in the second week of life (infants are meant to gain an average of 20g/day in the first month of life and the second week of life is when infants should be gaining back their postnatal weight loss) (36,37). The WHO standards were designed using a cohort of term infants while the INTERGROWTH-21st standards were designed specifically for preterm infants and serve as a complement to the WHO standards in the first six months of life. Additional health outcomes include infant morbidity based on maternal self-report of illnesses and symptoms experienced by the infants in the past week at each study visit; neonatal and infant mortality at any point during follow-up; and child development assessed via the Caregiver Reported Early Childhood Development Instruments (CREDI) at one year of age (38). Maternal study measures including illness, depression (based on the Patient Health Questionnaire-2) and anthropometrics (weight, height and MUAC at birth, 6 weeks, 6 month and 12 months) are collected to evaluate mothers' roles in the feeding and growth of their infants (39). Finally, data on bed capacity, length of stay post-delivery, neonatal intensive care admissions, infrastructure, space, equipment, feeding options, medications, staffing, service availability, and sanitation practices are gathered to assess the health facility inputs available for care provision of LBW infants.

Qualitative

We explore beliefs, barriers, facilitators, risks and benefits of various feeding practices through structured IDIs and FGDs.

Data collection

Quantitative and qualitative data collection occur in parallel through six descriptive study activities (Table 1). For most data collection activities, timing of data collection will be linked with infant age (Figure 1). All data collection is prospective except for the chart reviews. Facility needs assessments are not included in Figure 1 since they will not be linked to infant age; facility profiles are completed at baseline and DHM assessments over the course of the study.

Figure 1. Data collection timeline by activity and infant age

Quantitative

Quantitative data collection is conducted by trained research nurses. The use of multiple data collection activities, combining observations and maternal self-report, helps to reduce bias and allows for triangulation of data. Retrospective patient chart data was collected for infants born in 12 study facilities between July 2018 and October 2019 using a structured survey. Chart reviews were completed before the initiation of prospective observational cohort data collection. The goal for the prospective cohort is to include 300 mother-infant pairs per site during 13 study visits using structured survey tools for infants born between September 2019 and January 2020 (noting that sites will start and end at different times) and completing one-year follow-up in July 2021 (Figure 1). Data are collected in-person at all timepoints, apart from at 32.5 and 45.5 weeks, at which time an abbreviated survey is administered over the phone in Malawi and Tanzania to bridge the three-month time gap between in-person visits in order to reduce loss to follow-up; India sites have previous experience

For peer review only - http://bmjopen.bmj.com/site/about/guidelines.xhtml

with at least three-month intervals between visits without adverse consequences on follow-up rates. Changes made in response to the COVID-19 pandemic included the addition of COVID-19 symptom screening questions, pauses to enrollment, data collection via phone calls where in-person visits were not possible and widening of the 6 months visit window. Table 4 summarizes the assessments and timing of administration for the prospective observational cohort. Focused training was conducted by a pediatrician or research investigator for all assessments listed. Although this is an observational cohort study with no intervention, adverse events are monitored and recorded. The in-facility observational cohort is intended to include 35 mother-infant pairs per site from all participating study facilities via regular feeding observations (15 minutes each) and maternal reports starting within six hours of birth and continuing until facility discharge (first week - every three to four hours; second week - one to two times a day; and third week and thereafter - once daily for unstable infants and every three days for stable infants) (Table 4). Finally, facility needs assessment data are collected via standardized tools capturing key vital statistics; the structural, human resource, equipment and service inputs present for new mothers and newborns; and facility and programmatic readiness for establishing and strengthening DHM banks (33–36). Study team members administer the assessments at each of the study facilities through observations, record reviews and staff consultations; global DHM experts will participate in and provide detailed guidance for the DHM component.

Table 4. Timing of assessments for longitudinal prospective observational cohort

**Assessment or survey tool content**	**Age of infant (weeks)**												
	**0**	**1**	**2**	**4**	**6**	**10**	**14**	**18**	**26**	**32.5**	**39**	**45.5**	**52**
Maternal demographics and pregnancy history*	·												
Infant demographics and delivery information*	·												
Dubowitz examination for gestational age at birth (40,41)	·												
Infant anthropometrics*	·	·	·	·	·	·	·	·	·		·		·
Maternal anthropometrics	·				·				·				·
Maternal and infant health information	·	·	·	·	·	·	·	·	·		·		·
Maternal and infant mortality information*	·	·	·	·	·	·	·	·	·	·	·	·	·
The World Health Organization-5 Well-Being Index (WHO-5) (42)											·		·
Maternal lactation and infant feeding information*	·	·	·	·	·	·	·	·	·	·	·	·	·
Infant and Young Child Feeding (IYCF) Questionnaire for complementary feeding period (43)											·		·

For peer review only - http://bmjopen.bmj.com/site/about/guidelines.xhtml

Latch, Audible Swallowing, Nipple Type, Comfort and Hold (LATCH) breastfeeding assessment* (44)		•	•	•	•	•	•	•	•				
Preterm Infant Breastfeeding Behavior Scale (PIBBS)* (45)		•	•	•	•								
Neonatal Eating Assessment Tool (NeoEAT) (46)		•	•	•	•	•	•	•	•				
Water, sanitation and hygiene information*		•	•	•	•	•	•	•	•		•		•
Patient Health Questionnaire 2 (PHQ-2) on maternal depression (47)		•	•	•	•	•	•	•	•		•		•
Caregiver Reported Early Childhood Development Instrument (CREDI) (38)													•

*Assessments also to be completed for the in-facility observational cohort between birth and facility discharge.

All sample sizes were determined to account for timeline, feasibility and resource constraints. Apart from the prospective observational cohort, we will not aim to make statistical inferences or precise point estimates from these samples, but will use the data descriptively for each infant to construct a narrative of their feeding patterns and health outcomes. The main statistical results from the prospective observational cohort will be point estimates and confidence intervals for certain rates, such as the percent of LBW infants who fail to thrive or whose growth falters, develop problems breastfeeding, or fall ill. The size of the true proportions will determine the precision of the estimate (e.g., with a sample of 300 mother-infant pairs at each site, a true proportion of 10% can be detected with precision of +/- 3.6%). The 95% confidence interval would be 6.4% to 13.6% (Figure 2).

Figure 2. Margin of error in estimation of a single proportion with a sample of 300 at each study site for the prospective observational cohort

To ensure standardization and facilitate high quality data collection, investigators will conduct site-specific workshops to review tools and train research staff on how to prepare for and conduct all key assessments; timing and duration will be tailored to the needs and knowledge of each team. Supervisors perform regular quality checks and refresher training as needed. Standardized anthropometric equipment includes: Seca 334 mobile digital baby scale, 887/876/874 digital flat scale with foot pedal for maternal weight, site-specific height boards for mothers, Seca 417 infant measuring board, Shorr mid-upper arm circumference (MUAC) tapes for infants and mothers (WM-MUAC26), and Shorr 65cm head circumference tapes for infants (SKU: WM-S Tape). The survey tools are administered in the local language (Hindi, Kannada and Marathi in India-Karnataka; Hindi and Oriya in India-Odisha; Chichewa in Malawi; and Swahili in Tanzania).

Qualitative

IDIs and FGDs with mothers and family members of LBW infants, community members, clinicians, government officials, and supply chain and DHM knowledge experts took place between July 2019 and January 2021. All participants will only be interviewed once. Interviewers include males and females with clinical and/or research backgrounds including specialized training in qualitative data collection methods. They conduct IDIs and FGDs with stakeholders and clinicians in English, and with mothers, family members and community leaders in relevant local languages. Interviewers are not personally known to interviewees and are trained to reduce bias

For peer review only - http://bmjopen.bmj.com/site/about/guidelines.xhtml

by building rapport with all participants and maintaining neutrality and confidentiality. They start each IDI and FGD by sharing the purpose of the study and any potential risks, obtaining consent, and then use structured interview guides with specific probes; guides were piloted prior to data collection. All IDIs and FDGs are audio-recorded with permission from participants and supported with real-time note-taking; transcripts and/or summary notes will not be reviewed by participants. IDIs are scheduled for one hour and FGDs for two. All qualitative data collection takes place in a private location either within a study health facility or in the community setting. Participants are given a small incentive to participate in order to cover the cost of travel and lost wages.

We use purposive sampling for government officials and knowledge experts and convenience sampling based on availability for clinicians, mothers, family members and community leaders. Participants are approached in person or via email and visits are confirmed over the phone. Mothers of LBW infants aged 0-3 months and 4-7 months are sampled from facility patient charts while those aged 9 and 12 months are sampled from the prospective cohort based on their sex and presence or absence of stunting at 6 months of age. Our sampling strategy is designed to reach code saturation rather than meaning saturation (48).

Data management and analysis

Quantitative

Quantitative data are entered and managed in CommCare (49), a secure electronic data capture and management system (retrospective charts review and prospective observational cohorts) and Excel (facility needs assessments). For the prospective observational cohort only, the Tanzania team uses its own electronic data capture system and securely transfers data to be merged with the rest of the data collected in CommCare. All data used for analysis are de-identified and stored securely. Experienced statistical analysts and epidemiologists will use means, medians and standard deviations to describe continuous variables and frequencies and proportions for categorical variables (all activities). For the prospective observational cohort, t-tests, chi-squared tests, and linear and logistic regression models will be used to explore relationships between key maternal and infant characteristics, feeding and growth. Confidence intervals around all measures will be constructed to adjust by site and cluster by mother to account for multiple births. Analyses will be stratified by site, sex and LBW phenotype. Missing data will be accounted for and denominators will be adjusted accordingly. Outliers for z-scores and anthropometrics will be examined and cleaned with the use of existing guidelines and sensitivity analyses, where helpful. Any further analyses will be post-hoc and described separately. Analyses will be conducted using the Stata Statistical Software Package, Version 16 (50) and the SAS statistical software suite (51).

Qualitative

De-identified qualitative data are stored for internal use in SharePoint, a secure web-based document management and storage system. A codebook will be developed deductively (based on the research aims and interview guide questions to inform high-level codes) and inductively (based on the emerging content of the IDIs and FGDs to inform sub-coded and emerging high-level codes) and applied to IDI and FGD data. Coding of the IDIs and FGDs will be performed by a total of seven coders and will involve a combination of rapid (framework analysis based on real-time notes supported by audio-recordings, where needed) and in-depth (coding of verbatim translated transcripts) approaches (52–54). Interviewers/site-based coders will employ a framework analysis and code notes into summary tables followed by subsequent coding (using a codebook) in Dedoose by qualitative researchers at Harvard. The first stage of data collection and coding in India and

For peer review only - http://bmjopen.bmj.com/site/about/guidelines.xhtml

Malawi will be used to enable on-going learning during the data collection process for a large volume of qualitative data (51). For maternal IDIs conducted among those with infants ages 9 months and older in India and Malawi, as well as all of Tanzania's IDIs and FGDs across the full year of infancy, interviewers will transcribe the audio-recordings verbatim in English and the qualitative researchers at Harvard will conduct a comprehensive, thematic analysis of the data to identify key messages as well as similarities and differences across sites. During the analysis, researchers at Harvard will review all coded data and will identify, via Dedoose, themes that were most and least commonly mentioned. Themes will be further defined and discussed with the site-based coders to review the interpretation of the data and reduce bias. Data will be analyzed by participant type and study location, and emergent key themes will be used to inform recommendations for future interventions.

Protocol and registration

The study is registered with clinicaltrials.gov (NCT04002908) and CTRI/2019/02/017475 (Clinical Trial Registry of India - http://ctri.nic.in).

ETHICS & DISSEMINATION

This study was approved by 11 ethics committees in India, Malawi, Tanzania and the United States: 1) India Health Ministry's Screening Committee with Indian Council of Medical Research acting as its secretariat (2019-2674); 2) Directorate of Health and Family Welfare Services, Government of Karnataka which also covers investigators at Women & Children Hospital, Davangere and Chigateri General District Hospital, Davangere (NHM/SPM/04/2019-20); 3) Institutional Ethics Committee of KLE Academy of Higher Education and Research which also covers investigators at JN Medical College, Belagavi and KLES Dr Prabhakar Kore Hospital & Medical Research Center, Belagavi (KAHER/IEC/2019-20/D-2760); 4) Institutional Ethics Review Board of SS Institute of Medical Sciences & Research Centre (IERB/200/2019); 5) Institutional Ethics Committee of JJM Medical College (JJMMC/IEC-01/2019) which also covers investigators at Bapuji Child Health Institute & Research Centre, Davangere, Women & Children Hospital, Davangere and Chigateri General District Hospital, Davangere; 6) Research and Ethics Committee, Directorate of Health Services, Odisha State which also covers investigators at City Hospital Oriya Bazar, Cuttack (155/PMU/187/17); 7) Institutional Ethical Committee, Sriram Chandra Bhanja Medical College, Cuttack (7188); 8) Malawi National Health Sciences Research Committee (NHSRC2019/Protocol19/03/2250-UNCPM 21905); 9) Tanzania National Institute of Medical Research (NIMR/HQ/R.8a/Vol.IX/3126); 10) Muhimbili University of Health and Allied Sciences (DA.282/298/01.C/); and 11) United States - Harvard T.H Chan School of Public Health (IRB10-0282) which also covers investigators at Boston Children's Hospital, Brigham and Women's Hospital, Emory University, PATH and University of North Carolina.

Written informed consent is obtained from all IDI, FGD, in-facility observational cohort and prospective observational cohort participants**.** After six months of follow-up, prospective observational cohort participants will be re-consented for an additional six months. Verbal consent will only be obtained in the event of extenuating circumstances where written consent cannot be sought; IRB approval will be needed before proceeding. For the facility needs assessments, verbal consent is sought at the facility leadership level as these activities are considered to be a part of quality improvement. Results from the LIFE study will be disseminated at global- and site-levels through peer-reviewed publications and presentations to key stakeholders during meetings and conferences. Site investigators will also share results informally with participating study facilities. Upon publication of the study results, select data will be made publicly available via Harvard Dataverse (55).

For peer review only - http://bmjopen.bmj.com/site/about/guidelines.xhtml

DISCUSSION

The LIFE study will fill critical data gaps in the care and nutrition of LBW infants in LMICs; although limited, most of the existing research in this area is concentrated in high-income countries (18). Overall, evidence is lacking on feeding, care and health of LBW infants born with birthweights of 1.5 to <2.5kg; however, these moderately LBW infants represent the majority (>90%) of global LBW births compared to very LBW infants (<1.5kg) (2,18,56–59). We will aim to establish the foundational knowledge required to design, test and implement the most effective and feasible infant feeding strategies to prevent and address growth faltering among LBW infants in low resource settings. The LIFE study will provide the much-needed evidence to comprehensively understand what LBW infants require, how they differ from infants born ≥2.5 kg, and what rigorous research is critical to strengthen global LBW infant feeding guidelines.

ACKNOWLEDGEMENTS

The authors would like to thank clinical leadership and staff at all study facilities for their partnership, support and contribution to this work; the mothers and infants for allowing us to have a glimpse into their experiences and sharing key moments of their lives; community members, government officials and subject experts for sharing their perspectives; and all data collectors and study staff for conducting study activities.

AUTHORS' CONTRIBUTIONS

Study conceptualization and design was completed by ACL, BAC, CD, CRS, DET, EB, IH, KEAS, KIB, KLM, KM, KMi, KN, LA, LSp, LSu, LV, MFY, NH, RMB, SMD, SSG and TM. Protocol development and tool design was carried out by ACL, BAC, CRS, DET, EB, EF, GG, IH, JNB, KEAS, KF, KIB, KLM, KM, KMi, KN, LA, LD, LGS, LSp, LSu, LV, MFY, MMD, NH, NS, RK, RMB, SLM, SM, SP, SMD, SSG, SSS, SSV and TM. Tool pilot testing/ modification were conducted by EB, EF, FS, GG, JNB, KF, LD, LGS, LSp, LSu, LV, MP, NH, NS, SM, SP, SSS and SSV. Writing of the manuscript was conducted by LSp and LV. All authors reviewed multiple drafts of the manuscript and approved the final version.

FUNDING STATEMENT

This work was supported by The Bill & Melinda Gates Foundation, grant number OPP1192260. The Bill and Melinda Gates Foundation reviewed the study design and sample-size calculations, but will not be involved in data collection, management, analysis, interpretation, writing of the manuscript, or the decision to submit manuscripts for publication.

COMPETING INTERESTS STATEMENT

All authors completed the ICMJE conflict of interest form and were funded by the Gates Foundation for this work as part of the LIFE study. ACL, BC, CRS, DET, KEAS, KIB, KLM, KM, MMD, RK and SS have received funding from the Gates Foundation for maternal and newborn health work at large. CD reports other from American Society for Nutrition, other from UpToDate, and other from People's Medical Publishing House outside the submitted work. ACL reports grants from the World Health Organization and National Institute of Health NICHD outside the submitted work. All other authors have declared no conflicts of interest.

DATA AVAILABILITY STATEMENT

For peer review only - http://bmjopen.bmj.com/site/about/guidelines.xhtml

For peer review only

Data sharing not applicable at this time as no datasets have been generated and/or analyzed for this study.

REFERENCES

1. Goal 3: Ensure healthy lives and promote well-being for all at all ages — SDG Indicators. United Nations - SDG; 2017.
2. Blencowe H, Krasevec J, de Onis M, Black RE, An X, Stevens GA, et al. National, regional, and worldwide estimates of low birthweight in 2015, with trends from 2000: a systematic analysis. Lancet Glob Health. 2019 May 15;7(7):e849–60.
3. Liu L, Oza S, Hogan D, Chu Y, Perin J, Zhu J, et al. Global, regional, and national causes of under-5 mortality in 2000-15: an updated systematic analysis with implications for the Sustainable Development Goals. Lancet. 2016 Dec 17;388(10063):3027–35.
4. Imdad A, Bhutta ZA. Nutritional management of the low birth weight/preterm infant in community settings: a perspective from the developing world. J Pediatr. 2013 Mar;162(3 Suppl):S107-14.
5. Blencowe H, Cousens S, Oestergaard MZ, Chou D, Moller A-B, Narwal R, et al. National, regional, and worldwide estimates of preterm birth rates in the year 2010 with time trends since 1990 for selected countries: a systematic analysis and implications. Lancet. 2012 Jun 9;379(9832):2162–72.
6. Katz J, Lee AC, Kozuki N, Lawn JE, Cousens S, Blencowe H, et al. Mortality risk in preterm and small-for-gestational-age infants in low-income and middle-income countries: a pooled country analysis. Lancet. 2013 Aug 3;382(9890):417–25.
7. Lee ACC, Katz J, Blencowe H, Cousens S, Kozuki N, Vogel JP, et al. National and regional estimates of term and preterm babies born small for gestational age in 138 low-income and middle-income countries in 2010. Lancet Glob Health. 2013 Jul;1(1):e26-36.
8. Mwaniki MK, Atieno M, Lawn JE, Newton CRJC. Long-term neurodevelopmental outcomes after intrauterine and neonatal insults: a systematic review. Lancet. 2012 Feb 4;379(9814):445–52.
9. Lawn JE, Kerber K, Enweronu-Laryea C, Cousens S. 3.6 million neonatal deaths--what is progressing and what is not? Semin Perinatol. 2010 Dec;34(6):371–86.
10. Pawar SJ, Oleti T, Bharathi S, Tipparaju S, Mustafa E. Growth and Neurodevelopmental Outcome in Preterm LBW Infants with Sepsis in India: A Prospective Cohort. Int J Pediatr. 2018 Feb 21;2018:5735632.
11. Upadhyay RP, Martines JC, Taneja S, Mazumder S, Bahl R, Bhandari N, et al. Risk of postneonatal mortality, hospitalisation and suboptimal breast feeding practices in low birthweight infants from rural Haryana, India: findings from a secondary data analysis. BMJ Open. 2018 Jun 22;8(6):e020384.
12. Rana R, McGrath M, Gupta P, Thakur E, Kerac M. Feeding Interventions for Infants with Growth Failure in the First Six Months of Life: A Systematic Review. Nutrients. 2020 Jul 9;12(7).
13. Christian P, Lee SE, Donahue Angel M, Adair LS, Arifeen SE, Ashorn P, et al. Risk of childhood undernutrition related to small-for-gestational age and preterm birth in low- and middle-income countries. Int J Epidemiol. 2013 Oct;42(5):1340–55.
14. Lawn JE, Kerber K, Enweronu-Laryea C, Massee Bateman O. Newborn survival in low resource settings--are we delivering? BJOG. 2009 Oct;116 Suppl 1:49–59.
15. Wardlaw TM, World Health Organization, UNICEF. Low birthweight: Country, regional and global

For peer review only - http://bmjopen.bmj.com/site/about/guidelines.xhtml

For peer review only

estimates. Geneva: WHO; 2004.

16. Blanc AK, Wardlaw T. Monitoring low birth weight: an evaluation of international estimates and an updated estimation procedure. Bull World Health Organ. 2005 Mar 16;83(3):178–85.

17. Gisore P, Shipala E, Otieno K, Rono B, Marete I, Tenge C, et al. Community based weighing of newborns and use of mobile phones by village elders in rural settings in Kenya: a decentralised approach to health care provision. BMC Pregnancy Childbirth. 2012 Mar 19;12:15.

18. North K, Marx-Delaney M, Bose C, Lee AC, Vesel L, Adair L, et al. Effect of Milk Type and Fortification on Growth of Low-Birthweight Infants: An Umbrella Review of Systematic Reviews and Meta-Analyses. Matern Child Nutr. 2021 Jul;17(3):e13176.

19. Guidelines on Optimal Feeding of Low Birth-Weight Infants in Low- and Middle-Income Countries. Geneva: World Health Organization; 2011.

20. Brown S, McSharry P. Improving accuracy and usability of growth charts: case study in Rwanda. BMJ Open. 2016 Jan 27;6(1):e009046.

21. URGENT CALL FOR AN INVESTIGATION INTO THE FEEDING OF SICK & VULNERABLE NEWBORNS | by JustACTIONS | Medium [Internet]. [cited 2021 Jun 24]. Available from: https://medium.com/@JustACTIONS/urgent-call-for-an-investigation-into-the-feeding-of-sick-vulnerable-newborns-3f4ddb49c494.

22. WHO. Survive and thrive: transforming care for every small and sick newborn. Geneva, Switzerland: World Health Organization; 2018.

23. Strengthening the reporting of observational studies in epidemiology (STROBE) statement: guidelines for reporting observational studies. BMJ. 2007 Nov 3;335(7626).

24. Tong A, Sainsbury P, Craig J. Consolidated criteria for reporting qualitative research (COREQ): a 32-item checklist for interviews and focus groups. Int J Qual Health Care. 2007 Dec;19(6):349–57.

25. John W. Creswell and Vicki L. Plano Clark. Designing and Conducting Mixed Methods Research. 3rd ed. 2017.

26. IIPS. National Family Health Survey 2015-2016. Mumbai: International Institute for Population Sciences; 2017.

27. Countdown Country Profiles [Internet]. Countdown to 2030. [cited 2020 Oct 30]. Available from: https://profiles.countdown2030.org/#/ds/MWI.

28. IGME. IGME UNInter-agency Group for Child Mortality Estimation [Internet]. 2020 [cited 2020Dec 7]. Available from: https://childmortality.org/.

29. Sarnat HB, Sarnat MS. Neonatal encephalopathy following fetal distress. A clinical and electroencephalographic study. Arch Neurol. 1976 Oct;33(10):696–705.

30. Roland EH, Hill A. Clinical aspects of perinatal hypoxic-ischemic brain injury. Semin Pediatr Neurol. 1995 Mar 1;2(1):57–71.

31. Villar J, Giuliani F, Bhutta ZA, Bertino E, Ohuma EO, Ismail LC, et al. Postnatal growth standards for preterm infants: the Preterm Postnatal Follow-up Study of the INTERGROWTH-21(st) Project. Lancet Glob Health. 2015 Nov;3(11):e681-91.

32. Villar J, Cheikh Ismail L, Victora CG, Ohuma EO, Bertino E, Altman DG, et al. International standards for newborn weight, length, and head circumference by gestational age and sex: the Newborn Cross-Sectional Study of the INTERGROWTH-21st Project. Lancet. 2014 Sep 6;384(9946):857–68.

For peer review only - http://bmjopen.bmj.com/site/about/guidelines.xhtml

33. Noel-Weiss J, Courant G, Woodend AK. Physiological weight loss in the breastfed neonate: a systematic review. Open Med. 2008 Oct 28;2(4):e99–110.

34. Macdonald PD, Ross SRM, Grant L, Young D. Neonatal weight loss in breast and formula fed infants. Arch Dis Child Fetal Neonatal Ed. 2003 Nov;88(6):F472-6.

35. DiTomasso D, Cloud M. Systematic Review of Expected Weight Changes After Birth for Full-Term, Breastfed Newborns. J Obstet Gynecol Neonatal Nurs. 2019 Nov;48(6):593–603.

36. Fenton TR, Anderson D, Groh-Wargo S, Hoyos A, Ehrenkranz RA, Senterre T. An Attempt to Standardize the Calculation of Growth Velocity of Preterm Infants-Evaluation of Practical Bedside Methods. J Pediatr. 2018 May;196:77–83.

37. Homan GJ. Failure to thrive: A practical guide. Am Fam Physician. 2016 Aug 15;94(4):295–9.

38. McCoy DC, Sudfeld CR, Bellinger DC, Muhihi A, Ashery G, Weary TE, et al. Development and validation of an early childhood development scale for use in low-resourced settings. Popul Health Metr. 2017 Feb 9;15(1):3.

39. Kroenke K, Spitzer RL, Williams JBW. The Patient Health Questionnaire-2: Validity of a two-item depression screener. Med Care. 2003 Nov;41(11):1284–92.

40. Clopton N. The dubowitz assessment of gestational age. Phys Occup Ther Pediatr. 1983;3(3):75–86.

41. Dubowitz LM, Dubowitz V, Goldberg C. Clinical assessment of gestational age in the newborn infant. J Pediatr. 1970 Jul;77(1):1–10.

42. Topp CW, Østergaard SD, Søndergaard S, Bech P. The WHO-5 Well-Being Index: a systematic review of the literature. Psychother Psychosom. 2015 Mar 28;84(3):167–76.

43. World Health Organization. Indicators forassessing infant and young childfeeding practices. Part 1 Definitions. [Internet]. World Health Organization; 2008 [cited 2020 Dec 18]. Available from: https://apps.who.int/iris/bitstream/handle/10665/43895/9789241596664_eng.pdf?sequence=1.

44. Jensen D, Wallace S, Kelsay P. LATCH: a breastfeeding charting system and documentation tool. J Obstet Gynecol Neonatal Nurs. 1994 Jan;23(1):27–32.

45. Hedberg Nyqvist K, Ewald U. Infant and maternal factors in the development of breastfeeding behaviour and breastfeeding outcome in preterm infants. Acta Paediatr. 1999 Nov;88(11):1194–203.

46. Pados BF, Estrem HH, Thoyre SM, Park J, McComish C. The neonatal eating assessment tool: development and content validation. Neonatal Netw. 2017 Nov 1;36(6):359–67.

47. Manea L, Gilbody S, Hewitt C, North A, Plummer F, Richardson R, et al. Identifying depression with the PHQ-2: A diagnostic meta-analysis. J Affect Disord. 2016 Oct;203:382–95.

48. Hennink MM, Kaiser BN, Marconi VC. Code saturation versus meaning saturation: how many interviews are enough? Qual Health Res. 2017 Mar;27(4):591–608.

49. Dimagi. CommCare. Cambridge, MA: Dimagi; 2021.

50. StataCorp LLC. Stata Statistical Software. College Station, TX: StataCorp LLC; 2019.

51. SAS Institute. SAS Software Suite. Cary, NC: SAS Institute; 2020.

52. Gale RC, Wu J, Erhardt T, Bounthavong M, Reardon CM, Damschroder LJ, et al. Comparison of rapid vs in-depth qualitative analytic methods from a process evaluation of academic detailing in the Veterans Health Administration. Implement Sci. 2019 Feb 1;14(1):11.

53. Taylor B, Henshall C, Kenyon S, Litchfield I, Greenfield S. Can rapid approaches to qualitative analysis

For peer review only - http://bmjopen.bmj.com/site/about/guidelines.xhtml

deliver timely, valid findings to clinical leaders? A mixed methods study comparing rapid and thematic analysis. BMJ Open. 2018 Oct 8;8(10):e019993.

54. Gale NK, Heath G, Cameron E, Rashid S, Redwood S. Using the framework method for the analysis of qualitative data in multi-disciplinary health research. BMC Med Res Methodol. 2013 Sep 18;13:117.

55. Harvard Dataverse [Internet]. [cited 2021 Jun 24]. Available from: https://dataverse.harvard.edu/.

56. World Health Organization, United Nations Children's Fund (UNICEF). Low birthweight: country, regional and global estimates. World Health Organization; 2004.

57. O'Leary M, Edmond K, Floyd S, Newton S, Thomas G, Thomas SL. A cohort study of low birth weight and health outcomes in the first year of life, Ghana. Bull World Health Organ. 2017 Aug 1;95(8):574–83.

58. Mahumud RA, Sultana M, Sarker AR. Distribution and determinants of low birth weight in developing countries. J Prev Med Public Health. 2017 Jan;50(1):18–28.

59. Marete I, Ekhaguere O, Bann CM, Bucher SL, Nyongesa P, Patel AB, et al. Regional trends in birth weight in low- and middle-income countries 2013-2018. Reprod Health. 2020 Dec 17;17(Suppl 3):176.

For peer review only

For peer review only - http://bmjopen.bmj.com/site/about/guidelines.xhtml

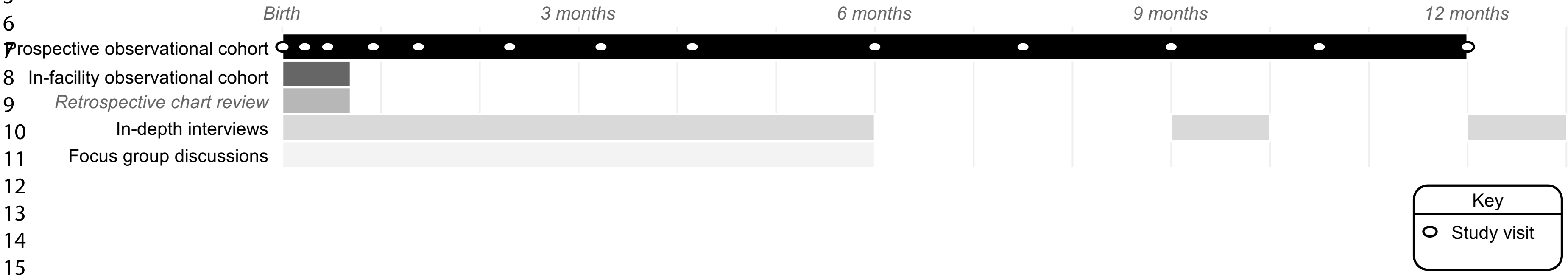

Figure 1. Data collection timeline by activity and infant age

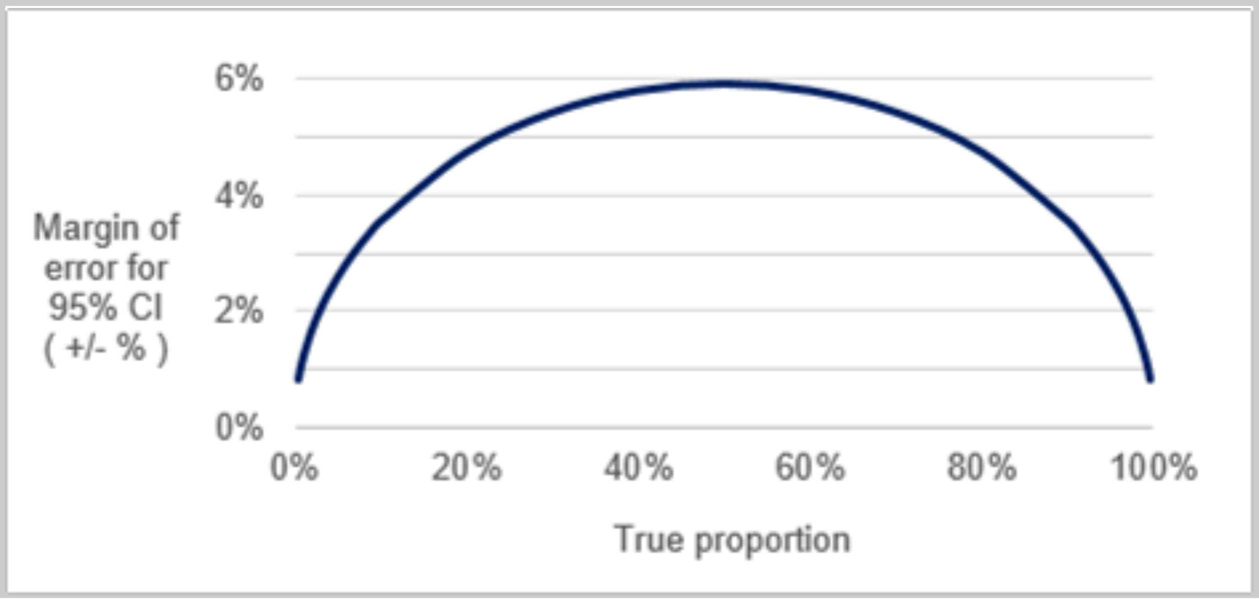

Figure 2. Margin of error in estimation of a single proportion with a sample of 300 at each study site for the prospective observational cohort

For peer review only

Consolidated criteria for reporting qualitative studies (COREQ): 32-item checklist

Developed from:
Tong A, Sainsbury P, Craig J. Consolidated criteria for reporting qualitative research (COREQ): a 32-item checklist for interviews and focus groups. *International Journal for Quality in Health Care*. 2007. Volume 19, Number 6: pp. 349 – 357

YOU MUST PROVIDE A RESPONSE FOR ALL ITEMS. ENTER N/A IF NOT APPLICABLE

No. Item	Guide questions/description	Reported on Page #
**Domain 1: Research team and reflexivity**		
Personal Characteristics		
1. Interviewer/facilitator	Which author/s conducted the interview or focus group?	N/A – The authors did not conduct the interviews or focus groups
2. Credentials	What were the researcher's credentials? E.g. PhD, MD	Page 11
3. Occupation	What was their occupation at the time of the study?	Page 11
4. Gender	Was the researcher male or female?	Page 11
5. Experience and training	What experience or training did the researcher have?	Page 11
Relationship with participants		
6. Relationship established	Was a relationship established prior to study commencement?	Page 11-12
7. Participant knowledge of the interviewer	What did the participants know about the researcher? e.g. personal goals, reasons for doing the research	Page 11-12
8. Interviewer characteristics	What characteristics were reported about the interviewer/facilitator? e.g. Bias, assumptions, reasons and interests in the research topic	Page 11
**Domain 2: study design**		
Theoretical framework		
9. Methodological orientation and theory	What methodological orientation was stated to underpin the study? e.g. grounded theory, discourse analysis, ethnography, phenomenology, content analysis	Pages 4-5
Participant selection		
10. Sampling	How were participants selected? e.g. purposive, convenience, consecutive, snowball	Pages 6-8 & 11-12
11. Method of approach	How were participants approached? e.g.	Page 12

For peer review only

	face-to-face, telephone, mail, email	
12. Sample size	How many participants were in the study?	Page 5
13. Non-participation	How many people refused to participate or dropped out? Reasons?	N/A because this is a protocol
Setting		
14. Setting of data collection	Where was the data collected? e.g. home, clinic, workplace	Page 12
15. Presence of non-participants	Was anyone else present besides the participants and researchers?	N/A because this is a protocol
16. Description of sample	What are the important characteristics of the sample? e.g. demographic data, date	Pages 4-8
Data collection		
17. Interview guide	Were questions, prompts, guides provided by the authors? Was it pilot tested?	Page 11-12
18. Repeat interviews	Were repeat interviews carried out? If yes, how many?	Page 11
19. Audio/visual recording	Did the research use audio or visual recording to collect the data?	Page 12
20. Field notes	Were field notes made during and/or after the inter view or focus group?	Page 12
21. Duration	What was the duration of the interviews or focus group?	Page 12
22. Data saturation	Was data saturation discussed?	Page 12
23. Transcripts returned	Were transcripts returned to participants for comment and/or correction?	Page 12
**Domain 3: analysis and findings**		
Data analysis		
24. Number of data coders	How many data coders coded the data?	Page 12
25. Description of the coding tree	Did authors provide a description of the coding tree?	N/A – this is a protocol and data has not yet been coded
26. Derivation of themes	Were themes identified in advance or derived from the data?	Page 12-13
27. Software	What software, if applicable, was used to manage the data?	Page 13
28. Participant checking	Did participants provide feedback on the findings?	Page 12-13
Reporting		
29. Quotations presented	Were participant quotations presented to illustrate the themes/findings? Was each quotation identified? e.g. participant number	N/A – this is a protocol
30. Data and findings consistent	Was there consistency between the data presented and the findings?	N/A – this is a protocol
31. Clarity of major themes	Were major themes clearly presented in the findings?	N/A – this is a protocol
32. Clarity of minor themes	Is there a description of diverse cases or discussion of minor themes?	N/A – this is a protocol

For peer review only

For peer review only

STROBE Statement—Checklist of items that should be included in reports of ***cohort studies***

	Item No	Recommendation	Page No
**Title and abstract**	1	(*a*) Indicate the study's design with a commonly used term in the title or the abstract<br>(*b*) Provide in the abstract an informative and balanced summary of what was done and what was found	a) Title: Page 1<br>Abstract: Page 2<br>b) Page 2. Please note that this is a protocol paper so there are no results reported.
**Introduction**			
Background/rationale	2	Explain the scientific background and rationale for the investigation being reported	Page 3
Objectives	3	State specific objectives, including any prespecified hypotheses	Introduction: Page 4<br>Table 1: Pages 4-6
**Methods**			
Study design	4	Present key elements of study design early in the paper	Study design: Pages 4<br>Table 1: Page 5
Setting	5	Describe the setting, locations, and relevant dates, including periods of recruitment, exposure, follow-up, and data collection	Study setting: Page 6<br>Table 2: Page 6<br>Data collection: Pages 9-12<br>Table 4: Pages 10-11
Participants	6	(*a*) Give the eligibility criteria, and the sources and methods of selection of participants. Describe methods of follow-up<br>(*b*) For matched studies, give matching criteria and number of exposed and unexposed	a) Study population: Pages 6-8<br>Table 3: Pages 7-8<br>Data collection: Pages 9-12<br>b) Not applicable; this is not a matched study
Variables	7	Clearly define all outcomes, exposures, predictors, potential confounders, and effect modifiers. Give diagnostic criteria, if applicable	Study measures: Pages 8-9<br>Data management and analysis: Pages 12-13
Data sources/ measurement	8*	For each variable of interest, give sources of data and details of methods of assessment (measurement). Describe comparability of assessment methods if there is more than one group	Study measures: Page 8-9<br>Data collection: Pages 9-12
Bias	9	Describe any efforts to address potential sources of bias	Data collection: Pages 9 and 11
Study size	10	Explain how the study size was arrived at	Data collection: Page 11<br>Figure 2: Page 11
Quantitative variables	11	Explain how quantitative variables were handled in the analyses. If applicable, describe which groupings were chosen and why	Data management and analysis: Pages 12-13. However, this is a protocol paper so no analyses have been conducted.
Statistical methods	12	(*a*) Describe all statistical methods, including those used to control for confounding<br>(*b*) Describe any methods used to examine subgroups and interactions	a-c) Data management and analysis: Pages 12-13<br>d) Data collection: Page 9<br>e) Data management and analysis: Page 12

For peer review only - http://bmjopen.bmj.com/site/about/guidelines.xhtml

		(*c*) Explain how missing data were addressed<br>(*d*) If applicable, explain how loss to follow-up was addressed<br>(*e*) Describe any sensitivity analyses	
**Results**			Not applicable because this is a protocol
Participants	13*	(a) Report numbers of individuals at each stage of study—eg numbers potentially eligible, examined for eligibility, confirmed eligible, included in the study, completing follow-up, and analysed<br>(b) Give reasons for non-participation at each stage<br>(c) Consider use of a flow diagram	Not applicable - protocol
Descriptive data	14*	(a) Give characteristics of study participants (eg demographic, clinical, social) and information on exposures and potential confounders<br>(b) Indicate number of participants with missing data for each variable of interest<br>(c) Summarise follow-up time (eg, average and total amount)	Not applicable - protocol
Outcome data	15*	Report numbers of outcome events or summary measures over time	Not applicable - protocol

For peer review only

For peer review only - http://bmjopen.bmj.com/site/about/guidelines.xhtml

Main results	16	(*a*) Give unadjusted estimates and, if applicable, confounder-adjusted estimates and their precision (eg, 95% confidence interval). Make clear which confounders were adjusted for and why they were included<br>(*b*) Report category boundaries when continuous variables were categorized<br>(*c*) If relevant, consider translating estimates of relative risk into absolute risk for a meaningful time period	Not applicable - protocol
Other analyses	17	Report other analyses done—eg analyses of subgroups and interactions, and sensitivity analyses	Not applicable - protocol
**Discussion**			
Key results	18	Summarise key results with reference to study objectives	Not applicable – protocol. We have summarized the utility and anticipated outputs in the discussion on page 13-14.
Limitations	19	Discuss limitations of the study, taking into account sources of potential bias or imprecision. Discuss both direction and magnitude of any potential bias	Strengths and limitations page 2. To be further discussed in results paper.
Interpretation	20	Give a cautious overall interpretation of results considering objectives, limitations, multiplicity of analyses, results from similar studies, and other relevant evidence	Not applicable - protocol
Generalisability	21	Discuss the generalisability (external validity) of the study results	Strengths and limitations page 2. To be further discussed in results paper.
**Other information**			
Funding	22	Give the source of funding and the role of the funders for the present study and, if applicable, for the original study on which the present article is based	Funding statement: Page 14

*Give information separately for exposed and unexposed groups.

Note: An Explanation and Elaboration article discusses each checklist item and gives methodological background and published examples of transparent reporting. The STROBE checklist is best used in conjunction with this article (freely available on the Web sites of PLoS Medicine at http://www.plosmedicine.org/, Annals of Internal Medicine at http://www.annals.org/, and Epidemiology at http://www.epidem.com/). Information on the STROBE Initiative is available at http://www.strobe-statement.org.

For peer review only

For peer review only - http://bmjopen.bmj.com/site/about/guidelines.xhtml
